# Changes in the gene expression profiles of the brains of male European eels (*Anguilla anguilla*) during sexual maturation

**DOI:** 10.1186/1471-2164-15-799

**Published:** 2014-09-17

**Authors:** Allison M Churcher, Jose Martin Pujolar, Massimo Milan, Peter C Hubbard, Rute ST Martins, João L Saraiva, Mar Huertas, Luca Bargelloni, Tomaso Patarnello, Ilaria AM Marino, Lorenzo Zane, Adelino VM Canário

**Affiliations:** CCMAR- Centre for Marine Sciences, University of Algarve, Campus de Gambelas, 8005-139 Faro, Portugal; Department of Bioscience, Aarhus University, Aarhus, 8000 Denmark; Department of Comparative Biomedicine and Food Science, University of Padova, Viale dell’ Università 16, 35020 Legnaro, PD Italy; Department of Biology, University of Padova, Via G. Colombo 3, 35131 Padova, PD Italy

**Keywords:** Eel, Neuroendocrine, Brain, Gene expression, Reproduction, Microarray

## Abstract

**Background:**

The vertebrate brain plays a critical role in the regulation of sexual maturation and reproduction by integrating environmental information with developmental and endocrine status. The European eel *Anguilla anguilla* is an important species in which to better understand the neuroendocrine factors that control reproduction because it is an endangered species, has a complex life cycle that includes two extreme long distance migrations with both freshwater and seawater stages and because it occupies a key position within the teleost phylogeny. At present, mature eels have never been caught in the wild and little is known about most aspects of reproduction in *A. anguilla*. The goal of this study was to identify genes that may be involved in sexual maturation in experimentally matured eels. For this, we used microarrays to compare the gene expression profiles of sexually mature to immature males.

**Results:**

Using a false discovery rate of 0.05, a total of 1,497 differentially expressed genes were identified. Of this set, 991 were expressed at higher levels in brains (forebrain and midbrain) of mature males while 506 were expressed at lower levels relative to brains of immature males. The set of up-regulated genes includes genes involved in neuroendocrine processes, cell-cell signaling, neurogenesis and development. Interestingly, while genes involved in immune system function were down-regulated in the brains of mature males, changes in the expression levels of several receptors and channels were observed suggesting that some rewiring is occurring in the brain at sexual maturity.

**Conclusions:**

This study shows that the brains of eels undergo major changes at the molecular level at sexual maturity that may include re-organization at the cellular level. Here, we have defined a set of genes that help to understand the molecular mechanisms controlling reproduction in eels. Some of these genes have previously described functions while many others have roles that have yet to be characterized in a reproductive context. Since most of the genes examined here have orthologs in other vertebrates, the results of this study will contribute to the body of knowledge concerning reproduction in vertebrates as well as to an improved understanding of eel biology.

**Electronic supplementary material:**

The online version of this article (doi:10.1186/1471-2164-15-799) contains supplementary material, which is available to authorized users.

## Background

Animal reproduction depends on the precise timing and coordination of behavioral and physiological processes. For successful reproduction to occur, animals need to reach the appropriate developmental stage, locate and attract mates and respond appropriately to reproductively relevant signals. The accuracy of each aspect of reproduction is essential to ensure that genetic material is passed on to the next generation.

A major function of the brain is to establish the appropriate developmental and endocrine status for reproduction, and then to co-ordinate this with aspects of behavior and sexual maturation. In vertebrates, this coordination is established through the hypothalamus and pituitary gland [1]. The pituitary gland secretes follicle-stimulating hormone (FSH) and luteinizing hormone (LH) which are gonadotropins that act on the gonads to induce sex steroid synthesis and promote gametogenesis. The pituitary gonadotropes are in the vicinity of neuron endings that release neuropeptides and neurotransmitters, such as gonadotropin releasing-hormone (GnRH) and dopamine, which stimulate and inhibit gonadotropin secretion respectively. Following gonadotropin release, sex steroids produced by gonad feedback to the brain and pituitary provide information about reproductive status [2, 3].

Brain and pituitary gene expression changes that are associated with reproduction have been observed in several teleosts. These include changes in the pattern of GnRH expression at maturation in masu salmon *Oncorhynchus masou* [4, 5], European sea bass *Dicentrarchus labrax* [6], red seabream *Pagrus major* [7], goldfish [8] and European eel *Anguilla anguilla* [9]. Changes in the expression levels of LHβ and FSHβ gene subunits have also been observed in the pituitary of several species, including European sea bass and European eel [9, 10]. Additionally, changes in the levels of kisspeptin system genes have also been observed in several teleosts [11, 12], as have changes in aromatase expression and activity [13, 14]. Many of these gene expression changes occur in areas of the brain that are associated with the detection and processing of sensory information.

Due to its complex life cycle and extreme long distance migrations, *A. anguilla* is an intriguing model in which to investigate reproduction related changes in the brain. The European eel is believed to spawn in a single location in the Sargasso Sea [15]. After spawning, larvae are transported by surface currents in the Gulf Stream to the shores of Europe and North Africa. When larvae reach the continental shelf, they undergo metamorphosis into glass eels that migrate into fresh, brackish and coastal waters as yellow eels. After a highly variable feeding period of 5–30 years, yellow eels metamorphose into silver eels that migrate from their widely dispersed foraging areas to the common spawning ground in the Sargasso Sea. This migration is approximately 5,000-6,000 km long. The changes that occur during metamorphosis into silver eels include an increase in eye size, a change in body coloration and a regression of the digestive system. Eels do not feed during the transatlantic adult migration and rely exclusively on fat content for energy. After reaching the spawning grounds in the Sargasso Sea, eels reproduce once then die shortly thereafter [15]. *A. anguilla* is also an important species to better understand the molecular aspects of reproduction because it is an endangered species, is of interest for aquaculture purposes and occupies a key position within the teleost phylogeny.

While much progress has been made over the last century, many aspects of eel reproduction remain poorly understood, including the physiological and molecular mechanisms responsible for sexual maturation and the coordination of reproductive events [16]. European eels are sexually immature when they start their migration across the Atlantic to the spawning grounds; it is not clear if they mature sexually during migration or after they arrive at Sargasso Sea. Maturation experiments through hormonal induction have shown dramatic changes at the physiological level (e.g. an increase gonad size, an increase in the production of sex steroids and other sex hormones, and the progression of gonadogenesis from the immature stage to the gamete producing stage) as well as changes in secondary sexual characteristics (e.g. darkened skin, increased ocular indices, and soft swollen abdomens). Final maturation in eels does not appear to be triggered by swimming alone and is likely influenced by exposure to environmental cues as the eels swim across the Atlantic [16]. In addition, reproductive pheromones are believed to have a role in the regulation of final maturation and reproduction in eels [17, 18]. If sexual maturation in *A. anguilla* is triggered by environmental signals, then signal detection is expected to occur through sensory pathways and signal processing is expected to occur in the brain before the neuroendocrine response is activated.

At present, mature adult eels have never been caught in the wild and eels do not mature in captivity without treatment with gonadotropic hormones. The inability to reach sexual maturation in captivity is due to a lack in sufficient pituitary gonadotropin production [19]. In eels, maturation is under double neuroendocrine control which occurs in some teleosts [2, 3] and which can be reversed by gonadotropic hormone treatments [9, 20, 21]. The dopaminergic inhibitory process leads to insufficient GnRH stimulation, and to the inhibition of pituitary gonadotropes FSH and LH, which exert direct and indirect control on gonad development by acting on steroid metabolism [3, 22].

The goal of the present gene profiling study was to identify changes in the brain that are involved in the coordination of reproductive events in the European eel, including those involved in neuroendocrine regulation of gametogenesis. More specifically, genes involved in sexual maturation and reproduction are expected to be expressed at higher levels in the brain of sexually mature males compared to immature males. Additionally, since the coordination of reproductive events is influenced by environmental cues that must be integrated with the physiological and developmental status of the animal, it is expected that changes at the molecular level occur in the areas associated with the processing of sensory signals (e.g. telencephalon, diencephalon and olfactory bulb).

## Results

Using a false discovery rate of 0.05, a total of 1,497 differentially expressed genes were identified. Of this set, 991 were expressed at higher levels in brains (forebrain and midbrain) of sexually mature males while 506 were expressed at lower levels relative to brains of sexually immature males. When searched against the RefSeq proteins using BLASTX [23], these transcripts retrieved 732 and 389 unique sequences, respectively.

### Functional annotation using DAVID

To obtain a list of gene identifiers that could be used for the functional analysis of differentially expressed genes, the *A. anguilla* transcriptome assembly contigs that were used to design the microarray were used in a BLASTX search of SwissProt proteins. The result of this search revealed that 11,130 of the 13,523 transcripts had alignments with e-values of 0.01 or less in SwissProt. When the contigs without hits in SwissProt were searched against RefSeq proteins and then the nr database, alignments for additional 1,149 and 110 sequences were retrieved, respectively. Using this approach, sequence identifiers for a total of 12,389 contigs were retrieved. For the analyses with DAVID [24], only differentially expressed transcripts with BLAST alignment e-values of 0.001 or less were considered.

**Figure 1:**
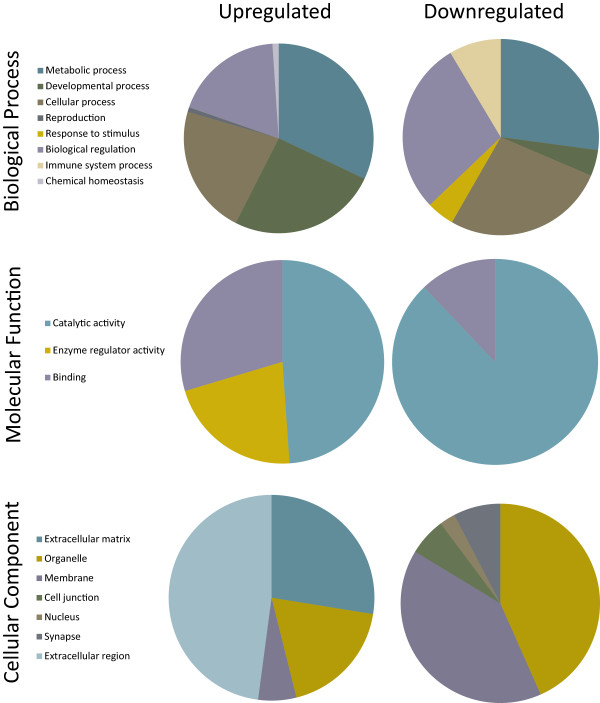
**Proportion of differentially expressed terms belonging to the GO categories Biological Process, Molecular Function and Cellular component.** Enriched terms from the lists of significantly up and down-regulated genes (q values of less than 0.05) were identified using DAVID. For each of the three main GO categories, terms were assigned to subcategories.

Functional annotation analysis with DAVID uncovered a number of significantly enriched terms within the list of up- and down-regulated genes (Additional file 1: Table S1). Figure 1 details the proportion of over-represented terms belonging to the following GO categories: Biological process, Cellular component and Molecular Function. For each of the three main GO categories, terms were assigned to subcategories.

Cluster analysis with DAVID identified eight significantly enriched clusters of up-regulated genes and five significantly enriched clusters of down-regulated genes (Additional file 2: Table S2 and Additional file 3: Table S3). For this analysis, only clusters with EASE scores greater than or equal to 1.3 were considered. Clusters of up-regulated genes include many that are involved in DNA binding, calcium singling and nervous system development and differentiation. Clusters of down-regulated genes include genes for RNA processing, immune system function and nucleotide binding.

### Differential expression: keyword analysis

To identify specific sets of differentially expressed genes, the protein descriptions obtained from the BLASTX search of the eukaryotic proteins in RefSeq were used. In total, 12,269 out of 13,523 assembly contigs showed alignments with an e-value score of 0.01 or better in the RefSeq proteins. The complete list of differentially expressed genes can be found in Additional file 4: Table S1. Among this set, we identified 64 neuroendocrine genes that are differentially expressed in the brains of sexually mature male eels (Table 1).

**Table 1:** **Differentially expressed neuroendocrine genes in the brains of sexually mature and immature male eels**

Direction	Fold change	Description	Function	GI number
+	1.99	Calcium/calmodulin-dependent protein kinase kinase 2-like isoform X1	Calcium signaling	498924960
+	1.52	Calcium-binding protein 4	Calcium signaling	62901497
+	1.79	Calmodulin-like	Calcium signaling	499017111
+	2.25	Cocaine- and amphetamine-regulated transcript ch22 precursor	Feeding, neuroendocrine signaling, neuronal development	325652192
+	2.73	Cocaine- and amphetamine-regulated transcript protein	Feeding, neuroendocrine signaling, neuronal development	301610675
+	3.85	Cocaine- and amphetamine-regulated transcript protein-like	Feeding, neuroendocrine signaling, neuronal development	410898200
+	2.12	Collagen alpha-2(V) chain	Calcium, collagen	143811378
+	1.67	Corticotropin-releasing factor-binding protein precursor	Neuroendocrine signaling	51010999
+	4.4	C-type natriuretic peptide 1 precursor	Neuropeptide signaling, energy metabolism	317575753
+	4.35	C-type natriuretic peptide 1-like	Neuropeptide signaling, energy metabolism	348526229
+	2.51	C-type natriuretic peptide 3-like precursor	Neuropeptide signaling, energy metabolism	192455608
+	1.56	Developmentally-regulated GTP-binding protein 2-like isoform X1	Development	1706518
+	1.67	Fibroblast growth factor receptor 1	Regulation of development, differentiation and migration	120047
+	9.78	Galanin peptides-like isoform 1	Neuroendocrine signaling	348532269
+	3.75	Glutamate decarboxylase 1-like	Neurotransmitter biosynthesis (GABA synthesis)	348538615
+	2.03	Growth arrest-specific protein 1	Developmental protein	30923264
+	2.02	Heat shock 70 kDa protein-like	Chaperone	326665952
+	2.02	heat shock 70 kDa protein-like	Chaperone	2495346
+	1.9	High affinity cGMP-specific 3'',5''-cyclic phosphodiesterase 9A	GPCR signaling, neuronal processes	326670632
+	4.02	Homeobox protein HMX2	Neurogenesis and differentiation	27735196
+	2	Homeobox protein HMX2	Neurogenesis and differentiation	152032526
+	2.48	Homeobox protein SIX1	Developmental protein, transcription regulation	2495290
+	1.97	Immunoglobulin superfamily containing leucine-rich repeat protein 2 precursor	Neurogenesis and differentiation	61806620
+	5.28	Insulin gene enhancer protein ISL-3-like	Neuroendocrine signaling	348500124
+	4.62	Iroquois-class homeodomain protein irx-2	Neurogenesis and differentiation	82181198
+	2.11	Iroquois-class homeodomain protein IRX-3	Developmental protein, transcription regulation	47117874
+	4.89	Iroquois-class homeodomain protein irx-3	Neurogenesis and differentiation	82185674
+	5.5	Iroquois-class homeodomain protein IRX-3	Developmental protein, transcription regulation	262527548
+	1.54	Iroquois-class homeodomain protein IRX-5	Neurogenesis and differentiation	143811406
+	1.63	Mammalian ependymin-related protein 1 precursor	Cell matrix adhesion	50539892
+	1.63	Mammalian ependymin-related protein 1-like	Cell matrix adhesion	499037681
+	2.46	Membrane-associated progesterone receptor component 1	Neuroendocrine signaling	318102138
+	1.78	Metalloproteinase inhibitor 2	Protease inhibitor	267133
+	2.47	Natriuretic peptide precursor C-like protein precursor	Neuropeptide signaling	238624212
+	1.83	Neuroendocrine protein 7B2-like isoform 2	Neuropeptide signaling	292624780
+	2.14	Neurogenic differentiation factor 1	Neurogenesis and differentiation	82227588
+	6.39	Neuropeptide B-like	Feeding, neuroendocrine signaling, neuronal development	348536652
+	2.22	Neuropeptide Y-like	Feeding, Neuroendocrine signaling	327274623
+	1.68	Neuropeptide-like protein C4orf48 homolog precursor	Developmental protein	213510972
+	2.53	Prodynorphin precursor	Neuropeptide signaling	317574783
+	2.4	Protachykinin-1 precursor	Neuropeptide signaling	317575728
+	2.57	Stanniocalcin-1	Calcium signaling	505851525
+	2.67	Tachykinin 3a precursor	Neuropeptide signaling	373838776
+	1.64	Transcription factor HES-4	Retinogenesis, neurogenesis and differentiation	82202542
+	2.53	Transcription factor HES-5	Developmental protein, transcription regulation	3913838
+	2.29	Type I iodothyronine deiodinase	Thyroid hormone biosynthesis	348504600
+	5.03	Type III iodothyronine deiodinase a	Thyroid hormone biosynthesis	209954616
+	9.85	Type III iodothyronine deiodinase b	Thyroid hormone biosynthesis	209954618
+	1.51	Zinc finger protein ZIC 1	Neurogenesis and differentiation	82243612
+	1.9	Zinc finger protein ZIC 2-A	Neurogenesis and differentiation	223634664
-	0.59	Calcium/calmodulin-dependent protein kinase (CaM kinase) II alpha	Calcium signaling	154147642
-	0.62	Calcium/calmodulin-dependent protein kinase II inhibitor 2	Calcium signaling	223029393
-	0.65	Calmodulin	Calcium signaling	224004208
-	0.48	Cyclic AMP-dependent transcription factor ATF-3	Transcription regulation	41055726
-	0.42	Cytochrome P450 3A18	Hydroxylation of testosterone	21955148
-	0.66	G protein-coupled receptor kinase 4-like	GPCR signaling	292609536
-	0.58	Glutamate receptor subunit epsilon-2	Ion transport	190358411
-	0.51	Heat shock 90 kDa protein 1 beta isoform b	Chaperone	185136252
-	0.6	Heat shock protein HSP 90-beta-like	Chaperone	392356281
-	0.63	Insulin receptor substrate 2-B-like	Insulin signaling	348540607
-	0.27	Insulin receptor substrate 2-B-like	Insulin signaling	504156832
-	0.41	Interferon-induced transmembrane protein 5-like	Bone mineralization	432860359
-	0.61	Lipopolysaccharide-induced tumor necrosis factor-alpha factor homolog	Transcription regulation	348534997
-	0.64	Prodynorphin precursor	Neuropeptide signaling	213514964

Genes that have positive direction values were up-regulated in the brains of sexually mature male eels in comparison to sexually immature male eels while those that are negative were down-regulated in this group. Gene descriptions are based on BLASTX hits in RefSeq proteins. A false discovery rate of 0.05 was used to identify differentially expressed genes.

The results of the DAVID analysis suggest that some re-organization of signaling pathways in the brain occurs at sexual maturation. Therefore, it seemed likely that there would also be changes in the expression levels for genes that are involved directly in cell-cell communication such as receptors and channels. We therefore searched for up and down regulated channels and G protein-coupled receptors (GPCRs). Seven GPCRs were differentially expressed. Four of these belong to the Glutamate family of GPCRs [25], one belongs to the Rhodopsin-like family and two belong to the Secretin family (Table 2). One of the Glutamate family receptors, an extracellular calcium sensing receptor-like gene, was expressed at higher levels in the brains of sexually mature males. The other three were expressed at lower levels in the brains of sexually mature males compared with sexually immature males. The receptor from the Rhodopsin-like GPCR family was the serotonin receptor (5-hydroxytryptamine receptor 1B) which was expressed at higher levels in the brains of sexually mature males than in immature males (Table 2). The GPCR 64-like gene and the GPCR 125-like gene belong to the Secretin family and were expressed at higher levels in the brains of sexually mature males. Additionally, there were eleven differentially expressed channels (Table 2) seven of which were expressed at higher levels in the brains of sexually mature males in comparison to the brains of immature males.

**Table 2:** **Differentially expressed GPCRs and channels in the brains of sexually mature male eels**

Direction	Fold change	Protein description	Type
+	1.66	Extracellular calcium-sensing receptor-like [Oreochromis niloticus]	GPCR (Glutamate)
+	2.07	5-hydroxytryptamine receptor 1B-like [Oryzias latipes]	GPCR (Rhodopsin)
+	6.15	G-protein coupled receptor 64-like [Maylandia zebra]	GPCR (Secretin)
+	2.37	Probable G-protein coupled receptor 125-like [Maylandia zebra]	GPCR (Secretin)
-	0.64	Probable G-protein coupled receptor 148-like [Oreochromis niloticus]	GPCR (Glutamate)
-	0.50	Metabotropic glutamate receptor 5-like [Danio rerio]	GPCR (Glutamate)
-	0.59	Metabotropic glutamate receptor 5-like [Oreochromis niloticus]	GPCR (Glutamate)
+	3.20	Sodium channel protein type 4 subunit alpha B-like [Maylandia zebra]	Channel
+	1.91	Sodium channel protein type 2 subunit alpha-like isoform 7 [Gallus gallus]	Channel
+	1.60	Voltage-dependent anion-selective channel protein 1 [Danio rerio]	Channel
+	1.74	Voltage-dependent anion-selective channel protein 2 [Salmo salar]	Channel
+	1.66	Voltage-dependent anion-selective channel protein 2 [Danio rerio]	Channel
+	1.60	Gamma-aminobutyric acid receptor subunit delta-like [Oreochromis niloticus]	Channel
+	5.68	Cyclic nucleotide-gated cation channel alpha-3-like [Danio rerio]	Channel
-	0.64	Potassium voltage-gated channel subfamily C member 2-like [Oreochromis niloticus]	Channel
-	0.63	Voltage-gated potassium channel subunit beta-1-like [Danio rerio]	Channel
-	0.62	Calcium-activated potassium channel subunit alpha-1-like [Xenopus tropicalis]	Channel
-	0.50	Sodium channel protein type 2 subunit alpha-like partial [Takifugu rubripes]	Channel

G protein-coupled receptors (GPCRs) were assigned to families based on the GRAFS classification system [25]. Genes that have positive direction values were up-regulated in the brains of sexually mature male eels in comparison to immature males while those that are negative were down-regulated in this group. Gene descriptions are based on BLASTX hits in RefSeq proteins. A false discovery rate of 0.05 was used to identify differentially expressed genes.

### Quantitative polymerase chain reaction

**Figure 2:**
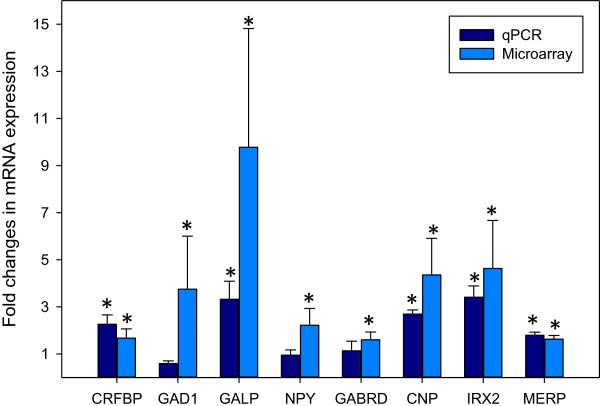
**Comparison of qPCR results with the microarray results for eight differentially expressed genes.** The expression levels for eight differentially expressed genes were determined by quantitative polymerase chain reaction (qPCR) with the same RNA pools that were used for the microarray. These include the following differentially expressed genes: galanin peptide (GALP), glutamate decarboxylase 1 (GAD1), corticotropin releasing factor binding protein (CRFBP), C-type natriuretic peptide (CNP), gamma-aminobutyric acid receptor subunit δ (GABRD), neuropeptide Y (NPY), mammalian ependymin-related protein (MERP) and the iroquois-class homeodomain protein 2 (IRX2). Bars represent mean fold changes, error bars represent standard deviations and * represents significant (P < 0.05) increases in the level of expression in the brains of sexually mature male eels in comparison to immature male eels.

Eight differentially expressed genes were chosen for quantitative polymerase chain (qPCR) using the same RNA samples that were used for the microarray (Figure 2). This list includes: galanin peptide (GALP), glutamate decarboxylase 1 (GAD1), corticotropin releasing factor binding protein (CRFBP), C-type natriuretic peptide (CNP), gamma-aminobutyric acid receptor subunit δ (GABRD), neuropeptide Y (NPY), mammalian ependymin-related protein (MERP) and the iroquois-class homeodomain protein 2 (IRX2). The TATA box binding protein (TBP) and elongation factor 1-alpha 1 (EEF1A1) were used as controls.

To verify that the fold changes observed in the microarray experiments correlated with the fold changes observed in the qPCR experiments, Spearman’s rank order correlation was used. The results of this test indicate a positive relationship exists between the fold changes observed in the microarray and qPCR experiments (n = 24, r = 0.511, p = 0.011). The qPCR expression levels between immature and mature samples were further evaluated using Student's t-tests. GALP, CRFPB, CNP, MERP and IRX2 were expressed at significantly higher levels in the brains of mature males compared with mature (P < 0.05) while the differences in expression of NPY, GAD1 and GABDR were below the statistical significance level.

## Discussion

This study shows that major changes in the gene expression profiles occur in the brains of male European eels at sexual maturity. This shift includes an increase in the expression levels of genes involved in cell signaling, including receptor and channels, as well as genes involved in development and differentiation. Several neuroendocrine genes were also expressed at higher levels in the brains of mature males in comparison to the brains of immature males. These genes are likely involved in gonadal maturation and development and act through the brain-pituitary-gonad axis. Genes involved in immune system function were down-regulated in the brains of sexually mature males, suggesting that a re-allocation of resources occurs at this stage. Interestingly, the expression levels of several receptors and channels change at sexual maturity suggesting that some neuronal rewiring occurs at this stage.

More transcripts for neuropeptide Y (NPY) were observed in the brains of mature male eels in comparison to immature males. NPY is a short peptide whose expression pattern has been described in the brains of several teleosts. For example, in goldfish, NPY expression has been detected in the forebrain including the telencephalon, preoptic area, the olfactory bulb and thalamic regions [26]. In the Japanese eel *Anguilla japonica*, NPY is highly expressed in the telencephalon, optic tectum thalamus and hypothalamus [27]. NPY stimulates LH release in fish [28], has a role in growth hormone (GH) and gonadotropin hormone (GTH) secretion [29–31] and may coordinate growth, feeding and reproduction in fish [2]. In goldfish, fasting and exposure to sex steroids increases NPY mRNA levels in the brain [32, 33]. Furthermore, the NPY response in goldfish depends on reproductive status [34]. Food deprivation in *A. japonica* also causes increases of NPY mRNA levels in the brain [27]. Changes in feeding behavior and regulation of energy expenditure are expected to accompany the maturation process in fish, particularly in species such as *A. anguilla* that allocate a lot of resources to reproduction [35]. Since the European eel does not feed at sexual maturity, it is possible that increased levels of NPY are related to nutritional and developmental status.

Genes involved in gamma-aminobutyric acid (GABA) signaling were also expressed at higher levels in the brains of mature males. For example, glutamate decarboxylase 1 (GAD1), an enzyme involved in the production of GABA from L-glutamic acid, was more abundant in the brains of sexually mature than immature males. GABA is a neurotransmitter involved in the control of pituitary hormone secretion in teleosts [36]. GABA has a stimulatory effect on LH by enhancing GnRH release and inhibiting dopamine turnover [2, 37]. The GABA A receptor subunit δ (GABRD) was expressed at a higher level in the brains of sexually mature male eels than in immature males. GABA A receptors are hetero-pentamer ligand gated channels that are gated by GABA binding. It appears that different combinations of receptor subunits may confer functional specificity [38]. In goldfish, four GABA A subunits (α 1, β 4, γ 1 and 2) were expressed at higher levels in the brains of sexually mature females [39]. Similarly, the GABA A receptor γ 2 subunit was expressed at higher levels in the brains of goldfish exposed to prostaglandin-F_2α_ (PGF_2α_) than in controls [40]. In the current study, the δ subunit was up-regulated in sexually mature males; this is intriguing given that the δ subunit appears to be involved in neurosteroid modulation of the GABA A receptor [41].

In the brains of sexually mature males, the 5-hydroxytryptamine (serotonin) receptor 1B was expressed at a higher level than in immature males. The brain serotonergic system is one of the mechanisms used to cope with the dramatic changes during maturation. This system plays an important role in a complex neuroendocrine loop that functions in homeostasis and acclimation during physiological or environmental challenges [42, 43].

Corticotropin releasing factor binding protein (CRFBP) was also expressed at a higher level in the brains of mature males than in immature males. CRFBP has an inhibitory role on CRF which is a regulator of stress responses [44]. In fish, acute stress results in an increase in CRFBP expression in the hypothalamus [45]. For European eels, reproduction is a stressful event because the spawning migration journey is very long (5,000-6,000 km). In addition, it is presumably challenging for both sexes to coordinate the temporal and spatial aspects of spawning in the vast Sargasso Sea which is 1,000 km wide and 3,000 km long. At the mature stage, European eels are likely to reduce their threshold for assessing risks and threats since they have a single opportunity to reproduce. Therefore the observed increases in CRFBP may be part of the stress response and of changes in behavior that are associated with reproduction.

An increase in the levels of mammalian ependymin-related protein (MERP) in the brains of mature males was observed. MERPs belong to a phylogenetically diverse clade that is related to ependymins [46] which are glycoproteins that are found in abundance in the cerebrospinal fluid of fish. Ependymins are involved in synaptic changes during memory consolidation and regeneration of the optic nerve [47–49]. Changes in the levels of ependymin-II expression have been observed in the hypothalamus in goldfish at breeding season [39] and in the telencephalon following exposure to a female pheromone [40].

Several genes involved in calmodulin (CALM) signaling were differentially expressed in the brains of sexually mature males (Table 1). For example, CALM is involved in calcium signaling and nervous system function including memory consolidation [50] and was up-regulated in the brains of sexually mature male eels. In goldfish, CALM levels also change with reproductive status. During breeding season, CALM and calcium/calmodulin-dependant protein kinase 2 (CaMK2) levels are highest in the hypothalamus in female goldfish [39] and CALM levels are highest in the telencephalon of males following exposure to PGF_2α_ [40]. Sexual maturation alters the eel’s internal state and changes in the way the animal relates to its environment. In the European eel, the memory circuitry is expected to be important for reproduction due to the long migration across the Atlantic to the Sargasso Sea. Though CaM signaling genes are likely involved in many processes in the brains of mature eels, it is possible that the changes observed here are also related to memory.

The microarray results showed a high positive correlation with those obtained in selected genes by qPCR thus providing a global validation of the microarray results. NPY, GAD1 and GABDR did not seem to follow the same pattern; this could be related to the low expression levels of these genes (and the associated higher error rate of measurement) or to the existence of alternative transcripts that are differentially detected by the two methods. However, these possibilities were not further investigated.

Recent studies in fish show that specific cells in the brain respond to light and exert neuroendocrine control of testis development [51, 52]. In *A. anguilla*, behavioral evidence indicates that photoreceptor sites that are independent of the pineal exist in the brain [53]. Eels become more sexually mature after months of migration in deep waters while only surfacing at night. In *A. anguilla*, spawning is believed to occur in the top 200 meters [16] where there is more light than in deeper water. The results of the current study show that several genes with roles in photosignal transduction and visual system development were up-regulated in the brains of mature males. This list includes retinal rod rhodopsin-sensitive cGMP 3' 5'-cyclic phosphodiesterase subunit γ and cyclic nucleotide-gated cation channel α-3 which is involved in olfactory and visual signal transduction. It also includes the calcium binding protein 4, which has an important role in synaptic function in photoreceptor cells [54] and the iroquois-class homeodomain protein (IRX5). IRX5 is a transcription factor that is involved in cone cell differentiation and retinal, craniofacial and gonad development [55, 56]. The transcription factor HES4 was also up-regulated in the brains of sexually mature males. HES4 belongs to a family of genes that have roles in cell differentiation and has a role in retina development [57]. While it is possible that these genes have other roles in the brains of mature males, an intriguing avenue for future research would be to determine whether there is a link between neurohormone systems, gonad development and photoreceptors in the brains of *A. anguilla* like there is in masu salmon [52].

Several genes involved in neurogenesis, cellular differentiation and development were up-regulated in the brain of sexually mature males (Additional file 1: Table S1). This set includes genes from the GO categories such as GO:0050767 (regulation of neurogenesis), GO:0007411 (axon guidance), GO:0045664 (regulation of neuron differentiation) and GO:0007423 (sensory organ development). In addition, many differentially expressed ion channels and GPCRs were differentially expressed (Table 2) which suggests that some re-organization and/or re-growth occurs at maturation. The calcium-activated potassium channel would tend to hyperpolarize (inhibit) neurons in response to neuromodulators/neurotransmitters. Down-regulation of these channels suggests a less responsive, more ‘hard-wired’ brain, which may be advantageous for certain aspects of reproduction.

GnRH, aromatase, kisspeptin system genes and sex steroid receptors are also known to have roles in reproduction. The microarray included a probe for one isoform of GnRH1 and no differences in expression were observed between immature and mature males. The array did not contain probes for other GnRH genes or isoforms. Although GnRH1 controls gonadotrophin secretion in the pituitary [58], the fact that the fish were artificially induced to mature using human chorionic gonadotrophin suggests that no positive or negative feedback mechanisms towards GnRH1 were occurring at the time of sampling. The microarray also contained probes for transcripts that are similar to the estrogen-related receptor beta like 1 and the androgen receptor beta and no significant differences in expression were detected. Kisspeptin system genes and aromatase were not analyzed in this survey. At present, mature eels have never been caught in the wild and the only way to obtain sexually mature animals is through artificial induction of maturation. It is therefore not clear to what extent changes that occur at the molecular level in the brain are the result of specific gonadal feedback mechanisms.

The eyes and the olfactory epithelium change with sexual maturation in *A. anguilla* and the American eel *Anguilla rostrata* [59, 60] suggesting that there is a change in the use of sensory modalities with reproduction. As mentioned previously, the data from this study suggest that the forebrains of male eels undergo some sort of rewiring at sexual maturation. As the areas of the brain tested here are associated with the processing and conversion of sensory input to physiological responses, it is possible that changes in the brain at sexual maturity are influenced by changes in sensory system requirements that occur at reproduction.

## Conclusions

In this study, we used nearly 15,000 microarray probes to identify gene expression changes in the brains of eels that occur at sexual maturation. Of the set of differentially expressed genes, 991 were expressed at higher levels in sexually mature males while 506 were expressed at lower levels than in immature males. This finding shows that the brains of eels undergo major changes at the molecular level during reproduction. The set of up-regulated genes includes genes that are involved in neuroendocrine processes which likely act through the brain-pituitary-gonad axis. It also includes genes involved in cell-cell signaling, neurogenesis and development indicating that the brain is undergoing developmental changes that are associated with maturation and reproduction. A major advantage to the approach employed here is that it allows many genes to be tested at the same time. It also allows for the identification of genes not previously known to be involved in particular processes such as reproduction. Consequently, some of these differentially expressed genes identified here have roles that have been described in other studies while others have roles that have yet to be characterized. Furthermore, since the majority of the genes tested here have orthologs in other vertebrates, the results from this study can be used to understand reproduction across a diversity of vertebrate species. Most importantly however, is that we have defined a set of genes that appear to be important for sexual maturation in *A. anguilla*. These findings therefore contribute to the body of knowledge concerning eel biology and will, in the future, contribute to aquaculture and conservation efforts.

## Methods

### Sample preparation

Approximately four hundred sexually immature European eels (80–90 g/fish) were obtained from a local distributor (Pescafial, Seville, Spain) and kept for acclimation in a 2000 L tank with freshwater (0 PSU salinity) and under natural temperature and photoperiod. After 14 days, approximately 120 fish were transferred to two independent temperature controlled recirculation water units connected to two 500 L cylindro-conical tanks (30 fish per tank) where the fish were acclimated to seawater (35 PSU salinity) over two weeks. Recirculation units were kept at a 20-22°C at 12 L/12D photoperiod and the fish were fed with commercial pellets (DIBAQ No. 3, Proaqua, Spain) at 3% of body weight per day. Males in one of the seawater units were injected intramuscularly every week for 140 days with 2000 IU hCG/kg (human chorionic gonadotropin, Sigma–Aldrich Chemical) in 0.9% saline to induce sexual maturation. Males in the other recirculation unit were injected weekly over the same period with 0.9% NaCl (vehicle).

Finally, eels were anesthetized in a solution of 100 mg/L tricaine methanesulfonate (MS-222, Sigma Aldrich) then decapitated. Tissues (brain, including the olfactory bulbs, telencephalon, diencephalon and mesencephalon) were collected from sexually immature (n = 12) and sexually mature males (n = 12) and frozen immediately in liquid nitrogen until the tissue collections were complete. They were then placed in RNAlater® (Sigma R0901) and kept overnight at 4°C then transferred to -20°C. Each tissue sample was pooled with the tissue from two other samples. Therefore each replicate (n = 4 mature, n = 4 immature) contains tissue from three individuals. Sexual maturation was characterized as follows [61]: seawater immature fish were designated stage III (presence of spermatogonia and spermatocytes), whereas sexually mature males were designated stage VI (presence of only spermatozoa and few spermatogonia which is called the functional maturation stage). The average gonadosomal index (GSI) values for sexually mature and immature males were 8.41 ± 0.56% and 0.084 ± 0.01% respectively. The mean eye diameters for mature males were 8.19 ± 0.16 mm and 6.51 ± 0.13 mm for immature males. Brain samples were shipped on dry ice. Fish care and experimentation complied with national legislation for the use of laboratory animals under a group-1 license issued by the Directorate-General for Veterinary, Ministry of Agriculture, Rural Development and Fisheries of Portugal.

### Microarray analysis

RNA was isolated from the brain using the RNAeasy Mini Kit (Qiagen) following the manufacter’s protocol. RNA was extracted separately for olfactory bulbs, telencephalons and diencephalon/mesencephalons. Afterwards, RNA concentration was measured using a Nanodrop ND-100 spectrophotometer (NanoDrop Technologies) and RNA integrity and quality were estimated using an Agilent 2100 Bioanalyzer (Agilent Technologies). A minimum RNA integrity number (RIN) of 7.5 was considered. Because RNA concentrations of olfactory bulb, telencephalon and diencephalon were below the amount needed for microarray analysis, a separate experiment for each part of the brain was not possible. Therefore, we combined equal concentrations of olfactory bulb, telencephalon and diencephalon/mesencephalon as a single brain sample for each replicate. Each replicate therefore contains RNA from the olfactory bulb, telencephalon and diencephalon/mesencephalon from three individuals.

Microarray analysis was conducted using an European eel-specific array consisting of a total of 14,913 probes based on a large collection of high-throughput transcriptomic sequences [62]. Probe sequences and further details on the microarray platform can be found on the GEO database under accession number GPL15124.

Sample labelling and hybridization were conducted following the details in [63]. Hybridized slides were scanned at 5 μm resolution using an Agilent DNA microarray scanner. Slides were scanned at two different sensitivity levels (XDR Hi 100% and XDR Lo 10%) to increase the power to detect both lowly and highly expressed genes. The two linked images generated were analyzed together. Data were extracted and background subtracted using the standard procedure in Agilent Feature Extraction (FE) software v. 9.5.1. Hybridization success was evaluated using flag values, excluding those intensities not equal to 1. Data was normalized using a quantile normalization procedure using R (http://www.r-project.org). Normalized fluorescence data from the arrays have been deposited in the GEO database (http://www.ncbi.nlm.nih.gov/geo) under accession number GSE55858.

Differentially transcribed genes across samples were identified using the program SAM (Significance Analysis of Microarrays) version 4.0 [63]. Groups (mature vs. immature males) were compared using the two-class unpaired test and up and down regulated genes were identified. Only genes with a minimum fold change of 1.5 were considered.

### Functional annotation using DAVID

To obtain a list of gene identifiers that could be used for the functional analysis of differentially expressed genes, BLASTX (2.2.25+ suite) [23] was used to search SwissProt. For this analysis, the SwissProt database was chosen to maximize the number of sequence identifiers that DAVID version 6.7 [24] would recognize. In this search, the European eel transcriptome assembly contigs (n = 13,523) that were used to design the array [62] were used as queries. Assembly contigs that did not have a BLASTX hit in SwissProt meeting the e-value cutoff of 0.01 were then searched against RefSeq proteins. Those without hits in SwissProt or RefSeq were then searched against the National Center for Biotechnology Information (NCBI) nr database using the same e-value cutoff.

For functional annotation analysis with DAVID, only BLAST alignments with an e-value less than or equal to 0.001 were considered. To identify differentially expressed genes, a false discovery rate (q-value) of 0.05 was used. For the DAVID analysis, the standard default settings that include an EASE score of 0.1 were used. For the functional annotation analysis, the annotation categories selected were: GO terms, Interpro, KEGG pathways and SP_PIR keywords. For the cluster analysis with DAVID, only clusters with EASE scores of greater than or equal to 1.3 were considered. To obtain a more descriptive view of up and down-regulated processes, GO terms belonging to the three GO categories (Biological Processes, Cellular Cycle and Molecular Function) were further divided into subcategories.

### Differential expression: keyword analysis

To identify specific subsets of genes that are differentially expressed in the brains of sexually mature male eels, the set of annotations from a BLASTX search of the RefSeq eukaryotic proteins (n = 3,360,895) was used. This approach was chosen because RefSeq, like SwissProt, is a curated dataset yet contains greater number and more diverse set of sequences from fish than SwissProt. In this search, the European eel assembly contigs were used as queries and an e-value cutoff of 0.01. Protein descriptions were retrieved for each blast hit and the results were loaded into a MySQL table which was used for a keyword search to identify genes and sets of genes that are differentially expressed and are involved in neuroendocrine and reproductive processes.

### Quantitative polymerase chain reaction

The expression levels for eight differentially expressed genes were determined by quantitative polymerase chain reaction (qPCR) with the same RNA pools that were used for the microarray. This set was chosen because it includes genes with small and large fold changes (fold changes of 1.63-9.78). It includes GALP, GAD1, CNP, CRFBP, GABRD, NPY, MERP and IRX2. TBP and EEF1A1 were used as controls. For qPCR, three replicates for each condition (immature and immature) were used instead of four because there was not enough material left in the other two samples. cDNA was synthesized from RNA in 20 μl reactions containing 500 ng of DNase-treated RNA, 200 ng of random hexamers (Jena Biosciences, Germany), 100 U of RevertAid (Fermentas, Thermo Fisher Scientific, USA) reverse transcriptase (RT) and 8 U of RiboLockRNase Inhibitor (Fermentas). Reactions were incubated for 10 minutes (min) at 25°C and 60 min at 42°C followed by enzyme inactivation for 10 min at 70°C. Quantifications were carried out using the relative standard curve method and the EvaGreen chemistry in duplicate 15 μl reactions containing: 2 μl of cDNA (diluted 1:4), 300 nM each specific primer (Additional file 5: Table S5) and 1x Sso Fast EvaGreen Supermix (Bio-Rad Laboratories, USA). Reactions were carried out using a Bio-Rad iClycler iQ5 qPCR thermocycler with the following cycling conditions: 30 seconds (s) at 95°C, 45 cycles of 5 s at 95°C, 10 s at 60°C and final melting curves between 65 and 98°C. Target specificity for each qPCR reaction was confirmed by verifying that the amplicon was the expected size by gel electrophoresis and by the presence of a single peak in each melting curve. A cDNA synthesis reaction without reverse transcriptase was used as a negative control to ensure all traces of genomic DNA had been removed and no amplification was observed for all primer pairs. Standard curves relating amplification cycle with initial template quantity were generated using serial dilutions of specific RT-PCR products for each gene (obtained from brain cDNA using the same primers) and included in all quantification plates, with qPCR efficiency ranging between 85–97% with R^2^ > 0.98. The geometric mean of reference genes TBP and EEF1A1 was used to normalize expression data. Relative expression levels were calculated by dividing the expression of each target gene by the geometric mean of the two reference genes.

To verify that the fold changes observed in the microarray experiments correlated with the fold changes observed in the qPCR experiments, Spearman’s rank order correlation was used. Fold changes for each biological replicate were calculated using the mean expression values from the immature brain samples. Student's t-tests were used to determine if expression levels measured by qPCR were significantly different in the brains of immature and mature males. The normality and equal variance assumptions were verified using Shapiro-Wilk’s test and equal variance tests.

## Electronic supplementary material

Additional file 1: Table S1: Functional annotation of differentially expressed genes in the brains of sexually mature male eels. The set of sequence identifiers were obtained from a BLASTX search of SwissProt and Refseq proteins. For functional annotation analysis with DAVID, only BLAST alignments with an e-value less than or equal to 0.001 were considered. To identify differentially expressed genes, a false discovery rate (q-value) of 0.05 was used. The standard default settings that include an EASE score of 0.1 in DAVID were used as well as the following annotation categories: GO terms, Interpro, KEGG pathways and SP PIR keywords. (PDF 668 KB)

Additional file 2: Table S2: Clusters of functionally related genes that are up-regulated in the brains of sexually mature male eels. For the clustering analysis with DAVID, only BLAST alignments with an e-value less than or equal to 0.001 were considered and a false discovery rate (q-value) of 0.05 was used. For the DAVID analysis, the standard default setting was used and only clusters with EASE scores greater than or equal to 1.3 were considered. (PDF 427 KB)

Additional file 3: Table S3: Clusters of functionally related genes that are down-regulated in the brains of sexually mature male eels. For the clustering analysis with DAVID, only BLAST alignments with an e-value less than or equal to 0.001 were considered and a false discovery rate (q-value) of 0.05 was used. For the DAVID analysis, the standard default settings was used and only clusters with EASE scores of greater than or equal to 1.3 were considered. (PDF 475 KB)

Additional file 4: Table S4: Differential gene expression results and BLASTX hits from RefSeq proteins. Complete list of significant differentially expressed genes. The transcriptome assembly contigs upon which the array probes were designed [62] are in the first column. Up-regulated genes (+) are those that were expressed in higher levels in the brains of sexually mature males and down-regulated genes (-) are those with expression levels that were lower than in the brains of immature males. A false discovery rate of 0.05 and a minimum fold change of 1.5 was used. Protein descriptions are based on BLASTX alignments to RefSeq proteins. (PDF 2 MB)

Additional file 5: Table S5: Primer sequences for qPCR. List of primer sequences used for qPCR and the expected product sizes in base pairs (bp). Primers were designed to amplify the following differentially expressed genes: galanin peptide (GALP), glutamate decarboxylase 1 (GAD1), corticotropin releasing factor binding protein (CRFBP), C-type natriuretic peptide (CNP), gamma-aminobutyric acid receptor subunit δ (GABRD), neuropeptide Y (NPY), mammalian ependymin-related protein (MERP) and the iroquois-class homeodomain protein 2 (IRX2). The TATA box binding protein (TBP) and Elongation factor 1-alpha 1 (EEF1A1) were used as controls. (PDF 259 KB)

## Abbreviations

CALM: Calmodulin
CAMK2: Calcium/calmodulin-dependant protein kinase 2
CNP: C-type natriuretic peptide
CRFBP: Corticotropin releasing factor binding protein
EEF1A1: Elongation factor 1-alpha 1
FSH: Follicle-stimulating hormone
GABA: Gamma-aminobutyric acid
GABRD: γ-aminobutyric acid receptor subunit δ
GAD1: Glutamate decarboxylase 1
GALP: Galanin peptide
GH: Growth hormone
GnRH: Gonadotropin releasing-hormone
GPCRs: G protein-coupled receptors
GSI: Gonadosomal index
GTH: Gonadotropin hormone
hCG: Human chorionic gonadotropin
HES4: Transcription factor HES-4
IRX2: Iroquois-class homeodomain protein 2
IRX5: Iroquois-class homeodomain protein 5
LH: Luteinizing hormone
MERP: Mammalian ependymin-related protein
NPY: Neuropeptide Y
PGF_2α_: Prostaglandin-F_2α_
qPCR: Quantitative polymerase chain reaction
TBP: TATA binding protein.
