# Supplementary material for: Changes in the gene expression profiles of the brains of male European eels (Anguilla anguilla) during sexual maturation
Source: BMC Genomics. 2014 Sep 17;15(1):799. doi: 10.1186/1471-2164-15-799 (PMC4175612; doi:10.1186/1471-2164-15-799)
Supplement: Supplementary file 1 — Additional file 1: Table S1: Functional annotation of differentially expressed genes in the brains of sexually mature male eels. The set of sequence identifiers were obtained from a BLASTX search of SwissProt and Refseq proteins. For functional annotation analysis with DAVID, only BLAST alignments with an e-value less than or equal to 0.001 were considered. To identify differentially expressed genes, a false discovery rate (q-value) of 0.05 was used. The standard default settings that include an EASE score of 0.1 in DAVID were used as well as the following annotation categories: GO terms, Interpro, KEGG pathways and SP PIR keywords. (PDF 668 KB) [file 12864_2014_6477_MOESM1_ESM.pdf]

Additional file 1: Table S1. Functional annotation of differentially expressed genes in the brains of sexually mature male eels.

up/ down	Category	Term	Count	List total	Pop hits	Fold enrich	P-value
+	SP_PIR_KEYWORDS	calcium binding	16	498	56	3.17	0.000
+	INTERPRO	IPR018247:EF-HAND 1	23	469	106	2.45	0.000
+	SP_PIR_KEYWORDS	EF hand	13	498	40	3.61	0.000
+	SP_PIR_KEYWORDS	Homeobox	15	498	52	3.2	0.000
+	SP_PIR_KEYWORDS	extracellular matrix	17	498	72	2.62	0.001
+	INTERPRO	IPR017970:Homeobox; conserved site	13	469	48	3.06	0.001
+	INTERPRO	IPR001356:Homeobox	13	469	49	3	0.001
+	GOTERM_CC_FAT	GO:0031012~extracellular matrix	19	352	92	2.21	0.002
+	SP_PIR_KEYWORDS	neuropeptide	6	498	11	6.05	0.002
+	INTERPRO	IPR012287:Homeodomain-related	12	469	47	2.88	0.002
+	INTERPRO	IPR018248:EF hand	16	469	76	2.38	0.002
+	INTERPRO	IPR003893:Iroquois-class homeodomain protein	4	469	4	11.3	0.003
+	INTERPRO	IPR018249:EF-HAND 2	20	469	108	2.09	0.003
+	GOTERM_CC_FAT	GO:0005578~proteinaceous extracellular matrix	18	352	89	2.17	0.003
+	INTERPRO	IPR008160:Collagen triple helix repeat	9	469	30	3.39	0.004
+	SP_PIR_KEYWORDS	blocked amino end	10	498	36	3.08	0.004
+	GOTERM_CC_FAT	GO:0044420~extracellular matrix part	10	352	35	3.06	0.004
+	GOTERM_MF_FAT	GO:0005509~calcium ion binding	42	357	309	1.53	0.005
+	SP_PIR_KEYWORDS	developmental protein	35	498	240	1.62	0.005
+	SP_PIR_KEYWORDS	neurogenesis	13	498	59	2.45	0.005
+	SP_PIR_KEYWORDS	hydroxylysine	5	498	9	6.17	0.006
+	GOTERM_MF_FAT	GO:0048154~S100 beta binding	4	357	5	9.02	0.006
+	GOTERM_BP_FAT	GO:0043583~ear development	8	355	27	3.42	0.007
+	SP_PIR_KEYWORDS	hydroxylation	8	498	26	3.42	0.007
+	GOTERM_MF_FAT	GO:0004866~endopeptidase inhibitor activity	10	357	40	2.82	0.007
+	GOTERM_MF_FAT	GO:0030414~peptidase inhibitor activity	10	357	40	2.82	0.007
+	GOTERM_MF_FAT	GO:0004857~enzyme inhibitor activity	15	357	78	2.17	0.008
+	SP_PIR_KEYWORDS	hydroxyproline	5	498	10	5.55	0.009

+	SP_PIR_KEYWORDS	microsome	10	498	41	2.71	0.009
+	GOTERM_BP_FAT	GO:0002009~morphogenesis of an epithelium	8	355	29	3.18	0.010
+	GOTERM_BP_FAT	GO:0001656~metanephros development	4	355	6	7.69	0.011
+	GOTERM_BP_FAT	GO:0045665~negative regulation of neuron differentiation	5	355	11	5.25	0.011
+	GOTERM_BP_FAT	GO:0050767~regulation of neurogenesis	9	355	37	2.81	0.012
+	SP_PIR_KEYWORDS	calcium	37	498	275	1.49	0.013
+	GOTERM_BP_FAT	GO:0007218~neuropeptide signaling pathway	7	355	24	3.37	0.014
+	GOTERM_BP_FAT	GO:0034637~cellular carbohydrate biosynthetic process	7	355	24	3.37	0.014
+	SP_PIR_KEYWORDS	collagen	8	498	30	2.96	0.015
+	SP_PIR_KEYWORDS	Secreted	44	498	345	1.42	0.016
+	GOTERM_BP_FAT	GO:0051960~regulation of nervous system development	9	355	39	2.66	0.016
+	GOTERM_BP_FAT	GO:0045664~regulation of neuron differentiation	8	355	32	2.89	0.017
+	GOTERM_BP_FAT	GO:0045666~positive regulation of neuron differentiation	4	355	7	6.59	0.017
+	GOTERM_MF_FAT	GO:0016645~oxidoreductase activity; acting on the CH-NH group of donors	4	357	7	6.44	0.018
+	GOTERM_MF_FAT	GO:0016646~oxidoreductase activity; acting on the CH-NH group of donors; NAD or NADP as acceptor	4	357	7	6.44	0.018
+	GOTERM_MF_FAT	GO:0005391~sodium:potassium-exchanging ATPase activity	4	357	7	6.44	0.018
+	INTERPRO	IPR002048:Calcium-binding EF-hand	15	469	87	1.95	0.019
+	KEGG_PATHWAY	hsa04115:p53 signaling pathway	4	157	7	6.24	0.020
+	GOTERM_BP_FAT	GO:0045026~plasma membrane fusion	3	355	3	11.54	0.021
+	GOTERM_BP_FAT	GO:0007342~fusion of sperm to egg plasma membrane	3	355	3	11.54	0.021
+	GOTERM_BP_FAT	GO:0030913~paranodal junction assembly	3	355	3	11.54	0.021
+	INTERPRO	IPR004361:Glyoxalase I	3	469	3	11.3	0.022
+	INTERPRO	IPR018146:Glyoxalase I; conserved site	3	469	3	11.3	0.022

+	GOTERM_MF_FAT	GO:0008934~inositol-1(or 4)-monophosphatase activity	3	357	3	11.27	0.022
+	GOTERM_MF_FAT	GO:0004462~lactoylglutathione lyase activity	3	357	3	11.27	0.022
+	INTERPRO	IPR001751:S100/CaBP-9k-type; calcium binding	5	469	13	4.34	0.023
+	INTERPRO	IPR013787:S100/CaBP-9k-type; calcium binding; subdomain	5	469	13	4.34	0.023
+	INTERPRO	IPR016040:NAD(P)-binding domain	13	469	72	2.04	0.023
+	GOTERM_BP_FAT	GO:0007423~sensory organ development	14	355	83	1.95	0.024
+	GOTERM_BP_FAT	GO:0060284~regulation of cell development	9	355	42	2.47	0.025
+	INTERPRO	IPR011992:EF-Hand type	18	469	116	1.75	0.025
+	SP_PIR_KEYWORDS	palmitate	9	498	41	2.44	0.027
+	SP_PIR_KEYWORDS	glycolysis	6	498	20	3.33	0.029
+	GOTERM_BP_FAT	GO:0019748~secondary metabolic process	6	355	21	3.3	0.030
+	SP_PIR_KEYWORDS	lipoprotein	25	498	181	1.53	0.033
+	INTERPRO	IPR001664:Intermediate filament protein	5	469	15	3.77	0.037
+	INTERPRO	IPR018039:Intermediate filament protein; conserved site	5	469	15	3.77	0.037
+	INTERPRO	IPR016044:Filament	5	469	15	3.77	0.037
+	INTERPRO	IPR006688:ADP-ribosylation factor	5	469	15	3.77	0.037
+	INTERPRO	IPR000594:UBA/THIF-type NAD/FAD binding fold	4	469	9	5.02	0.038
+	INTERPRO	IPR018503:Tetraspanin; conserved site	6	469	22	3.08	0.039
+	GOTERM_BP_FAT	GO:0048598~embryonic morphogenesis	12	355	71	1.95	0.039
+	SP_PIR_KEYWORDS	Intermediate filament	5	498	15	3.7	0.040
+	GOTERM_BP_FAT	GO:0032288~myelin assembly	3	355	4	8.66	0.040
+	GOTERM_BP_FAT	GO:0016051~carbohydrate biosynthetic process	7	355	30	2.69	0.040
+	INTERPRO	IPR000760:Inositol monophosphatase	3	469	4	8.47	0.041
+	GOTERM_BP_FAT	GO:0042471~ear morphogenesis	6	355	23	3.01	0.043
+	GOTERM_CC_FAT	GO:0044421~extracellular region part	30	352	226	1.42	0.044
+	GOTERM_BP_FAT	GO:0044057~regulation of system process	11	355	64	1.98	0.046

+	GOTERM_BP_FAT	GO:0030198~extracellular matrix organization	7	355	31	2.61	0.046
+	SP_PIR_KEYWORDS	triple helix	4	498	10	4.44	0.054
+	INTERPRO	IPR000301:Tetraspanin; subgroup	6	469	24	2.82	0.055
+	SP_PIR_KEYWORDS	er-golgi transport	9	498	47	2.13	0.056
+	SP_PIR_KEYWORDS	lipid-binding	9	498	47	2.13	0.056
+	SP_PIR_KEYWORDS	nadp	10	498	56	1.98	0.060
+	INTERPRO	IPR000533:Tropomyosin	3	469	5	6.78	0.065
+	SP_PIR_KEYWORDS	lithium	3	498	5	6.66	0.067
+	KEGG_PATHWAY	dre00010:Glycolysis / Gluconeogenesis	3	157	5	6.55	0.068
+	INTERPRO	IPR018499:Tetraspanin	6	469	26	2.61	0.074
+	INTERPRO	IPR012335:Thioredoxin fold	9	469	51	1.99	0.077
+	INTERPRO	IPR000073:Alpha/beta hydrolase fold-1	4	469	12	3.77	0.083
+	SP_PIR_KEYWORDS	endoplasmic reticulum	31	498	260	1.32	0.090
+	INTERPRO	IPR002379:ATPase; F0/V0 complex; subunit C	3	469	6	5.65	0.092
+	INTERPRO	IPR000886:Endoplasmic reticulum; targeting sequence	5	469	20	2.82	0.094
+	INTERPRO	IPR006689:ARF/SAR superfamily	5	469	20	2.82	0.094
+	SP_PIR_KEYWORDS	sodium/potassium transport	3	498	6	5.55	0.095
-	GOTERM_BP_FAT	GO:0019882~antigen processing and presentation	7	163	23	7.65	0.000
-	GOTERM_BP_FAT	GO:0042127~regulation of cell proliferation	17	163	175	2.44	0.001
-	GOTERM_CC_FAT	GO:0042611~MHC protein complex	5	142	14	9.48	0.001
-	GOTERM_BP_FAT	GO:0048002~antigen processing and presentation of peptide antigen	5	163	14	8.98	0.002
-	SP_PIR_KEYWORDS	alternative splicing	64	227	1104	1.41	0.002
-	SP_PIR_KEYWORDS	mrna splicing	12	227	103	2.84	0.003
-	GOTERM_BP_FAT	GO:0006397~mRNA processing	14	163	140	2.51	0.003
-	GOTERM_BP_FAT	GO:0007243~protein kinase cascade	10	163	78	3.22	0.003
-	GOTERM_BP_FAT	GO:0008380~RNA splicing	13	163	126	2.59	0.004
-	SP_PIR_KEYWORDS	mrna processing	13	227	122	2.59	0.004
-	INTERPRO	IPR003597:Immunoglobulin C1-set	5	214	17	7.28	0.004
-	INTERPRO	IPR006574:SPRY-associated	6	214	28	5.31	0.005
-	GOTERM_BP_FAT	GO:0006955~immune response	14	163	146	2.41	0.005
-	GOTERM_CC_FAT	GO:0005886~plasma membrane	44	142	795	1.47	0.005
-	INTERPRO	IPR003879:Butyrophylin-like	6	214	29	5.12	0.005

-	INTERPRO	IPR003006:Immunoglobulin/major histocompatibility complex; conserved site	5	214	19	6.52	0.006
-	SP_PIR_KEYWORDS	nucleotide-binding	41	227	662	1.51	0.007
-	INTERPRO	IPR011993:Pleckstrin homology-type	10	214	85	2.91	0.007
-	GOTERM_BP_FAT	GO:0016071~mRNA metabolic process	14	163	154	2.28	0.007
-	GOTERM_BP_FAT	GO:0050858~negative regulation of antigen receptor-mediated signaling pathway	3	163	4	18.85	0.009
-	GOTERM_BP_FAT	GO:0050860~negative regulation of T cell receptor signaling pathway	3	163	4	18.85	0.009
-	SP_PIR_KEYWORDS	immune response	8	227	58	3.36	0.009
-	SP_PIR_KEYWORDS	coiled coil	36	227	576	1.52	0.010
-	GOTERM_BP_FAT	GO:0002474~antigen processing and presentation of peptide antigen via MHC class I	4	163	12	8.38	0.010
-	GOTERM_MF_FAT	GO:0000166~nucleotide binding	52	177	866	1.37	0.011
-	INTERPRO	IPR001849:Pleckstrin homology	9	214	76	2.93	0.011
-	INTERPRO	IPR001870:B302 (SPRY)-like	6	214	35	4.24	0.012
-	INTERPRO	IPR003877:SPla/RYanodine receptor SPRY	6	214	35	4.24	0.012
-	GOTERM_BP_FAT	GO:0000375~RNA splicing; via transesterification reactions	7	163	50	3.52	0.013
-	GOTERM_BP_FAT	GO:0000398~nuclear mRNA splicing; via spliceosome	7	163	50	3.52	0.013
-	GOTERM_BP_FAT	GO:0000377~RNA splicing; via transesterification reactions with bulged adenosine as nucleophile	7	163	50	3.52	0.013
-	SP_PIR_KEYWORDS	serine/threonine-protein kinase	11	227	109	2.46	0.013
-	INTERPRO	IPR013783:Immunoglobulin-like fold	11	214	113	2.41	0.015
-	SP_PIR_KEYWORDS	atp-binding	31	227	492	1.53	0.016
-	INTERPRO	IPR002290:Serine/threonine protein kinase	9	214	83	2.68	0.018
-	GOTERM_BP_FAT	GO:0007411~axon guidance	4	163	15	6.7	0.020
-	GOTERM_MF_FAT	GO:0032553~ribonucleotide binding	43	177	710	1.38	0.020
-	GOTERM_MF_FAT	GO:0032555~purine ribonucleotide binding	43	177	710	1.38	0.020

-	GOTERM_BP_FAT	GO:0050856~regulation of T cell receptor signaling pathway	3	163	6	12.57	0.021
-	GOTERM_CC_FAT	GO:0030054~cell junction	12	142	148	2.15	0.021
-	GOTERM_CC_FAT	GO:0045202~synapse	9	142	95	2.52	0.025
-	GOTERM_CC_FAT	GO:0030666~endocytic vesicle membrane	3	142	7	11.38	0.026
-	GOTERM_BP_FAT	GO:0006928~cell motion	9	163	91	2.49	0.026
-	GOTERM_BP_FAT	GO:0051329~interphase of mitotic cell cycle	4	163	17	5.91	0.027
-	GOTERM_BP_FAT	GO:0051325~interphase	4	163	17	5.91	0.027
-	GOTERM_BP_FAT	GO:0050854~regulation of antigen receptor-mediated signaling pathway	3	163	7	10.77	0.029
-	INTERPRO	IPR007111:NACHT nucleoside triphosphatase	3	214	7	10.61	0.030
-	GOTERM_BP_FAT	GO:0006468~protein amino acid phosphorylation	14	163	185	1.9	0.030
-	SP_PIR_KEYWORDS	mhc ii	3	227	7	10.44	0.031
-	INTERPRO	IPR000315:Zinc finger; B-box	5	214	30	4.13	0.031
-	SP_PIR_KEYWORDS	phosphoprotein	95	227	1963	1.18	0.031
-	SP_PIR_KEYWORDS	cell junction	11	227	125	2.14	0.031
-	GOTERM_MF_FAT	GO:0017076~purine nucleotide binding	43	177	731	1.34	0.031
-	GOTERM_CC_FAT	GO:0044459~plasma membrane part	27	142	485	1.48	0.034
-	GOTERM_BP_FAT	GO:0007626~locomotory behavior	7	163	63	2.79	0.037
-	GOTERM_BP_FAT	GO:0000082~G1/S transition of mitotic cell cycle	3	163	8	9.43	0.037
-	INTERPRO	IPR011161:MHC class I-like antigen recognition	3	214	8	9.28	0.038
-	INTERPRO	IPR006703:AIG1	3	214	8	9.28	0.038
-	INTERPRO	IPR001039:MHC class I; alpha chain; alpha1 and alpha2	3	214	8	9.28	0.038
-	INTERPRO	IPR000387:Protein-tyrosine phosphatase	4	214	19	5.21	0.039
-	GOTERM_BP_FAT	GO:0044057~regulation of system process	7	163	64	2.75	0.039
-	SP_PIR_KEYWORDS	mhc i	3	227	8	9.13	0.040
-	SP_PIR_KEYWORDS	leucine-rich repeat	8	227	79	2.47	0.042
-	GOTERM_CC_FAT	GO:0042612~MHC class I protein complex	3	142	9	8.85	0.042
-	GOTERM_BP_FAT	GO:0000910~cytokinesis	4	163	20	5.03	0.042

-	INTERPRO	IPR008271:Serine/threonine protein kinase; active site	9	214	98	2.27	0.043
-	INTERPRO	IPR000387:Dual-specific/protein-tyrosine phosphatase; conserved region	4	214	20	4.95	0.044
-	GOTERM_MF_FAT	GO:0005524~ATP binding	33	177	543	1.38	0.044
-	GOTERM_BP_FAT	GO:0010648~negative regulation of cell communication	7	163	66	2.67	0.045
-	INTERPRO	IPR012677:Nucleotide-binding; alpha-beta plait	8	214	82	2.42	0.046
-	INTERPRO	IPR000504:RNA recognition motif; RNP-1	8	214	82	2.42	0.046
-	GOTERM_CC_FAT	GO:0044456~synapse part	6	142	53	3.01	0.046
-	GOTERM_BP_FAT	GO:0045927~positive regulation of growth	4	163	21	4.79	0.048
-	SP_PIR_KEYWORDS	polymorphism	49	227	936	1.27	0.050
-	INTERPRO	IPR017442:Serine/threonine protein kinase-related	9	214	102	2.18	0.052
-	SP_PIR_KEYWORDS	kinase	15	227	216	1.69	0.058
-	INTERPRO	IPR000330:SNF2-related	3	214	11	6.75	0.070
-	INTERPRO	IPR018355:SPla/RYanodine receptor subgroup	4	214	25	3.96	0.077
-	INTERPRO	IPR008803:Root hair defective 3 GTP-binding	2	214	2	24.76	0.079
-	INTERPRO	IPR007275:YT521-B-like protein	2	214	2	24.76	0.079
-	INTERPRO	IPR003656:Zinc finger; BED-type predicted	2	214	2	24.76	0.079
-	INTERPRO	IPR000008:C2 calcium-dependent membrane targeting	5	214	41	3.02	0.081
-	KEGG_PATHWAY	hsa04010:MAPK signaling pathway	4	72	25	3.81	0.082
-	SP_PIR_KEYWORDS	tyrosine-specific phosphatase	3	227	12	6.09	0.084
-	INTERPRO	IPR016130:Protein-tyrosine phosphatase; active site	4	214	26	3.81	0.085
-	SP_PIR_KEYWORDS	cytoplasm	64	227	1324	1.18	0.093
-	SP_PIR_KEYWORDS	nucleus	63	227	1304	1.18	0.097
-	INTERPRO	IPR017441:Protein kinase; ATP binding site	9	214	117	1.9	0.098
-	SP_PIR_KEYWORDS	dna-binding	23	227	403	1.39	0.098
-	KEGG_PATHWAY	hsa04144:Endocytosis	3	72	13	5.5	0.098
