# Supplementary material for: Changes in the gene expression profiles of the brains of male European eels (Anguilla anguilla) during sexual maturation
Source: BMC Genomics. 2014 Sep 17;15(1):799. doi: 10.1186/1471-2164-15-799 (PMC4175612; doi:10.1186/1471-2164-15-799)
Supplement: Supplementary file 2 — Additional file 2: Table S2: Clusters of functionally related genes that are up-regulated in the brains of sexually mature male eels. For the clustering analysis with DAVID, only BLAST alignments with an e-value less than or equal to 0.001 were considered and a false discovery rate (q-value) of 0.05 was used. For the DAVID analysis, the standard default setting was used and only clusters with EASE scores greater than or equal to 1.3 were considered. (PDF 427 KB) [file 12864_2014_6477_MOESM2_ESM.pdf]

Additional file 2: Table S2. Clusters of functionally related genes that are up-regulated in the brains of sexually mature male eels

**Annotation Cluster 1**	**Enrichment Score: 2.817136811147704**										
Category	Term	Count	%	PValue	List Total	Pop Hits	Pop Total	Fold Enrichment	Bonferroni	Benjamini	FDR
UP_SEQ_FEATURE	DNA-binding region:Homeobox; TALE-type	5	0.942	0.001	495	6	5523	9.298	0.740	0.740	1.368
SMART	SM00548:IRO	4	0.753	0.002	198	4	2565	12.955	0.229	0.122	2.003
INTERPRO	IPR003893:Iroquois-class homeodomain protein	4	0.753	0.003	469	4	5298	11.296	0.884	0.302	3.884
**Annotation Cluster 2**	**Enrichment Score: 2.2791263907808483**										
Category	Term	Count	%	PValue	List Total	Pop Hits	Pop Total	Fold Enrichment	Bonferroni	Benjamini	FDR
SP_PIR_KEYWORDS	calcium binding	16	3.013	0.000	498	56	5528	3.172	0.034	0.034	0.116
INTERPRO	IPR018247:EF-HAND 1	23	4.331	0.000	469	106	5298	2.451	0.087	0.087	0.166
SP_PIR_KEYWORDS	EF hand	13	2.448	0.000	498	40	5528	3.608	0.053	0.027	0.183
INTERPRO	IPR018248:EF hand	16	3.013	0.002	469	76	5298	2.378	0.850	0.316	3.424
UP_SEQ_FEATURE	domain:EF-hand 1	20	3.766	0.002	495	106	5523	2.105	0.980	0.858	3.923
INTERPRO	IPR018249:EF-HAND 2	20	3.766	0.003	469	108	5298	2.092	0.885	0.265	3.889
UP_SEQ_FEATURE	domain:EF-hand 2	20	3.766	0.003	495	108	5523	2.066	0.992	0.803	4.868
GOTERM_MF_FAT	GO:0005509~calcium ion binding	42	7.910	0.004	357	309	4024	1.532	0.894	0.894	6.282
SMART	SM00054:EFh	15	2.825	0.006	198	87	2565	2.234	0.576	0.249	6.466
UP_SEQ_FEATURE	calcium-binding region:3	7	1.318	0.006	495	20	5523	3.905	1.000	0.882	10.346
UP_SEQ_FEATURE	calcium-binding region:2	13	2.448	0.013	495	66	5523	2.198	1.000	0.906	19.555
SP_PIR_KEYWORDS	calcium	37	6.968	0.013	498	275	5528	1.494	0.996	0.340	16.762
UP_SEQ_FEATURE	calcium-binding region:1	14	2.637	0.013	495	74	5523	2.111	1.000	0.884	19.804
INTERPRO	IPR002048:Calcium-binding EF-hand	15	2.825	0.019	469	87	5298	1.948	1.000	0.837	25.937
INTERPRO	IPR011992:EF-Hand type	18	3.390	0.025	469	116	5298	1.753	1.000	0.806	32.433
UP_SEQ_FEATURE	domain:EF-hand 3	11	2.072	0.051	495	63	5523	1.948	1.000	0.995	58.702
UP_SEQ_FEATURE	calcium-binding region:4	3	0.565	0.124	495	7	5523	4.782	1.000	1.000	89.193
UP_SEQ_FEATURE	domain:EF-hand 4	6	1.130	0.183	495	34	5523	1.969	1.000	1.000	96.657
**Annotation Cluster 3**	**Enrichment Score: 2.1676404071572217**										
Category	Term	Count	%	PValue	List Total	Pop Hits	Pop Total	Fold Enrichment	Bonferroni	Benjamini	FDR
SP_PIR_KEYWORDS	extracellular matrix	17	3.202	0.001	498	72	5528	2.621	0.190	0.051	0.711
GOTERM_CC_FAT	GO:0031012~extracellular matrix	19	3.578	0.002	352	92	3770	2.212	0.425	0.425	2.233

GOTERM_CC_FAT	GO:0005578~proteinaceous extracellular matrix	18	3.390	0.003	352	89	3770	2.166	0.619	0.383	3.871
SP_PIR_KEYWORDS	hydroxylation	8	1.507	0.007	498	26	5528	3.416	0.935	0.239	8.849
SP_PIR_KEYWORDS	Secreted	44	8.286	0.016	498	345	5528	1.416	0.999	0.358	20.171
GOTERM_CC_FAT	GO:0044421~extracellular region part	30	5.650	0.044	352	226	3770	1.422	1.000	0.975	45.319
GOTERM_CC_FAT	GO:0005576~extracellular region	50	9.416	0.060	352	425	3770	1.260	1.000	0.983	56.607
**Annotation Cluster 4**	**Enrichment Score: 1.7865065642320699**										
Category	Term	Count	%	PValue	List Total	Pop Hits	Pop Total	Fold Enrichment	Bonferroni	Benjamini	FDR
SP_PIR_KEYWORDS	developmental protein	35	6.591	0.005	498	240	5528	1.619	0.855	0.241	6.349
SP_PIR_KEYWORDS	neurogenesis	13	2.448	0.005	498	59	5528	2.446	0.894	0.244	7.318
SP_PIR_KEYWORDS	differentiation	16	3.013	0.173	498	128	5528	1.388	1.000	0.859	93.019
**Annotation Cluster 5**	**Enrichment Score: 1.452163909019713**										
Category	Term	Count	%	PValue	List Total	Pop Hits	Pop Total	Fold Enrichment	Bonferroni	Benjamini	FDR
UP_SEQ_FEATURE	DNA-binding region:Homeobox; TALE-type	5	0.942	0.001	495	6	5523	9.298	0.740	0.740	1.368
GOTERM_BP_FAT	GO:0045665~negative regulation of neuron differentiation	5	0.942	0.011	355	11	4097	5.246	1.000	0.986	16.935
GOTERM_BP_FAT	GO:0050767~regulation of neurogenesis	9	1.695	0.012	355	37	4097	2.807	1.000	0.975	18.095
GOTERM_BP_FAT	GO:0051960~regulation of nervous system development	9	1.695	0.016	355	39	4097	2.663	1.000	0.973	23.966
GOTERM_BP_FAT	GO:0045664~regulation of neuron differentiation	8	1.507	0.017	355	32	4097	2.885	1.000	0.964	25.038
GOTERM_BP_FAT	GO:0045666~positive regulation of neuron differentiation	4	0.753	0.017	355	7	4097	6.595	1.000	0.948	25.104
UP_SEQ_FEATURE	chain:Iroquois-class homeodomain protein irx-3	3	0.565	0.023	495	3	5523	11.158	1.000	0.967	31.805
GOTERM_BP_FAT	GO:0060284~regulation of cell development	9	1.695	0.025	355	42	4097	2.473	1.000	0.960	34.361
GOTERM_BP_FAT	GO:0009954~proximal/distal pattern formation	3	0.565	0.147	355	8	4097	4.328	1.000	0.988	92.970

GOTERM_BP_FAT	GO:0045596~negative regulation of cell differentiation	8	1.507	0.232	355	58	4097	1.592	1.000	0.993	98.756
GOTERM_BP_FAT	GO:0051094~positive regulation of developmental process	7	1.318	0.527	355	67	4097	1.206	1.000	0.999	100.000
GOTERM_BP_FAT	GO:0045597~positive regulation of cell differentiation	5	0.942	0.712	355	55	4097	1.049	1.000	1.000	100.000
**Annotation Cluster 6**	**Enrichment Score: 1.4360318194371593**										
Category	Term	Count	%	PValue	List Total	Pop Hits	Pop Total	Fold Enrichment	Bonferroni	Benjamini	FDR
GOTERM_CC_FAT	GO:0044420~extracellular matrix part	10	1.883	0.004	352	35	3770	3.060	0.713	0.340	4.974
SP_PIR_KEYWORDS	basement membrane	4	0.753	0.105	498	13	5528	3.416	1.000	0.816	78.831
GOTERM_CC_FAT	GO:0005604~basement membrane	5	0.942	0.125	352	21	3770	2.550	1.000	0.975	83.659
**Annotation Cluster 7**	**Enrichment Score: 1.408056635907251**										
Category	Term	Count	%	PValue	List Total	Pop Hits	Pop Total	Fold Enrichment	Bonferroni	Benjamini	FDR
SP_PIR_KEYWORDS	neuropeptide	6	1.130	0.002	498	11	5528	6.055	0.501	0.130	2.331
GOTERM_BP_FAT	GO:0007218~neuropeptide signaling pathway	7	1.318	0.014	355	24	4097	3.366	1.000	0.973	20.999
SP_PIR_KEYWORDS	cleavage on pair of basic residues	8	1.507	0.235	498	56	5528	1.586	1.000	0.905	97.663
GOTERM_BP_FAT	GO:0007186~G-protein coupled receptor protein signaling pathway	12	2.260	0.420	355	116	4097	1.194	1.000	0.997	99.988
**Annotation Cluster 8**	**Enrichment Score: 1.3402763897531442**										
Category	Term	Count	%	PValue	List Total	Pop Hits	Pop Total	Fold Enrichment	Bonferroni	Benjamini	FDR
GOTERM_MF_FAT	GO:0048154~S100 beta binding	4	0.753	0.006	357	5	4024	9.017	0.950	0.776	8.294
PIR_SUPERFAMILY	PIRSF002353:S-100 protein	5	0.942	0.017	241	12	2710	4.685	0.978	0.978	19.463
INTERPRO	IPR013787:S100/CaBP-9k-type, calcium binding, subdomain	5	0.942	0.023	469	13	5298	4.345	1.000	0.825	29.708
INTERPRO	IPR001751:S100/CaBP-9k-type, calcium binding	5	0.942	0.023	469	13	5298	4.345	1.000	0.825	29.708

UP_SEQ_FEATURE	calcium-binding region:1; low affinity	4	0.753	0.053	495	10	5523	4.463	1.000	0.995	59.965
UP_SEQ_FEATURE	calcium-binding region:2; high affinity	4	0.753	0.053	495	10	5523	4.463	1.000	0.995	59.965
GOTERM_MF_FAT	GO:0042803~protein homodimerization activity	10	1.883	0.362	357	87	4024	1.296	1.000	0.995	99.846
SP_PIR_KEYWORDS	brain	3	0.565	0.363	498	14	5528	2.379	1.000	0.948	99.819
