# Supplementary material for: Changes in the gene expression profiles of the brains of male European eels (Anguilla anguilla) during sexual maturation
Source: BMC Genomics. 2014 Sep 17;15(1):799. doi: 10.1186/1471-2164-15-799 (PMC4175612; doi:10.1186/1471-2164-15-799)
Supplement: Supplementary file 3 — Additional file 3: Table S3: Clusters of functionally related genes that are down-regulated in the brains of sexually mature male eels. For the clustering analysis with DAVID, only BLAST alignments with an e-value less than or equal to 0.001 were considered and a false discovery rate (q-value) of 0.05 was used. For the DAVID analysis, the standard default settings was used and only clusters with EASE scores of greater than or equal to 1.3 were considered. (PDF 475 KB) [file 12864_2014_6477_MOESM3_ESM.pdf]

Additional file 3: Table S3. Clusters of functionally related genes that are down-regulated in the brains of sexually mature male eels.

Annotation Cluster 1	Enrichment Score: 2.1286729869075547										
Category	Term	Count	%	PValue	List Total	Pop Hits	Pop Total	Fold Enrichment	Bonferroni	Benjamini	FDR
GOTERM_BP_FAT	GO:0019882~antigen processing and presentation	7	2.905	0.000	163	23	4097	7.650	0.219	0.219	0.325
UP_SEQ_FEATURE	region of interest:Alpha-2	5	2.075	0.000	221	9	5523	13.884	0.174	0.174	0.399
UP_SEQ_FEATURE	region of interest:Alpha-1	5	2.075	0.000	221	9	5523	13.884	0.174	0.174	0.399
GOTERM_CC_FAT	GO:0042611~MHC protein complex	5	2.075	0.001	142	14	3770	9.482	0.284	0.284	1.791
GOTERM_BP_FAT	GO:0048002~antigen processing and presentation of peptide antigen	5	2.075	0.002	163	14	4097	8.977	0.881	0.509	2.767
UP_SEQ_FEATURE	domain:Ig-like C1-type	5	2.075	0.002	221	14	5523	8.925	0.726	0.351	2.676
INTERPRO	IPR003597:Immunoglobulin C1-set	5	2.075	0.004	214	17	5298	7.281	0.837	0.837	5.530
GOTERM_BP_FAT	GO:0006955~immune response	14	5.809	0.005	163	146	4097	2.410	0.997	0.560	7.286
UP_SEQ_FEATURE	region of interest:Connecting peptide	5	2.075	0.005	221	18	5523	6.942	0.970	0.442	7.064
INTERPRO	IPR003006:Immunoglobulin/major histocompatibility complex, conserved site	5	2.075	0.006	214	19	5298	6.515	0.938	0.500	8.340
SP_PIR_KEYWORDS	immune response	8	3.320	0.009	227	58	5528	3.359	0.912	0.385	11.048
SMART	SM00407:IGc1	5	2.075	0.010	135	17	2565	5.588	0.687	0.687	10.790
GOTERM_BP_FAT	GO:0002474~antigen processing and presentation of peptide antigen via MHC class I	4	1.660	0.010	163	12	4097	8.378	1.000	0.720	15.404
INTERPRO	IPR013783:Immunoglobulin-like fold	11	4.564	0.015	214	113	5298	2.410	0.999	0.572	19.210
UP_SEQ_FEATURE	region of interest:Alpha-3	3	1.245	0.029	221	7	5523	10.710	1.000	0.806	36.028
SP_PIR_KEYWORDS	mhc ii	3	1.245	0.031	227	7	5528	10.437	1.000	0.609	33.504
INTERPRO	IPR001039:MHC class I, alpha chain, alpha1 and alpha2	3	1.245	0.038	214	8	5298	9.284	1.000	0.771	42.630
INTERPRO	IPR011161:MHC class I-like antigen recognition	3	1.245	0.038	214	8	5298	9.284	1.000	0.771	42.630

SP_PIR_KEYWORDS	mhc i	3	1.245	0.040	227	8	5528	9.132	1.000	0.601	41.261
GOTERM_CC_FAT	GO:0042612~MHC class I protein complex	3	1.245	0.042	142	9	3770	8.850	1.000	0.768	42.545
SP_PIR_KEYWORDS	heterodimer	4	1.660	0.122	227	30	5528	3.247	1.000	0.802	82.027
INTERPRO	IPR007110:Immunoglobulin-like	8	3.320	0.124	214	104	5298	1.904	1.000	0.917	84.742
**Annotation Cluster 2**	**Enrichment Score: 1.816568104765957**										
Category	Term	Count	%	PValue	List Total	Pop Hits	Pop Total	Fold Enrichment	Bonferroni	Benjamini	FDR
INTERPRO	IPR011993:Pleckstrin homology-type	10	4.149	0.007	214	85	5298	2.913	0.952	0.456	9.116
INTERPRO	IPR001849:Pleckstrin homology	9	3.734	0.011	214	76	5298	2.932	0.992	0.557	14.217
UP_SEQ_FEATURE	domain:PH	8	3.320	0.018	221	68	5523	2.940	1.000	0.803	23.833
SMART	SM00233:PH	9	3.734	0.042	135	76	2565	2.250	0.993	0.811	38.839
**Annotation Cluster 3**	**Enrichment Score: 1.6253411639066149**										
Category	Term	Count	%	PValue	List Total	Pop Hits	Pop Total	Fold Enrichment	Bonferroni	Benjamini	FDR
UP_SEQ_FEATURE	nucleotide phosphate-binding region:ATP	28	11.618	0.000	221	336	5523	2.083	0.210	0.111	0.492
SP_PIR_KEYWORDS	nucleotide-binding	41	17.012	0.006	227	662	5528	1.508	0.830	0.358	8.185
GOTERM_MF_FAT	GO:0000166~nucleotide binding	52	21.577	0.011	177	866	4024	1.365	0.964	0.964	13.362
SP_PIR_KEYWORDS	atp-binding	31	12.863	0.016	227	492	5528	1.534	0.988	0.427	19.317
GOTERM_MF_FAT	GO:0032555~purine ribonucleotide binding	43	17.842	0.020	177	710	4024	1.377	0.998	0.958	23.794
GOTERM_MF_FAT	GO:0032553~ribonucleotide binding	43	17.842	0.020	177	710	4024	1.377	0.998	0.958	23.794
GOTERM_MF_FAT	GO:0017076~purine nucleotide binding	43	17.842	0.031	177	731	4024	1.337	1.000	0.964	34.863
GOTERM_MF_FAT	GO:0005524~ATP binding	33	13.693	0.044	177	543	4024	1.382	1.000	0.971	45.486
UP_SEQ_FEATURE	binding site:ATP	12	4.979	0.050	221	158	5523	1.898	1.000	0.856	53.780
GOTERM_MF_FAT	GO:0032559~adenyl ribonucleotide binding	33	13.693	0.050	177	549	4024	1.367	1.000	0.960	49.972
SP_PIR_KEYWORDS	kinase	15	6.224	0.058	227	216	5528	1.691	1.000	0.662	54.353
GOTERM_MF_FAT	GO:0030554~adenyl nucleotide binding	33	13.693	0.076	177	570	4024	1.316	1.000	0.984	65.677
GOTERM_MF_FAT	GO:0001883~purine nucleoside binding	33	13.693	0.087	177	577	4024	1.300	1.000	0.983	70.606
GOTERM_MF_FAT	GO:0001882~nucleoside binding	33	13.693	0.092	177	580	4024	1.294	1.000	0.965	72.631

Annotation Cluster 4	Enrichment Score: 1.4616635105090412										
Category	Term	Count	%	PValue	List Total	Pop Hits	Pop Total	Fold Enrichment	Bonferroni	Benjamini	FDR
SP_PIR_KEYWORDS	mrna splicing	12	4.979	0.003	227	103	5528	2.837	0.562	0.338	3.904
GOTERM_BP_FAT	GO:0006397~mRNA processing	14	5.809	0.003	163	140	4097	2.513	0.982	0.632	5.119
GOTERM_BP_FAT	GO:0008380~RNA splicing	13	5.394	0.004	163	126	4097	2.593	0.990	0.539	5.931
SP_PIR_KEYWORDS	mrna processing	13	5.394	0.004	227	122	5528	2.595	0.660	0.302	5.069
GOTERM_BP_FAT	GO:0016071~mRNA metabolic process	14	5.809	0.007	163	154	4097	2.285	1.000	0.676	11.185
GOTERM_BP_FAT	GO:0000375~RNA splicing, via transesterification reactions	7	2.905	0.013	163	50	4097	3.519	1.000	0.772	19.255
GOTERM_BP_FAT	GO:0000377~RNA splicing, via transesterification reactions with bulged adenosine as nucleophile	7	2.905	0.013	163	50	4097	3.519	1.000	0.772	19.255
GOTERM_BP_FAT	GO:0000398~nuclear mRNA splicing, via spliceosome	7	2.905	0.013	163	50	4097	3.519	1.000	0.772	19.255
UP_SEQ_FEATURE	domain:RRM	5	2.075	0.030	221	30	5523	4.165	1.000	0.791	36.751
INTERPRO	IPR000504:RNA recognition motif, RNP-1	8	3.320	0.046	214	82	5298	2.415	1.000	0.734	48.571
INTERPRO	IPR012677:Nucleotide-binding, alpha-beta plait	8	3.320	0.046	214	82	5298	2.415	1.000	0.734	48.571
GOTERM_BP_FAT	GO:0006396~RNA processing	15	6.224	0.071	163	231	4097	1.632	1.000	0.961	69.598
GOTERM_CC_FAT	GO:0016604~nuclear body	5	2.075	0.086	142	45	3770	2.950	1.000	0.907	68.509
SMART	SM00360:RRM	8	3.320	0.133	135	82	2565	1.854	1.000	0.937	80.464
SP_PIR_KEYWORDS	rna-binding	13	5.394	0.273	227	239	5528	1.325	1.000	0.955	98.486
GOTERM_MF_FAT	GO:0003723~RNA binding	15	6.224	0.471	177	306	4024	1.114	1.000	0.993	99.981
UP_SEQ_FEATURE	domain:RRM 2	3	1.245	0.564	221	47	5523	1.595	1.000	1.000	100.000
UP_SEQ_FEATURE	domain:RRM 1	3	1.245	0.564	221	47	5523	1.595	1.000	1.000	100.000
**Annotation Cluster 5**	**Enrichment Score: 1.4255472364232042**										
Category	Term	Count	%	PValue	List Total	Pop Hits	Pop Total	Fold Enrichment	Bonferroni	Benjamini	FDR
GOTERM_CC_FAT	GO:0030054~cell junction	12	4.979	0.021	142	148	3770	2.153	0.994	0.820	24.343
GOTERM_CC_FAT	GO:0045202~synapse	9	3.734	0.025	142	95	3770	2.515	0.997	0.772	27.408
SP_PIR_KEYWORDS	cell junction	11	4.564	0.031	227	125	5528	2.143	1.000	0.545	34.120
GOTERM_CC_FAT	GO:0044459~plasma membrane part	27	11.203	0.034	142	485	3770	1.478	1.000	0.746	35.972
GOTERM_CC_FAT	GO:0044456~synapse part	6	2.490	0.046	142	53	3770	3.006	1.000	0.757	45.814
SP_PIR_KEYWORDS	synapse	6	2.490	0.108	227	62	5528	2.357	1.000	0.774	77.852
