# Supplementary material for: Changes in the gene expression profiles of the brains of male European eels (Anguilla anguilla) during sexual maturation
Source: BMC Genomics. 2014 Sep 17;15(1):799. doi: 10.1186/1471-2164-15-799 (PMC4175612; doi:10.1186/1471-2164-15-799)
Supplement: Supplementary file 4 — Additional file 4: Table S4: Differential gene expression results and BLASTX hits from RefSeq proteins. Complete list of significant differentially expressed genes. The transcriptome assembly contigs upon which the array probes were designed [62] are in the first column. Up-regulated genes (+) are those that were expressed in higher levels in the brains of sexually mature males and down-regulated genes (-) are those with expression levels that were lower than in the brains of immature males. A false discovery rate of 0.05 and a minimum fold change of 1.5 was used. Protein descriptions are based on BLASTX alignments to RefSeq proteins. (PDF 2 MB) [file 12864_2014_6477_MOESM4_ESM.pdf]

Additional file 4: Table S4. Differential gene expression results and BLASTX hits from RefSeq proteins

Contig	Direction	Fold change	FDR	Blast hit GI	Protein description
eeel_c10033	+	1.805	0.024	348523077	PREDICTED: DNA-directed RNA polymerases I II and III subunit RPABC3-like [Oreochromis niloticus]
eeel_c10123	+	1.578	0.039	432923650	PREDICTED: protein BCCIP homolog [Oryzias latipes]
eeel_c1026	+	1.726	0.006		
eeel_c10420	+	1.557	0.030	320461693	vacuolar ATPase assembly integral membrane protein VMA21 [Danio rerio]
eeel_c10506	+	2.366	0.011	50345022	small muscular protein [Danio rerio]
eeel_c1056	+	1.621	0.016	348500160	PREDICTED: signal peptidase complex catalytic subunit SEC11A-like [Oreochromis niloticus]
eeel_c10562	+	1.817	0.012	18859137	nuclease-sensitive element-binding protein 1 isoform 2 [Danio rerio]
eeel_c10565	+	1.708	0.017		
eeel_c10574	+	1.811	0.005	403305452	PREDICTED: eosinophil lysophospholipase-like [Saimiri boliviensis boliviensis]
eeel_c10581	+	1.664	0.026	50540014	ADP-ribosylation factor-like protein 1 [Danio rerio]
eeel_c10585	+	1.623	0.005	55742591	prostaglandin E synthase 3 [Danio rerio]
eeel_c1075	+	2.142	0.036	321267514	dermatopontin [Danio rerio]
eeel_c10914	+	1.756	0.012	148922926	succinate dehydrogenase [ubiquinone] iron-sulfur subunit mitochondrial precursor [Danio rerio]
eeel_c11096	+	2.148	0.024	20373145	claudin b [Danio rerio]
eeel_c11140	+	7.592	0.039	348502997	PREDICTED: rhodopsin-like [Oreochromis niloticus]
eeel_c11155	+	2.127	0.047	291407863	PREDICTED: ubiquitin-conjugating enzyme E2A-like isoform 1 [Oryctolagus cuniculus]
eeel_c1119	+	1.723	0.012		
eeel_c11209	+	1.502	0.012	348526434	PREDICTED: isochorismatase domain-containing protein 2 mitochondrial-like [Oreochromis niloticus]
eeel_c11215	+	1.538	0.030		
eeel_c1126	+	3.194	0.030	395534831	PREDICTED: importin subunit alpha-6 [Sarcophilus harrisii]
eeel_c11270	+	1.840	0.005	345493247	PREDICTED: hypothetical protein LOC100122087 [Nasonia vitripennis]
eeel_c11322	+	3.400	0.017	291190642	suppression of tumorigenicity 5 protein [Salmo salar]
eeel_c11330	+	1.807	0.047	291407865	PREDICTED: ubiquitin-conjugating enzyme E2A-like isoform 2 [Oryctolagus cuniculus]
eeel_c11376	+	4.338	0.012		
eeel_c1143	+	1.953	0.011	432855291	PREDICTED: glutaredoxin-2 mitochondrial-like [Oryzias latipes]
eeel_c1166	+	1.727	0.026	410926467	PREDICTED: transmembrane protein 181-like [Takifugu rubripes]
eeel_c11732	+	1.757	0.007		
eeel_c11740	+	1.568	0.021		
eeel_c11900	+	1.678	0.016	229576820	mitochondrial import inner membrane translocase subunit Tim9 [Danio rerio]
eeel_c11902	+	1.802	0.007	498984719	PREDICTED: triosephosphate isomerase B-like [Maylandia zebra]

eeel_c11979	+	1.783	0.014	32308153	annexin A2a [Danio rerio]
eeel_c12395	+	1.501	0.036	348537895	PREDICTED: flavin reductase-like [Oreochromis niloticus]
eeel_c1245	+	1.556	0.007	47086553	dolichyl-diphosphooligosaccharide--protein glycosyltransferase subunit 2 precursor [Danio rerio]
eeel_c12495	+	3.093	0.006	348503530	PREDICTED: inositol monophosphatase 1-like [Oreochromis niloticus]
eeel_c12510	+	1.511	0.036	395529086	PREDICTED: complement component 1 Q subcomponent-binding protein mitochondrial partial [Sarcophilus harrisii]
eeel_c12520	+	2.973	0.014	499032299	PREDICTED: myelin basic protein-like isoform X1 [Maylandia zebra]
eeel_c12545	+	1.907	0.007	507930739	PREDICTED: protein FAM3D [Condylura cristata]
eeel_c12597	+	1.719	0.014	345322223	PREDICTED: LOW QUALITY PROTEIN: afadin- and alpha-actinin-binding protein-like [Ornithorhynchus anatinus]
eeel_c12613	+	1.571	0.006	499037754	PREDICTED: protein TILB homolog isoform X1 [Maylandia zebra]
eeel_c12621	+	1.574	0.016	348526233	PREDICTED: 39S ribosomal protein L51 mitochondrial-like [Oreochromis niloticus]
eeel_c12626	+	1.550	0.036		
eeel_c12628	+	2.750	0.008	292618745	PREDICTED: transcription factor BTF3 homolog 4-like [Danio rerio]
eeel_c12748	+	2.046	0.006	45360661	MID1 interacting protein 1 [Xenopus (Silurana) tropicalis]
eeel_c12825	+	1.835	0.007		
eeel_c12918	+	1.624	0.047	24119230	proteasome alpha 6a subunit [Danio rerio]
eeel_c12961	+	1.945	0.013	213511846	Four and a half LIM domains protein 1 [Salmo salar]
eeel_c13042	+	3.099	0.006	348504688	PREDICTED: tropomyosin alpha-4 chain-like isoform 2 [Oreochromis niloticus]
eeel_c13054	+	1.599	0.030	224809395	collagen alpha-2(V) chain precursor [Danio rerio]
eeel_c13182	+	1.585	0.008	348534969	PREDICTED: acyl carrier protein mitochondrial-like [Oreochromis niloticus]
eeel_c13264	+	1.541	0.026	291190568	Pre-mRNA-splicing factor SYF1 [Salmo salar]
eeel_c13373	+	2.254	0.026	325652192	cocaine- and amphetamine-regulated transcript ch22 precursor [Oryzias latipes]
eeel_c13418	+	1.770	0.036	410905845	PREDICTED: prenylated Rab acceptor protein 1-like [Takifugu rubripes]
eeel_c13554	+	1.639	0.026	213510968	Thymosin beta-a [Salmo salar]
eeel_c13577	+	1.720	0.014	118344634	DAZAP2-like protein [Takifugu rubripes]
eeel_c13619	+	2.843	0.036	301628660	PREDICTED: plectin-1 partial [Xenopus (Silurana) tropicalis]
eeel_c1363	+	1.537	0.009	348503912	PREDICTED: spectrin alpha chain brain isoform 1 [Oreochromis niloticus]
eeel_c13660	+	1.705	0.006	348546295	PREDICTED: lactose-binding lectin l-2-like partial [Oreochromis niloticus]
eeel_c13736	+	1.509	0.026	348503426	PREDICTED: ubiquitin-like modifier-activating enzyme 5-like [Oreochromis niloticus]
eeel_c13752	+	2.079	0.014	348501120	PREDICTED: V-type proton ATPase 21 kDa proteolipid subunit-like [Oreochromis niloticus]
eeel_c1391	+	2.807	0.006	57526522	inositol monophosphatase 1 [Danio rerio]
eeel_c13976	+	1.988	0.009	410928504	PREDICTED: sodium/potassium-transporting ATPase subunit beta-233-like [Takifugu rubripes]
eeel_c14016	+	1.648	0.011	311893347	small integral membrane protein 19 [Danio rerio]
eeel_c14033	+	1.948	0.017		
eeel_c14091	+	2.096	0.030	318068061	mitochondrial hydroxyacyl-coenzyme a dehydrogenase [Ictalurus punctatus]

eeel_c14113	+	4.399	0.016	317575753	c-type natriuretic peptide 1 precursor [Ictalurus punctatus]
eeel_c14171	+	1.735	0.012	498984080	PREDICTED: uncharacterized protein C1orf198 homolog isoform X1 [Maylandia zebra]
eeel_c14191	+	1.607	0.021	27545193	brain creatine kinase b [Danio rerio]
eeel_c14211	+	1.603	0.005	410897104	PREDICTED: sacsin-like [Takifugu rubripes]
eeel_c143	+	1.748	0.016		
eeel_c14393	+	1.739	0.009	348533588	PREDICTED: glutamate receptor U1-like [Oreochromis niloticus]
eeel_c14398	+	1.566	0.016		
eeel_c14525	+	1.664	0.011	259089303	CKLF-like MARVEL transmembrane domain-containing protein 7 [Oncorhynchus mykiss]
eeel_c14553	+	1.602	0.013	348523405	PREDICTED: caveolin-1-like [Oreochromis niloticus]
eeel_c14597	+	1.769	0.024	259089115	TRM112-like protein [Oncorhynchus mykiss]
eeel_c14683	+	1.768	0.009	348500512	PREDICTED: 40S ribosomal protein S28-like isoform 1 [Oreochromis niloticus]
eeel_c14683	+	1.575	0.036	348500512	PREDICTED: 40S ribosomal protein S28-like isoform 1 [Oreochromis niloticus]
eeel_c14690	+	1.565	0.021	348537590	PREDICTED: dynein light chain roadblock-type 1-like isoform 1 [Oreochromis niloticus]
eeel_c14788	+	2.302	0.008	499035754	PREDICTED: isopentenyl-diphosphate Delta-isomerase 1-like isoform X1 [Maylandia zebra]
eeel_c14798	+	1.542	0.036	410903968	PREDICTED: occludin-like [Takifugu rubripes]
eeel_c14820	+	1.769	0.009		
eeel_c14884	+	1.510	0.021	41053927	outer dense fiber protein 3-B [Danio rerio]
eeel_c14900	+	1.989	0.012	348535178	PREDICTED: tubulin beta-1 chain-like [Oreochromis niloticus]
eeel_c14948	+	8.066	0.017	186288308	transketolase-like protein 2 [Danio rerio]
eeel_c15179	+	1.658	0.006	410922044	PREDICTED: leukocyte elastase inhibitor-like [Takifugu rubripes]
eeel_c15206	+	1.737	0.013	326912719	PREDICTED: triosephosphate isomerase-like [Meleagris gallopavo]
eeel_c1536	+	3.849	0.007	410898200	PREDICTED: cocaine- and amphetamine-regulated transcript protein-like [Takifugu rubripes]
eeel_c15456	+	1.727	0.011	242247629	glutathione S-transferase M3 (brain) [Danio rerio]
eeel_c15601	+	1.662	0.011	380036054	proteasome activator complex subunit 1 [Ictalurus punctatus]
eeel_c1570	+	2.652	0.008		
eeel_c1574	+	1.688	0.005	291409522	PREDICTED: DNA directed RNA polymerase II polypeptide G [Oryctolagus cuniculus]
eeel_c16066	+	2.063	0.005	498984298	PREDICTED: sushi von Willebrand factor type A EGF and pentraxin domain-containing protein 1-like [Maylandia zebra]
eeel_c16228	+	1.980	0.026	499048113	PREDICTED: unconventional myosin-Ic-like isoform X3 [Maylandia zebra]
eeel_c16229	+	1.773	0.009	47271415	methylosome subunit pICln [Danio rerio]
eeel_c1624	+	1.913	0.011	363736043	PREDICTED: sodium channel protein type 2 subunit alpha-like isoform 7 [Gallus gallus]
eeel_c16291	+	1.540	0.039	410931036	PREDICTED: dynein heavy chain 9 axonemal-like [Takifugu rubripes]
eeel_c16318	+	1.644	0.036	348525626	PREDICTED: dynactin subunit 3-like [Oreochromis niloticus]
eeel_c16337	+	2.379	0.009		

eeel_c16365	+	2.788	0.013	326923090	PREDICTED: alpha-16-mannosylglycoprotein 6-beta-N-acetylglucosaminyltransferase A-like [Meleagris gallopavo]
eeel_c16382	+	1.550	0.014	155369223	POU domain class 3 transcription factor 4-A [Xenopus laevis]
eeel_c16399	+	1.618	0.013	432921532	PREDICTED: target of rapamycin complex subunit lst8-like [Oryzias latipes]
eeel_c16468	+	1.716	0.039	348517897	PREDICTED: growth arrest and DNA damage-inducible proteins-interacting protein 1-like [Oreochromis niloticus]
eeel_c1657	+	1.888	0.021	291383303	PREDICTED: ribosomal protein S20-like [Oryctolagus cuniculus]
eeel_c16612	+	4.307	0.000	227908753	zwilling [Danio rerio]
eeel_c16625	+	1.787	0.005	213511092	Probable saccharopine dehydrogenase [Salmo salar]
eeel_c16657	+	1.684	0.011	320202999	protein FAM214A [Danio rerio]
eeel_c16661	+	1.806	0.012		
eeel_c16768	+	2.979	0.017	126333881	PREDICTED: iron-sulfur cluster assembly 1 homolog mitochondrial-like [Monodelphis domestica]
eeel_c16788	+	1.628	0.011	397491869	PREDICTED: uncharacterized protein LOC100991169 [Pan paniscus]
eeel_c16857	+	1.766	0.039	54262125	stress-70 protein mitochondrial [Danio rerio]
eeel_c16950	+	1.529	0.000	348508068	PREDICTED: protein kish-B-like [Oreochromis niloticus]
eeel_c1706	+	1.500	0.026		
eeel_c17078	+	2.464	0.013	318102138	membrane-associated progesterone receptor component 1 [Ictalurus punctatus]
eeel_c17087	+	1.691	0.013	432855142	PREDICTED: calcium-binding mitochondrial carrier protein SCaMC-1-like [Oryzias latipes]
eeel_c17113	+	2.506	0.021	192455608	C-type natriuretic peptide 3-like precursor [Danio rerio]
eeel_c17115	+	1.613	0.012	213512026	Alcohol dehydrogenase class-3 [Salmo salar]
eeel_c17238	+	1.529	0.021	348506309	PREDICTED: ADP-ribosylation factor 6-like [Oreochromis niloticus]
eeel_c1726	+	1.898	0.030	348515637	PREDICTED: myosin light chain 1 skeletal muscle isoform-like [Oreochromis niloticus]
eeel_c17505	+	1.642	0.021	213514964	prodynorphin precursor [Salmo salar]
eeel_c1753	+	2.792	0.014	348517883	PREDICTED: enoyl-CoA delta isomerase 1 mitochondrial-like [Oreochromis niloticus]
eeel_c17637	+	1.866	0.011	125829706	PREDICTED: collagen alpha-2(VI) chain [Danio rerio]
eeel_c17706	+	3.758	0.030	498927971	PREDICTED: regulator of G-protein signaling 5-like [Maylandia zebra]
eeel_c17761	+	2.580	0.039	348501150	PREDICTED: aquaporin-1-like [Oreochromis niloticus]
eeel_c18018	+	1.989	0.017	498924960	PREDICTED: calcium/calmodulin-dependent protein kinase kinase 2-like isoform X1 [Maylandia zebra]
eeel_c18071	+	1.850	0.021		
eeel_c18128	+	1.716	0.017	154152155	peptidyl-prolyl cis-trans isomerase FKBP7 precursor [Bos taurus]
eeel_c18195	+	2.027	0.021	327265847	PREDICTED: synapsin-2-like [Anolis carolinensis]
eeel_c18201	+	2.750	0.000		
eeel_c18229	+	2.724	0.017	226443073	Glycophorin-C [Salmo salar]
eeel_c18324	+	2.075	0.036	348510611	PREDICTED: leucine-rich repeat neuronal protein 1-like [Oreochromis niloticus]
eeel_c18489	+	1.745	0.013	259089155	PRA1 family protein 3 [Oncorhynchus mykiss]

eeel_c18511	+	1.587	0.036	432947053	PREDICTED: disheveled-associated activator of morphogenesis 1-like [Oryzias latipes]
eeel_c18604	+	1.889	0.047		
eeel_c18673	+	1.581	0.009	499029111	PREDICTED: transcription factor COE3-like isoform X1 [Maylandia zebra]
eeel_c18770	+	1.595	0.006	403308006	PREDICTED: zinc finger protein 544 isoform 1 [Saimiri boliviensis boliviensis]
eeel_c18808	+	1.867	0.007	291388411	PREDICTED: R-spondin 3-like [Oryctolagus cuniculus]
eeel_c18841	+	2.958	0.024	292619122	PREDICTED: hypothetical protein LOC100331538 [Danio rerio]
eeel_c18855	+	1.505	0.006	312069400	hypothetical protein LOAG_02078 [Loa loa]
eeel_c18947	+	2.278	0.000		
eeel_c18952	+	1.628	0.039	410926105	PREDICTED: diacylglycerol kinase epsilon-like [Takifugu rubripes]
eeel_c18990	+	1.548	0.039	312107035	hypothetical protein LOAG_15297 [Loa loa]
eeel_c19035	+	1.630	0.024	348529764	PREDICTED: N(G)N(G)-dimethylarginine dimethylaminohydrolase 1-like [Oreochromis niloticus]
eeel_c19079	+	1.794	0.039	432950099	PREDICTED: UPF0693 protein C10orf32 homolog [Oryzias latipes]
eeel_c19188	+	1.991	0.013	41282065	protein YIF1A [Danio rerio]
eeel_c19258	+	1.954	0.011		
eeel_c19344	+	1.502	0.039	348529882	PREDICTED: uncharacterized protein C18orf8-like [Oreochromis niloticus]
eeel_c19394	+	1.775	0.013	167527095	hypothetical protein [Monosiga brevicollis MX1]
eeel_c19452	+	2.288	0.006	125840076	PREDICTED: hypothetical protein LOC797003 [Danio rerio]
eeel_c19534	+	1.623	0.024		
eeel_c19582	+	1.616	0.013	224051134	PREDICTED: vacuolar protein sorting-associated protein 18 homolog [Taeniopygia guttata]
eeel_c19734	+	2.491	0.013	498950203	PREDICTED: P2Y purinoceptor 1-like [Maylandia zebra]
eeel_c19772	+	2.273	0.007	395506655	PREDICTED: peroxisomal proliferator-activated receptor A-interacting complex 285 kDa protein-like [Sarcophilus harrisii]
eeel_c19788	+	2.223	0.021	47551321	carboxypeptidase E precursor [Danio rerio]
eeel_c19805	+	2.062	0.030	471380156	PREDICTED: tubulin beta-3 chain [Trichechus manatus latirostris]
eeel_c19829	+	1.630	0.007	499037681	PREDICTED: mammalian ependymin-related protein 1-like [Maylandia zebra]
eeel_c19962	+	1.776	0.017	116267933	integrator complex subunit 9 [Danio rerio]
eeel_c20012	+	2.213	0.016		
eeel_c20012	+	2.011	0.021		
eeel_c20034	+	1.932	0.014	301612261	PREDICTED: uncharacterized protein C9orf135 homolog [Xenopus (Silurana) tropicalis]
eeel_c20057	+	1.748	0.006	113678243	uncharacterized protein LOC767732 [Danio rerio]
eeel_c20166	+	1.520	0.024	432853133	PREDICTED: collagen and calcium-binding EGF domain-containing protein 1-like [Oryzias latipes]
eeel_c20213	+	25.131	0.000	348531190	PREDICTED: cytochrome c oxidase subunit 8A mitochondrial-like [Oreochromis niloticus]
eeel_c20294	+	1.997	0.021	260825062	hypothetical protein BRAFLDRAFT_69918 [Branchiostoma floridae]

eeel_c20387	+	2.117	0.007	348509655	PREDICTED: DEP domain-containing protein 7-like [Oreochromis niloticus]
eeel_c20452	+	1.560	0.036	348506046	PREDICTED: fatty acyl-CoA hydrolase precursor medium chain-like [Oreochromis niloticus]
eeel_c20546	+	1.515	0.007		
eeel_c20619	+	1.527	0.036	122692429	putative deoxyribonuclease TATDN3 [Bos taurus]
eeel_c20691	+	1.669	0.007		
eeel_c20725	+	1.638	0.013	395823162	PREDICTED: LOW QUALITY PROTEIN: eukaryotic initiation factor 4A-II-like [Otolemur garnettii]
eeel_c20743	+	2.221	0.021	327274623	PREDICTED: neuropeptide Y-like [Anolis carolinensis]
eeel_c20768	+	3.469	0.017	326670654	PREDICTED: collagen alpha-3(VI) chain [Danio rerio]
eeel_c20776	+	1.905	0.014		
eeel_c20790	+	1.661	0.011	113682186	uncharacterized protein LOC564374 [Danio rerio]
eeel_c20887	+	2.315	0.008	348525586	PREDICTED: ankyrin repeat and MYND domain-containing protein 2-like [Oreochromis niloticus]
eeel_c20915	+	2.556	0.011	348531124	PREDICTED: laminin subunit alpha-4 [Oreochromis niloticus]
eeel_c20968	+	1.516	0.014	348513532	PREDICTED: aldehyde dehydrogenase family 9 member A1-like [Oreochromis niloticus]
eeel_c20995	+	2.004	0.008	348516455	PREDICTED: NEDD8-activating enzyme E1 regulatory subunit-like isoform 1 [Oreochromis niloticus]
eeel_c20998	+	1.890	0.026		
eeel_c21001	+	2.390	0.013	51972289	alpha/beta hydrolase domain-containing protein 11 [Danio rerio]
eeel_c21128	+	1.603	0.024	410914774	PREDICTED: phosphoglycerate kinase 1-like [Takifugu rubripes]
eeel_c21154	+	1.622	0.017	213515494	Sialic acid synthase [Salmo salar]
eeel_c21219	+	1.567	0.030	327260976	PREDICTED: polyadenylate-binding protein-interacting protein 2-like [Anolis carolinensis]
eeel_c21425	+	1.712	0.009	268607748	leptin receptor overlapping transcript-like 1 [Danio rerio]
eeel_c21444	+	1.613	0.017	47085951	cyclin-G2 [Danio rerio]
eeel_c21461	+	1.846	0.024		
eeel_c21514	+	1.533	0.006		
eeel_c21557	+	1.771	0.026	410895553	PREDICTED: putative pre-mRNA-splicing factor ATP-dependent RNA helicase DHX32-like [Takifugu rubripes]
eeel_c21572	+	1.827	0.011	432880193	PREDICTED: plastin-3-like [Oryzias latipes]
eeel_c21576	+	1.542	0.024	432940858	PREDICTED: activator of 90 kDa heat shock protein ATPase homolog 1-like isoform 1 [Oryzias latipes]
eeel_c2164	+	1.550	0.030	373838782	protachykinin-1 precursor [Danio rerio]
eeel_c21691	+	1.530	0.016		
eeel_c21734	+	2.226	0.036	499010860	PREDICTED: GTPase IMAP family member 8-like [Maylandia zebra]
eeel_c21777	+	1.537	0.009	348535429	PREDICTED: LOW QUALITY PROTEIN: peptidyl-prolyl cis-trans isomerase CWC27 homolog [Oreochromis niloticus]

eeel_c21782	+	1.914	0.007		
eeel_c21802	+	1.843	0.016	192451499	uncharacterized protein LOC558627 [Danio rerio]
eeel_c21922	+	6.389	0.005	348536652	PREDICTED: neuropeptide B-like [Oreochromis niloticus]
eeel_c21989	+	1.878	0.021		
eeel_c22164	+	5.033	0.005	209954616	type III iodothyronine deiodinase a [Takifugu rubripes]
eeel_c2217	+	1.621	0.009		
eeel_c22189	+	2.012	0.030	410925270	PREDICTED: centromere protein M-like [Takifugu rubripes]
eeel_c22453	+	1.674	0.016	62955107	cyclin-dependent kinase 5 activator 2 [Danio rerio]
eeel_c22492	+	1.595	0.016		
eeel_c22508	+	2.160	0.016	348500108	PREDICTED: 39S ribosomal protein L46 mitochondrial-like [Oreochromis niloticus]
eeel_c22538	+	1.554	0.017	326920699	PREDICTED: LOW QUALITY PROTEIN: sec1 family domain-containing protein 1-like partial [Meleagris gallopavo]
eeel_c2255	+	1.797	0.007	432923328	PREDICTED: LYR motif containing protein 1-like [Oryzias latipes]
eeel_c22697	+	1.603	0.009	348537391	PREDICTED: phosphatidylinositol-glycan biosynthesis class W protein-like [Oreochromis niloticus]
eeel_c22724	+	2.059	0.007	348504313	PREDICTED: ELAV-like protein 4-like [Oreochromis niloticus]
eeel_c22745	+	2.418	0.005	62955083	septin-10 [Danio rerio]
eeel_c22775	+	1.871	0.026	395742562	PREDICTED: uncharacterized protein LOC100939189 partial [Pongo abelii]
eeel_c22808	+	1.836	0.005	498966907	PREDICTED: transcription factor AP-2-alpha-like isoform X1 [Maylandia zebra]
eeel_c22832	+	1.521	0.014	148225007	thioredoxin-like 4A [Xenopus laevis]
eeel_c22905	+	2.850	0.009		
eeel_c22918	+	1.954	0.047	348500054	PREDICTED: AKT-interacting protein-like [Oreochromis niloticus]
eeel_c23072	+	1.516	0.047	498968771	PREDICTED: growth arrest-specific protein 1-like [Maylandia zebra]
eeel_c23152	+	1.670	0.039	51010999	corticotropin-releasing factor-binding protein precursor [Danio rerio]
eeel_c23158	+	1.651	0.016	259089201	UPF0235 protein C15orf40 [Oncorhynchus mykiss]
eeel_c23254	+	1.566	0.030	348545410	PREDICTED: hypothetical protein LOC100706256 [Oreochromis niloticus]
eeel_c23267	+	1.828	0.047	348533862	PREDICTED: peptidyl-prolyl cis-trans isomerase FKBP14-like [Oreochromis niloticus]
eeel_c23354	+	1.853	0.017	348523295	PREDICTED: copine-9-like [Oreochromis niloticus]
eeel_c23407	+	1.529	0.017		
eeel_c23427	+	2.007	0.030	348503410	PREDICTED: zinc finger protein ZIC 4-like [Oreochromis niloticus]
eeel_c23483	+	1.558	0.009	291388133	PREDICTED: ubiquitin-conjugating enzyme E2W [Oryctolagus cuniculus]
eeel_c23659	+	2.052	0.008	410902231	PREDICTED: 60S ribosomal protein L3-like [Takifugu rubripes]
eeel_c23699	+	1.689	0.006	499033187	PREDICTED: protocadherin-8-like [Maylandia zebra]
eeel_c23746	+	1.732	0.024	432897347	PREDICTED: BUD13 homolog isoform 1 [Oryzias latipes]
eeel_c23772	+	1.581	0.021		
eeel_c23840	+	1.766	0.039	410931103	PREDICTED: uncharacterized protein LOC101069026 [Takifugu rubripes]
eeel_c23924	+	1.781	0.024	317619899	mu-crystallin homolog [Ictalurus punctatus]
eeel_c24052	+	2.206	0.036	41152050	large proline-rich protein BAT3 [Danio rerio]
eeel_c24072	+	3.258	0.016	94536641	methionine adenosyltransferase II alpha-like [Danio rerio]

eeel_c24095	+	1.927	0.011	242003082	tubulin beta chain putative [Pediculus humanus corporis]
eeel_c24118	+	1.541	0.039	395815487	PREDICTED: reticulocalbin-1 [Otolemur garnettii]
eeel_c2412	+	2.100	0.007		
eeel_c24280	+	1.731	0.024	507551846	PREDICTED: zinc finger protein 420-like [Jaculus jaculus]
eeel_c2451	+	1.640	0.006	213511946	ATP synthase H+ transporting mitochondrial F0 complex subunit c-2 [Salmo salar]
eeel_c24513	+	1.702	0.021	410903638	PREDICTED: electrogenic sodium bicarbonate cotransporter 1-like [Takifugu rubripes]
eeel_c24777	+	1.551	0.017	432865732	PREDICTED: 28S ribosomal protein S25 mitochondrial-like [Oryzias latipes]
eeel_c24819	+	1.836	0.009	185132169	tissue inhibitor of metalloproteinase 2 precursor [Oncorhynchus mykiss]
eeel_c24889	+	2.072	0.011	432947039	PREDICTED: 5-hydroxytryptamine receptor 1B-like [Oryzias latipes]
eeel_c24948	+	2.227	0.011	348522778	PREDICTED: MAGUK p55 subfamily member 6 [Oreochromis niloticus]
eeel_c24952	+	1.680	0.047	213510794	Eukaryotic translation initiation factor 6 [Salmo salar]
eeel_c25043	+	1.618	0.017	348514606	PREDICTED: cytosolic acyl coenzyme A thioester hydrolase-like [Oreochromis niloticus]
eeel_c25059	+	2.584	0.009	498949822	PREDICTED: fibrillin-1-like [Maylandia zebra]
eeel_c251	+	1.637	0.011	348514005	PREDICTED: cathepsin L1-like [Oreochromis niloticus]
eeel_c25217	+	2.622	0.016	410903890	PREDICTED: argininosuccinate synthase-like [Takifugu rubripes]
eeel_c25264	+	2.080	0.026	213515342	NEDD8-activating enzyme E1 catalytic subunit [Salmo salar]
eeel_c2528	+	1.807	0.030	393715121	MHC class I alpha chain precursor [Oncorhynchus mykiss]
eeel_c25393	+	2.070	0.036	498938543	PREDICTED: uncharacterized protein LOC101469977 [Maylandia zebra]
eeel_c25470	+	4.027	0.000	410901026	PREDICTED: homeobox protein HMX2-like [Takifugu rubripes]
eeel_c25528	+	1.509	0.039	50344948	peptidyl-prolyl cis-trans isomerase-like 3 [Danio rerio]
eeel_c25542	+	2.113	0.021	410915900	PREDICTED: thrombomodulin-like [Takifugu rubripes]
eeel_c25675	+	1.920	0.014	348504144	PREDICTED: ras association domain-containing protein 10-like [Oreochromis niloticus]
eeel_c25809	+	1.609	0.013	390176927	GA15288 [Drosophila pseudoobscura pseudoobscura]
eeel_c25826	+	2.449	0.006	410912340	PREDICTED: CD82 antigen-like [Takifugu rubripes]
eeel_c25971	+	2.789	0.012	156380020	predicted protein [Nematostella vectensis]
eeel_c26093	+	2.244	0.039	195110659	GI22823 [Drosophila mojavensis]
eeel_c26172	+	2.119	0.006	348535696	PREDICTED: 28S ribosomal protein S30 mitochondrial-like [Oreochromis niloticus]
eeel_c26331	+	1.638	0.021	239049989	plexin-B2 precursor [Danio rerio]
eeel_c2637	+	1.812	0.014		
eeel_c26476	+	1.811	0.024	292620990	PREDICTED: hypothetical protein LOC100329764 [Danio rerio]
eeel_c26626	+	3.319	0.047	123704523	retinol dehydrogenase 10-A [Danio rerio]
eeel_c2673	+	4.879	0.012	348500050	PREDICTED: iroquois-class homeodomain protein irx-3-like [Oreochromis niloticus]
eeel_c26862	+	1.942	0.007	125821047	PREDICTED: hypothetical protein LOC569091 [Danio rerio]
eeel_c26927	+	1.963	0.021	499029410	PREDICTED: LOW QUALITY PROTEIN: crystallin J1A-like [Maylandia zebra]
eeel_c26959	+	3.005	0.026	167535145	hypothetical protein [Monosiga brevicollis MX1]
eeel_c27016	+	2.012	0.036	432897611	PREDICTED: organic solute transporter subunit alpha-like [Oryzias latipes]
eeel_c27094	+	2.879	0.007	312091502	hypothetical protein LOAG_11435 [Loa loa]

eeel_c2712	+	2.766	0.000	432911317	PREDICTED: myelin protein P0-like [Oryzias latipes]
eeel_c27234	+	1.617	0.024		
eeel_c27273	+	1.616	0.016	50731694	PREDICTED: 24-dienoyl-CoA reductase mitochondrial [Gallus gallus]
eeel_c27311	+	1.932	0.039	326672159	PREDICTED: sc:d805 [Danio rerio]
eeel_c27357	+	2.272	0.009	45361507	uncharacterized protein LOC394955 [Xenopus (Silurana) tropicalis]
eeel_c27485	+	1.730	0.012	432853497	PREDICTED: basigin-like [Oryzias latipes]
eeel_c27583	+	1.582	0.011		
eeel_c27623	+	1.896	0.016	326670632	PREDICTED: high affinity cGMP-specific 3'5'-cyclic phosphodiesterase 9A [Danio rerio]
eeel_c27657	+	2.145	0.017	410916341	PREDICTED: uncharacterized protein KIAA1737 homolog [Takifugu rubripes]
eeel_c27872	+	2.117	0.021	62955347	uncharacterized protein LOC550384 [Danio rerio]
eeel_c27887	+	1.599	0.009	410915900	PREDICTED: thrombomodulin-like [Takifugu rubripes]
eeel_c27908	+	1.639	0.021	157278309	class III alcohol dehydrogenase chi subunit [Oryzias latipes]
eeel_c27949	+	1.546	0.008		
eeel_c27997	+	1.505	0.016	157787169	uncharacterized protein LOC100126028 [Danio rerio]
eeel_c28390	+	1.642	0.008		
eeel_c28468	+	1.862	0.030		
eeel_c2857	+	3.351	0.006	259155106	Transcription factor AP-2 alpha [Salmo salar]
eeel_c28612	+	3.033	0.013	410900015	PREDICTED: glycerol-3-phosphate dehydrogenase [NAD(+)] cytoplasmic-like [Takifugu rubripes]
eeel_c28623	+	1.583	0.014	213514868	protein phosphatase methylesterase 1 [Salmo salar]
eeel_c28841	+	2.523	0.013	291235029	PREDICTED: diphthamide biosynthesis protein 2-like [Saccoglossus kowalevskii]
eeel_c28910	+	1.500	0.036	47086139	signal peptidase complex subunit 3 [Danio rerio]
eeel_c29055	+	2.049	0.014		
eeel_c29184	+	1.588	0.011	348521526	PREDICTED: ceramide kinase-like [Oreochromis niloticus]
eeel_c29277	+	1.624	0.016	192453566	ADP/ATP translocase 3 [Danio rerio]
eeel_c29333	+	1.572	0.021	57525690	nicolin-1 [Danio rerio]
eeel_c29430	+	1.693	0.009	213515260	Methyltransferase-like protein 9 [Salmo salar]
eeel_c29441	+	1.513	0.011	410925100	PREDICTED: putative deoxyribonuclease TATDN1-like [Takifugu rubripes]
eeel_c29464	+	2.388	0.005	291190078	X/potassium-transporting ATPase subunit beta-m [Salmo salar]
eeel_c29506	+	1.760	0.016	348531124	PREDICTED: laminin subunit alpha-4 [Oreochromis niloticus]
eeel_c29507	+	1.650	0.024	52219028	leukocyte receptor cluster (LRC) member 1 [Danio rerio]
eeel_c2951	+	1.810	0.026	292626992	PREDICTED: activity-dependent neuroprotector homeobox protein [Danio rerio]
eeel_c29554	+	3.244	0.006	50345078	sperm-associated antigen 6 [Danio rerio]
eeel_c29678	+	2.727	0.030	301610675	PREDICTED: cocaine- and amphetamine-regulated transcript protein [Xenopus (Silurana) tropicalis]
eeel_c2980	+	1.586	0.039	33504555	dixin-A [Danio rerio]
eeel_c29986	+	2.105	0.011	167533756	hypothetical protein [Monosiga brevicollis MX1]

eeel_c30076	+	1.604	0.016	348535804	PREDICTED: gamma-aminobutyric acid receptor subunit delta-like [Oreochromis niloticus]
eeel_c30116	+	1.718	0.021	185133062	keratin type I cytoskeletal 18 [Oncorhynchus mykiss]
eeel_c30122	+	1.881	0.026	291190803	Periodic tryptophan protein 2 homolog [Salmo salar]
eeel_c30286	+	1.923	0.026	327274830	PREDICTED: adseverin-like [Anolis carolinensis]
eeel_c30331	+	1.905	0.021	18859579	zinc finger protein ZIC 2 [Danio rerio]
eeel_c30346	+	2.055	0.017	499024913	PREDICTED: protein FAM199X-like isoform X1 [Maylandia zebra]
eeel_c3048	+	1.837	0.000	45387807	cofilin-2 [Danio rerio]
eeel_c30570	+	1.868	0.030	498968016	PREDICTED: villin-1-like isoform X1 [Maylandia zebra]
eeel_c30584	+	12.206	0.009	213515450	Pro-MCH 1 precursor [Salmo salar]
eeel_c30763	+	2.119	0.024	194332582	uncharacterized protein LOC100170530 [Xenopus (Silurana) tropicalis]
eeel_c30818	+	2.646	0.039	410903682	PREDICTED: thrombospondin-4-B-like [Takifugu rubripes]
eeel_c30925	+	1.651	0.030	50344806	gastrotropin [Danio rerio]
eeel_c31022	+	2.138	0.024	498965389	PREDICTED: uncharacterized protein LOC101485334 [Maylandia zebra]
eeel_c31104	+	1.679	0.030	213513294	Solute carrier family 25 member 33 [Salmo salar]
eeel_c31166	+	1.508	0.016	504169318	PREDICTED: thioredoxin domain-containing protein 9 [Ochotona princeps]
eeel_c31177	+	1.691	0.007	348541489	PREDICTED: grpE protein homolog 1 mitochondrial-like [Oreochromis niloticus]
eeel_c31293	+	3.276	0.006		
eeel_c31296	+	2.373	0.021	348539728	PREDICTED: forkhead box protein F2-like [Oreochromis niloticus]
eeel_c31303	+	1.541	0.039	410908275	PREDICTED: semaphorin-7A-like [Takifugu rubripes]
eeel_c31322	+	4.136	0.030	116267993	complexin-4 [Danio rerio]
eeel_c3161	+	2.138	0.005	45361345	guanine nucleotide binding protein (G protein) alpha inhibiting activity polypeptide 2 [Xenopus (Silurana) tropicalis]
eeel_c31695	+	2.074	0.024	326674694	PREDICTED: adenylate cyclase type 2 [Danio rerio]
eeel_c31873	+	1.828	0.011		
eeel_c3195	+	1.545	0.024		
eeel_c32081	+	2.437	0.017	499021027	PREDICTED: inorganic pyrophosphatase-like [Maylandia zebra]
eeel_c32164	+	2.384	0.006	125822504	PREDICTED: 3-hydroxyacyl-CoA dehydratase 1 isoform 2 [Danio rerio]
eeel_c32321	+	1.643	0.014	153792478	ran guanine nucleotide release factor [Danio rerio]
eeel_c32394	+	2.283	0.006	116734837	rhombotin-1 [Bos taurus]
eeel_c32444	+	1.670	0.017	74096371	alpha-23-sialyltransferase ST3Gal I [Takifugu rubripes]
eeel_c3247	+	2.101	0.012	56606146	protocadherin 2 alpha b 5 precursor [Danio rerio]
eeel_c32658	+	1.853	0.030	213511977	Annexin A2-A [Salmo salar]
eeel_c32792	+	1.899	0.006		
eeel_c32893	+	1.722	0.007		
eeel_c32922	+	1.574	0.009		
eeel_c32957	+	2.032	0.047	410906805	PREDICTED: calcium-binding protein 4-like [Takifugu rubripes]
eeel_c3307	+	2.151	0.007	50539792	tight junction protein 2b [Danio rerio]
eeel_c33104	+	3.277	0.030	348520766	PREDICTED: protein AHNAK2-like [Oreochromis niloticus]

eeel_c33119	+	2.138	0.009	395541464	PREDICTED: galectin-1-like [Sarcophilus harrisii]
eeel_c33177	+	2.110	0.036	410898840	PREDICTED: protein AHNAK2-like [Takifugu rubripes]
eeel_c33215	+	1.539	0.036	326932650	PREDICTED: proliferating cell nuclear antigen-like [Meleagris gallopavo]
eeel_c33332	+	1.598	0.017		
eeel_c33438	+	4.390	0.009		
eeel_c33438	+	2.924	0.011		
eeel_c33576	+	1.997	0.021	432888026	PREDICTED: serine/threonine-protein kinase ULK1-like [Oryzias latipes]
eeel_c33735	+	36.576	0.016	348530728	PREDICTED: relaxin-3-like [Oreochromis niloticus]
eeel_c33860	+	9.640	0.008	59276036	GS homeobox 1 [Danio rerio]
eeel_c33902	+	5.684	0.017	292609515	PREDICTED: cyclic nucleotide-gated cation channel alpha-3-like [Danio rerio]
eeel_c33997	+	1.546	0.047	403296567	PREDICTED: tubulin alpha-1A chain [Saimiri boliviensis boliviensis]
eeel_c34047	+	1.669	0.039	348532554	PREDICTED: 3-methyl-2-oxobutanoate dehydrogenase [lipoamide] kinase-like [Oreochromis niloticus]
eeel_c34064	+	1.686	0.009	348524346	PREDICTED: 28S ribosomal protein S2 mitochondrial-like [Oreochromis niloticus]
eeel_c34070	+	1.730	0.012	410905517	PREDICTED: chromobox protein homolog 3-like [Takifugu rubripes]
eeel_c34072	+	1.847	0.026	348527266	PREDICTED: claudin-5-like [Oreochromis niloticus]
eeel_c34139	+	1.634	0.030	50539892	mammalian ependymin-related protein 1 precursor [Danio rerio]
eeel_c34352	+	2.002	0.014	326924092	PREDICTED: homeobox protein HMX2-like [Meleagris gallopavo]
eeel_c3436	+	1.860	0.024	410927765	PREDICTED: nebulin-like partial [Takifugu rubripes]
eeel_c34454	+	1.502	0.011	348527680	PREDICTED: vesicle-trafficking protein SEC22b-B-like isoform 2 [Oreochromis niloticus]
eeel_c34717	+	1.518	0.030	326928057	PREDICTED: sodium- and chloride-dependent taurine transporter-like [Meleagris gallopavo]
eeel_c34875	+	1.791	0.030		
eeel_c35049	+	3.532	0.009	332819583	PREDICTED: uncharacterized protein LOC736153 [Pan troglodytes]
eeel_c35053	+	1.553	0.005		
eeel_c3514	+	2.491	0.000	259089115	TRM112-like protein [Oncorhynchus mykiss]
eeel_c35147	+	2.695	0.036		
eeel_c3520	+	2.180	0.005	410917181	PREDICTED: GTP-binding protein Rhes-like [Takifugu rubripes]
eeel_c3531	+	1.579	0.000	224062459	PREDICTED: mitogen-activated protein kinase 6 isoform 1 [Taeniopygia guttata]
eeel_c35365	+	1.559	0.016	301610677	PREDICTED: microtubule-associated protein 1B isoform X1 [Xenopus (Silurana) tropicalis]
eeel_c35432	+	1.816	0.013	61651784	apolipoprotein B mRNA editing enzyme catalytic polypeptide-like 2a [Danio rerio]
eeel_c35496	+	3.199	0.000	499007993	PREDICTED: sodium channel protein type 4 subunit alpha B-like [Maylandia zebra]
eeel_c35601	+	1.756	0.026		
eeel_c3565	+	2.022	0.000	47085709	integral membrane protein 2Bb [Danio rerio]
eeel_c3571	+	1.951	0.008	498974669	PREDICTED: fatty acyl-CoA reductase 1-like isoform X1 [Maylandia zebra]
eeel_c35791	+	1.663	0.039	348542955	PREDICTED: extracellular calcium-sensing receptor-like [Oreochromis niloticus]

eeel_c35822	+	2.000	0.011	488524135	PREDICTED: LOW QUALITY PROTEIN: ras-related protein Rab-6C-like [Dasypus novemcinctus]
eeel_c35878	+	5.530	0.005	194332582	uncharacterized protein LOC100170530 [Xenopus (Silurana) tropicalis]
eeel_c36052	+	1.588	0.047	410925709	PREDICTED: uncharacterized protein LOC101065709 [Takifugu rubripes]
eeel_c36103	+	1.731	0.036	318087606	mitochondrial 28S ribosomal protein s21 [Ictalurus punctatus]
eeel_c36194	+	3.877	0.006	83415106	transcription factor Sox-14 [Danio rerio]
eeel_c36280	+	1.983	0.036	292630016	PREDICTED: hypothetical LOC567907 [Danio rerio]
eeel_c3630	+	1.770	0.006	195546822	phosphatidylcholine transfer protein [Danio rerio]
eeel_c36455	+	1.597	0.009	125995400	SWI/SNF-related matrix-associated actin-dependent regulator of chromatin subfamily A member 5 [Danio rerio]
eeel_c3653	+	1.673	0.006	348581674	PREDICTED: stress-associated endoplasmic reticulum protein 1-like [Cavia porcellus]
eeel_c36578	+	1.569	0.005	410908331	PREDICTED: sorting nexin-1-like [Takifugu rubripes]
eeel_c36630	+	1.573	0.036	50539696	ras-related protein Rab-15 [Danio rerio]
eeel_c3691	+	1.748	0.006	50080160	SPARC precursor [Danio rerio]
eeel_c36943	+	1.612	0.005	167534850	hypothetical protein [Monosiga brevicollis MX1]
eeel_c37181	+	1.630	0.047	185133090	CD59-like protein 2 precursor [Oncorhynchus mykiss]
eeel_c37309	+	2.286	0.039	348502232	PREDICTED: receptor-type tyrosine-protein phosphatase eta-like [Oreochromis niloticus]
eeel_c37362	+	2.876	0.047	426378219	PREDICTED: protein AHNAK2 [Gorilla gorilla gorilla]
eeel_c37364	+	7.326	0.005	194332582	uncharacterized protein LOC100170530 [Xenopus (Silurana) tropicalis]
eeel_c37396	+	2.259	0.039	68367270	PREDICTED: solute carrier family 46 member 3 [Danio rerio]
eeel_c37573	+	1.824	0.036	156550769	PREDICTED: thioredoxin-like protein 4A-like [Nasonia vitripennis]
eeel_c3761	+	1.699	0.007	319803101	uncharacterized protein C1orf198 homolog [Danio rerio]
eeel_c37616	+	2.143	0.030	326676262	PREDICTED: neuroblast differentiation-associated protein AHNAK [Danio rerio]
eeel_c37658	+	1.753	0.014	432855382	PREDICTED: RNA 3'-terminal phosphate cyclase-like [Oryzias latipes]
eeel_c37703	+	1.740	0.026	348520144	PREDICTED: protein TEX261-like [Oreochromis niloticus]
eeel_c3771	+	3.028	0.047	392347864	PREDICTED: uncharacterized protein LOC100910862 [Rattus norvegicus]
eeel_c37762	+	1.974	0.006	61806620	immunoglobulin superfamily containing leucine-rich repeat protein 2 precursor [Danio rerio]
eeel_c37856	+	1.629	0.030	348531222	PREDICTED: tetratricopeptide repeat protein 8-like isoform 1 [Oreochromis niloticus]
eeel_c37959	+	1.587	0.007	290543474	guanine nucleotide-binding protein G(i) subunit alpha-1 [Cavia porcellus]
eeel_c3800	+	1.680	0.021	121583950	arrestin domain-containing protein 3 [Danio rerio]
eeel_c38014	+	1.606	0.017		
eeel_c38014	+	1.533	0.021		
eeel_c38026	+	2.292	0.013	432866132	PREDICTED: uncharacterized protein LOC101162600 [Oryzias latipes]
eeel_c38043	+	2.381	0.024	499007400	PREDICTED: germ cell-specific gene 1-like protein-like [Maylandia zebra]
eeel_c38181	+	1.939	0.024	410896764	PREDICTED: transcription factor Sox-21-B-like [Takifugu rubripes]
eeel_c38279	+	1.814	0.026	292627432	PREDICTED: phospholipid scramblase 2 [Danio rerio]

eeel_c38325	+	2.810	0.013	348518002	PREDICTED: UV excision repair protein RAD23 homolog A-like [Oreochromis niloticus]
eeel_c38338	+	1.556	0.012		
eeel_c38355	+	2.185	0.012	41055335	calpain 1 (mu/I) large subunit a [Danio rerio]
eeel_c38373	+	1.628	0.024	498945774	PREDICTED: HIG1 domain family member 1A mitochondrial-like [Maylandia zebra]
eeel_c38448	+	1.572	0.024	348521980	PREDICTED: gamma-soluble NSF attachment protein-like [Oreochromis niloticus]
eeel_c38780	+	2.770	0.005	50345078	sperm-associated antigen 6 [Danio rerio]
eeel_c38781	+	1.537	0.014	348511769	PREDICTED: signal recognition particle 72 kDa protein [Oreochromis niloticus]
eeel_c38853	+	1.533	0.021	54400362	threonyl-tRNA synthetase cytoplasmic isoform 2 [Danio rerio]
eeel_c38916	+	1.946	0.011	348507867	PREDICTED: 39S ribosomal protein L20 mitochondrial-like [Oreochromis niloticus]
eeel_c39138	+	2.025	0.014	293347225	PREDICTED: serine/threonine-protein kinase Sgk3 isoform 2 [Rattus norvegicus]
eeel_c39180	+	1.552	0.047	410904725	PREDICTED: uncharacterized protein LOC101062688 [Takifugu rubripes]
eeel_c39191	+	1.742	0.024	326675152	PREDICTED: r-spondin-2 [Danio rerio]
eeel_c39265	+	1.528	0.016	47087391	1-acyl-sn-glycerol-3-phosphate acyltransferase gamma [Danio rerio]
eeel_c39351	+	12.472	0.006	126325843	PREDICTED: transcription factor SOX-14-like [Monodelphis domestica]
eeel_c39383	+	2.374	0.011	147904266	lysyl oxidase precursor [Xenopus laevis]
eeel_c39488	+	1.793	0.013	18859023	mesoderm specific transcript [Danio rerio]
eeel_c39636	+	1.530	0.024	47271415	methylosome subunit pICln [Danio rerio]
eeel_c39637	+	1.832	0.008	432860117	PREDICTED: uncharacterized protein LOC101170671 [Oryzias latipes]
eeel_c39637	+	1.837	0.013	432860117	PREDICTED: uncharacterized protein LOC101170671 [Oryzias latipes]
eeel_c39751	+	3.736	0.007	348538840	PREDICTED: myelin proteolipid protein-like isoform 1 [Oreochromis niloticus]
eeel_c39813	+	3.257	0.011		
eeel_c39835	+	3.416	0.006		
eeel_c39902	+	1.693	0.013	148226704	A-kinase anchor protein 12 [Danio rerio]
eeel_c40115	+	2.248	0.011	348507517	PREDICTED: zyxin-like [Oreochromis niloticus]
eeel_c40124	+	2.486	0.011	18858815	homeobox protein HMX3 [Danio rerio]
eeel_c40142	+	2.751	0.006		
eeel_c40149	+	2.169	0.013	348516816	PREDICTED: myelin proteolipid protein-like [Oreochromis niloticus]
eeel_c4018	+	3.791	0.006	499000985	PREDICTED: neurofilament light polypeptide-like [Maylandia zebra]
eeel_c40536	+	5.260	0.012	292617392	PREDICTED: si:ch73-250a16.4 [Danio rerio]
eeel_c40637	+	1.829	0.012	498993403	PREDICTED: alcohol dehydrogenase [NADP(+)] B-like isoform X2 [Maylandia zebra]
eeel_c40667	+	1.666	0.016		
eeel_c40725	+	1.512	0.005		
eeel_c40830	+	1.669	0.024	326680368	PREDICTED: hypothetical protein LOC561967 [Danio rerio]
eeel_c41023	+	1.555	0.047	115496228	regulator of G-protein signaling 7-binding protein A [Danio rerio]
eeel_c41076	+	1.682	0.017	325296962	IQ and AAA domain-containing protein-like [Danio rerio]
eeel_c41119	+	1.816	0.021	432880409	PREDICTED: GPI ethanolamine phosphate transferase 2-like [Oryzias latipes]
eeel_c4130	+	2.757	0.036	213512537	betaine--homocysteine S-methyltransferase 1 [Salmo salar]
eeel_c4140	+	1.688	0.036	312105679	hypothetical protein LOAG_15015 [Loa loa]

eeel_c41421	+	1.535	0.036	488580272	PREDICTED: mediator of RNA polymerase II transcription subunit 17 [Dasypus novemcinctus]
eeel_c41969	+	2.025	0.009	499014505	PREDICTED: fructose-bisphosphate aldolase C-like [Maylandia zebra]
eeel_c4199	+	1.813	0.012	348503426	PREDICTED: ubiquitin-like modifier-activating enzyme 5-like [Oreochromis niloticus]
eeel_c42073	+	1.665	0.016	410922345	PREDICTED: fibroblast growth factor receptor 1-A-like isoform 4 [Takifugu rubripes]
eeel_c42085	+	1.536	0.024	348522304	PREDICTED: low molecular weight neuronal intermediate filament-like [Oreochromis niloticus]
eeel_c42410	+	3.250	0.000		
eeel_c42688	+	1.900	0.024	291414531	PREDICTED: PCI domain containing 2 partial [Oryctolagus cuniculus]
eeel_c42754	+	1.803	0.000	348520690	PREDICTED: gremlin-1-like [Oreochromis niloticus]
eeel_c42783	+	1.897	0.024		
eeel_c42887	+	3.403	0.017	326679451	PREDICTED: nuclear factor 7 ovary [Danio rerio]
eeel_c42893	+	1.614	0.024	466061495	PREDICTED: uncharacterized protein LOC101285118 [Orcinus orca]
eeel_c43156	+	2.097	0.011	348532809	PREDICTED: iron-sulfur cluster assembly enzyme ISCU mitochondrial-like [Oreochromis niloticus]
eeel_c43514	+	2.144	0.012	210147423	proteasome 26S non-ATPase subunit 5 [Danio rerio]
eeel_c43760	+	1.598	0.007	87162449	protein canopy-1 precursor [Danio rerio]
eeel_c43820	+	1.623	0.030		
eeel_c44068	+	1.541	0.047	113680231	iroquois-class homeodomain protein IRX-5 [Danio rerio]
eeel_c4417	+	1.607	0.017	348508100	PREDICTED: myosin regulatory light polypeptide 9-like [Oreochromis niloticus]
eeel_c44175	+	1.687	0.008	348510123	PREDICTED: 26S proteasome non-ATPase regulatory subunit 3-like [Oreochromis niloticus]
eeel_c44354	+	1.637	0.014	292619425	PREDICTED: mismatch repair endonuclease PMS2 [Danio rerio]
eeel_c4452	+	1.598	0.009	94536912	probable ribosome biogenesis protein NEP1 [Danio rerio]
eeel_c44651	+	1.731	0.036	318085579	rho gdp-dissociation inhibitor 2 [Ictalurus punctatus]
eeel_c44806	+	1.538	0.021	18859023	mesoderm specific transcript [Danio rerio]
eeel_c44863	+	10.516	0.005	125832213	PREDICTED: carbonic anhydrase 9 [Danio rerio]
eeel_c44875	+	1.923	0.014		
eeel_c44885	+	1.547	0.026	348503452	PREDICTED: 3-hydroxyacyl-CoA dehydratase 1-like [Oreochromis niloticus]
eeel_c4504	+	1.674	0.026	41055540	PDZ domain-containing protein GIPC2 [Danio rerio]
eeel_c45090	+	2.392	0.006	410929053	PREDICTED: DEP domain-containing protein 7-like [Takifugu rubripes]
eeel_c45142	+	1.606	0.026		
eeel_c45216	+	2.217	0.017	113673092	nuclear receptor 2C2-associated protein [Danio rerio]
eeel_c4531	+	2.003	0.009	213514204	Transmembrane protein 186 [Salmo salar]
eeel_c45322	+	1.872	0.006	348521460	PREDICTED: 3-beta-hydroxysteroid-Delta(8)Delta(7)-isomerase-like [Oreochromis niloticus]
eeel_c45361	+	1.572	0.013	410900468	PREDICTED: leucine-rich repeat flightless-interacting protein 2-like [Takifugu rubripes]
eeel_c45417	+	2.416	0.016	291405581	PREDICTED: DNA ligase (ATP) 3 isoform 1 [Oryctolagus cuniculus]

eeel_c45566	+	2.070	0.008	432861674	PREDICTED: dnaJ homolog subfamily A member 4-like [Oryzias latipes]
eeel_c45756	+	2.526	0.000	482677675	hairy and enhancer of split 5-like [Gallus gallus]
eeel_c45765	+	1.667	0.039	410899354	PREDICTED: alpha-enolase-like isoform 1 [Takifugu rubripes]
eeel_c45866	+	1.828	0.030	68371322	PREDICTED: ADM-like [Danio rerio]
eeel_c46072	+	2.001	0.005		
eeel_c46115	+	2.141	0.014	125833481	PREDICTED: glycine N-acyltransferase-like protein 3 [Danio rerio]
eeel_c4612	+	1.515	0.016	47087251	sestrin-3 [Danio rerio]
eeel_c46155	+	2.056	0.024	327266334	PREDICTED: copine-9-like [Anolis carolinensis]
eeel_c4645	+	2.558	0.006		
eeel_c46607	+	1.522	0.036	213515440	actin-related protein 8 [Salmo salar]
eeel_c46651	+	2.197	0.039	116517260	uncharacterized protein LOC565195 [Danio rerio]
eeel_c46693	+	1.793	0.026	62955193	survival of motor neuron protein-interacting protein 1 [Danio rerio]
eeel_c46829	+	5.003	0.024		
eeel_c4693	+	1.516	0.005	213514364	Vascular endothelial growth factor D precursor [Salmo salar]
eeel_c47147	+	3.226	0.013		
eeel_c4731	+	3.524	0.005	348528238	PREDICTED: LIM domain only protein 7-like [Oreochromis niloticus]
eeel_c47402	+	1.997	0.012	499042800	PREDICTED: titin-like [Maylandia zebra]
eeel_c476	+	1.688	0.036	348517239	PREDICTED: ATP synthase subunit alpha mitochondrial-like [Oreochromis niloticus]
eeel_c47704	+	5.081	0.009	113680231	iroquois-class homeodomain protein IRX-5 [Danio rerio]
eeel_c4771	+	1.670	0.005	185136247	endothelial differentiation-related factor 1 homolog [Oncorhynchus mykiss]
eeel_c4773	+	1.514	0.012	348510773	PREDICTED: centromere protein O-like [Oreochromis niloticus]
eeel_c47780	+	1.568	0.036	213513704	hydroxysteroid dehydrogenase-like protein 2 [Salmo salar]
eeel_c4788	+	1.912	0.011	113678602	protein FAM206A [Danio rerio]
eeel_c47988	+	1.827	0.016	126327738	PREDICTED: serine/threonine-protein kinase PAK 1-like isoform 1 [Monodelphis domestica]
eeel_c48020	+	2.567	0.021	505851525	PREDICTED: stanniocalcin-1 [Sorex araneus]
eeel_c48431	+	2.385	0.026	157841270	uncharacterized protein LOC559351 [Danio rerio]
eeel_c4845	+	1.642	0.047	432927895	PREDICTED: COP9 signalosome complex subunit 5-like [Oryzias latipes]
eeel_c48482	+	1.840	0.047	296217536	PREDICTED: switch-associated protein 70 isoform 1 [Callithrix jacchus]
eeel_c48532	+	3.058	0.000	113671514	stimulated by retinoic acid gene 6 homolog [Danio rerio]
eeel_c48885	+	2.482	0.012	348531170	PREDICTED: homeobox protein SIX1-like [Oreochromis niloticus]
eeel_c4912	+	1.824	0.026	348537812	PREDICTED: lysosomal alpha-glucosidase-like [Oreochromis niloticus]
eeel_c4921	+	2.561	0.011	197099510	tropomyosin alpha-3 chain [Pongo abelii]
eeel_c49239	+	1.516	0.039	194036729	PREDICTED: trimethylguanosine synthase [Sus scrofa]
eeel_c49335	+	1.673	0.021	48762657	alpha-enolase [Danio rerio]
eeel_c49384	+	1.627	0.024	68366774	PREDICTED: 80 kDa MCM3-associated protein [Danio rerio]
eeel_c49549	+	1.965	0.005	498956597	PREDICTED: iron-sulfur cluster assembly enzyme ISCU mitochondrial-like [Maylandia zebra]
eeel_c49557	+	2.091	0.005	41054467	riboflavin transporter 1 [Danio rerio]

eeel_c4959	+	2.353	0.006		
eeel_c4969	+	11.605	0.013	498996654	PREDICTED: fibroblast growth factor-binding protein 2-like [Maylandia zebra]
eeel_c4972	+	1.770	0.007		
eeel_c4972	+	2.141	0.012		
eeel_c49969	+	1.534	0.039	213515554	kaptin [Salmo salar]
eeel_c50188	+	2.986	0.016		
eeel_c50188	+	1.872	0.030		
eeel_c5026	+	1.708	0.047	499023480	PREDICTED: phosphorylase b kinase gamma catalytic chain liver/testis isoform-like [Maylandia zebra]
eeel_c50369	+	1.534	0.030		
eeel_c5046	+	1.547	0.036	348510381	PREDICTED: NEDD8-activating enzyme E1 catalytic subunit-like isoform 1 [Oreochromis niloticus]
eeel_c50594	+	1.871	0.013	348533247	PREDICTED: probable tRNA pseudouridine synthase 1-like [Oreochromis niloticus]
eeel_c50595	+	1.551	0.009	213511606	kynurenine formamidase [Salmo salar]
eeel_c5066	+	2.331	0.000		
eeel_c5066	+	2.002	0.021		
eeel_c50749	+	1.637	0.009	156379369	predicted protein [Nematostella vectensis]
eeel_c50995	+	9.778	0.017	348532269	PREDICTED: galanin peptides-like isoform 1 [Oreochromis niloticus]
eeel_c51084	+	1.673	0.011	410900646	PREDICTED: exosome complex exonuclease RRP44-like [Takifugu rubripes]
eeel_c51281	+	2.036	0.000		
eeel_c51722	+	2.894	0.006	296223709	PREDICTED: actin cytoplasmic 2-like isoform 1 [Callithrix jacchus]
eeel_c51828	+	1.664	0.013	348534130	PREDICTED: hexokinase-1 [Oreochromis niloticus]
eeel_c51951	+	1.660	0.016	18858803	transcription factor HES-1 [Danio rerio]
eeel_c5248	+	1.688	0.014	46309499	alpha/beta hydrolase domain-containing protein 14A [Danio rerio]
eeel_c5334	+	2.216	0.024	162138982	serine protease HTRA1B precursor [Danio rerio]
eeel_c534	+	5.073	0.007	50345022	small muscular protein [Danio rerio]
eeel_c5371	+	2.081	0.017		
eeel_c5512	+	4.630	0.017	71896525	iroquois-class homeodomain protein IRX-2 [Gallus gallus]
eeel_c5532	+	1.582	0.000	318131932	isocitrate dehydrogenase [NAD] subunit alpha mitochondrial [Ictalurus punctatus]
eeel_c5534	+	1.546	0.036	213511204	ER membrane protein complex subunit 4 [Salmo salar]
eeel_c5617	+	1.632	0.000	326668207	PREDICTED: cytospin-B [Danio rerio]
eeel_c56673	+	2.057	0.014	185132790	retinol-binding protein 2 precursor [Oncorhynchus mykiss]
eeel_c57311	+	1.819	0.036	55742511	ubiquitin fusion degradation protein 1 homolog [Xenopus (Silurana) tropicalis]
eeel_c5751	+	1.518	0.026		
eeel_c5807	+	2.112	0.017	301607381	PREDICTED: hypothetical protein LOC100496145 [Xenopus (Silurana) tropicalis]
eeel_c5834	+	1.639	0.036	318037426	mitogen-activated protein kinase scaffold protein 1 [Ictalurus punctatus]
eeel_c593	+	1.506	0.017	291397244	PREDICTED: peroxiredoxin 6 [Oryctolagus cuniculus]
eeel_c5952	+	1.943	0.009		
eeel_c603	+	1.734	0.006	410931036	PREDICTED: dynein heavy chain 9 axonemal-like [Takifugu rubripes]

eeel_c6069	+	1.703	0.007	499045768	PREDICTED: phosphorylase b kinase regulatory subunit alpha skeletal muscle isoform-like isoform X1 [Maylandia zebra]
eeel_c6136	+	1.592	0.021	410900352	PREDICTED: UPF0693 protein C10orf32 homolog [Takifugu rubripes]
eeel_c6159	+	1.592	0.008	41152441	dehydrogenase/reductase (SDR family) member 9 [Danio rerio]
eeel_c621	+	1.864	0.024	498981227	PREDICTED: collagen alpha-1(XI) chain-like [Maylandia zebra]
eeel_c624	+	1.506	0.039	432868564	PREDICTED: protein HEXIM-like [Oryzias latipes]
eeel_c628	+	1.935	0.013	238776821	proteolipid protein 2 [Danio rerio]
eeel_c6321	+	1.530	0.013	348513460	PREDICTED: ras-related protein Rab-6B-like [Oreochromis niloticus]
eeel_c6322	+	3.083	0.000	50345042	response gene to complement 32 protein [Danio rerio]
eeel_c6332	+	1.641	0.006		
eeel_c6412	+	4.662	0.036	185133699	collagen 1a1 precursor [Oncorhynchus mykiss]
eeel_c6445	+	1.947	0.000	348527872	PREDICTED: solute carrier family 22 member 5-like [Oreochromis niloticus]
eeel_c6560	+	2.030	0.000	292619961	PREDICTED: hypothetical protein LOC100332367 [Danio rerio]
eeel_c6605	+	5.284	0.039	348500124	PREDICTED: insulin gene enhancer protein ISL-3-like [Oreochromis niloticus]
eeel_c6683	+	2.017	0.016	326665952	PREDICTED: heat shock 70 kDa protein-like [Danio rerio]
eeel_c6723	+	2.251	0.014	99028957	homeobox protein unc-4 homolog [Danio rerio]
eeel_c6751	+	1.569	0.026	348533107	PREDICTED: glutathione peroxidase 7-like [Oreochromis niloticus]
eeel_c6792	+	1.563	0.000		
eeel_c6862	+	2.860	0.011		
eeel_c6867	+	1.545	0.039	348501228	PREDICTED: proteasome subunit alpha type-7-like isoform 1 [Oreochromis niloticus]
eeel_c6951	+	1.584	0.036	507634559	PREDICTED: MOSC domain-containing protein 1 mitochondrial [Echinops telfairi]
eeel_c6975	+	2.900	0.005	348503530	PREDICTED: inositol monophosphatase 1-like [Oreochromis niloticus]
eeel_c6998	+	2.180	0.016	326679023	PREDICTED: hypothetical protein LOC557000 [Danio rerio]
eeel_c7001	+	1.564	0.014	192455648	uncharacterized protein LOC561073 precursor [Danio rerio]
eeel_c7125	+	1.722	0.000		
eeel_c717	+	1.522	0.036	185132832	DNA-binding protein inhibitor ID-1 [Oncorhynchus mykiss]
eeel_c7238	+	2.937	0.047	301774272	PREDICTED: tropomyosin alpha-3 chain-like isoform 3 [Ailuropoda melanoleuca]
eeel_c7258	+	4.355	0.012	348526229	PREDICTED: C-type natriuretic peptide 1-like [Oreochromis niloticus]
eeel_c73	+	2.052	0.047	410903878	PREDICTED: cytochrome c oxidase subunit 5B mitochondrial-like [Takifugu rubripes]
eeel_c7325	+	1.710	0.011	348539526	PREDICTED: 12-dihydroxy-3-keto-5-methylthiopentene dioxygenase-like [Oreochromis niloticus]
eeel_c7423	+	2.185	0.026	300795671	UDP glucuronosyltransferase 1 family polypeptide a3 precursor [Danio rerio]
eeel_c7426	+	3.236	0.013	348519459	PREDICTED: myelin basic protein-like [Oreochromis niloticus]
eeel_c7427	+	1.609	0.008		
eeel_c7457	+	1.928	0.007	498998900	PREDICTED: WAP four-disulfide core domain protein 3-like partial [Maylandia zebra]
eeel_c7460	+	1.578	0.000	348512408	PREDICTED: succinate dehydrogenase [ubiquinone] flavoprotein subunit mitochondrial-like isoform 1 [Oreochromis niloticus]
eeel_c7498	+	1.534	0.047	348532177	PREDICTED: leukocyte elastase inhibitor-like [Oreochromis niloticus]
eeel_c7511	+	1.986	0.006	198474772	GA25691 [Drosophila pseudoobscura pseudoobscura]

eeel_c7672	+	1.719	0.006	47271382	CD9 antigen [Danio rerio]
eeel_c8036	+	1.724	0.005		
eeel_c8172	+	1.833	0.014	410895129	PREDICTED: cytochrome C oxidase assembly factor 3 homolog mitochondrial-like [Takifugu rubripes]
eeel_c8228	+	1.543	0.009	268607579	trafficking protein particle complex 2-like [Danio rerio]
eeel_c8273	+	1.759	0.014	301774272	PREDICTED: tropomyosin alpha-3 chain-like isoform 3 [Ailuropoda melanoleuca]
eeel_c8302	+	1.628	0.036		
eeel_c8498	+	4.332	0.006		
eeel_c8569	+	1.749	0.000	499024983	PREDICTED: probable RNA-binding protein EIF1AD-like isoform X1 [Maylandia zebra]
eeel_c8578	+	1.627	0.008	499031856	PREDICTED: microsomal glutathione S-transferase 3-like [Maylandia zebra]
eeel_c8658	+	2.362	0.030	50540138	protein phosphatase 1 regulatory subunit 1C [Danio rerio]
eeel_c8719	+	2.231	0.024		
eeel_c8759	+	1.717	0.011		
eeel_c878	+	2.141	0.030	213514432	Neurogenic differentiation factor 1 [Salmo salar]
eeel_c8795	+	2.035	0.024		
eeel_c8936	+	1.828	0.006	41054860	probable RNA-binding protein EIF1AD [Danio rerio]
eeel_c899	+	2.093	0.011	348515437	PREDICTED: NADH dehydrogenase [ubiquinone] 1 alpha subcomplex subunit 1-like isoform 1 [Oreochromis niloticus]
eeel_c9005	+	4.210	0.039		
eeel_c9215	+	1.864	0.016	56790260	mitochondrial uncoupling protein 2 [Danio rerio]
eeel_c9246	+	1.657	0.000		
eeel_c930	+	1.728	0.036	213510962	Transmembrane protein 126A [Salmo salar]
eeel_c9309	+	1.679	0.014	338723081	PREDICTED: dynein light chain roadblock-type 2-like [Equus caballus]
eeel_c950	+	2.374	0.047	498999976	PREDICTED: probable G-protein coupled receptor 125-like [Maylandia zebra]
eeel_c9526	+	9.036	0.014	498961906	PREDICTED: beta-crystallin A3-2-like [Maylandia zebra]
eeel_c973	+	1.764	0.000	47087187	MID1 interacting G12-like protein [Gallus gallus]
eeel_c9797	+	1.526	0.024	348534158	PREDICTED: probable saccharopine dehydrogenase-like [Oreochromis niloticus]
eeel_c9833	+	2.405	0.009	317575728	protachykinin-1 precursor [Ictalurus punctatus]
eeel_c9841	+	1.514	0.036	224060167	PREDICTED: zinc finger protein ZIC 1 [Taeniopygia guttata]
eeel_rep_c15837	+	3.573	0.000	185132790	retinol-binding protein 2 precursor [Oncorhynchus mykiss]
eeel_rep_c16022	+	1.947	0.021	498949521	PREDICTED: BTB/POZ domain-containing protein 1-like isoform X2 [Maylandia zebra]
eeel_rep_c16089	+	2.426	0.011	126332066	PREDICTED: protein S100-P-like [Monodelphis domestica]
eeel_rep_c16493	+	1.503	0.017	50539990	thioredoxin [Danio rerio]
eeel_rep_c17239	+	1.660	0.021	58391145	AGAP003935-PA [Anopheles gambiae str. PEST]
eeel_rep_c18733	+	1.633	0.030	27545193	brain creatine kinase b [Danio rerio]
eeel_rep_c18740	+	1.651	0.000	499018076	PREDICTED: cystatin-like [Maylandia zebra]
eeel_rep_c18832	+	1.955	0.007	47086689	glutathione S-transferase M [Danio rerio]
eeel_rep_c18950	+	2.288	0.024	348540479	PREDICTED: SLAM family member 7-like [Oreochromis niloticus]
eeel_rep_c18959	+	1.720	0.012		

eeel_rep_c19217	+	1.965	0.007	395742562	PREDICTED: uncharacterized protein LOC100939189 partial [Pongo abelii]
eeel_rep_c19927	+	1.513	0.007	160420173	transmembrane protein 144 [Xenopus laevis]
eeel_rep_c22199	+	2.487	0.021	301777660	PREDICTED: myosin-7-like [Ailuropoda melanoleuca]
eeel_rep_c22317	+	2.823	0.021		
eeel_rep_c22430	+	2.006	0.012	326668568	PREDICTED: gelsolin-like [Danio rerio]
eeel_rep_c23470	+	1.578	0.005	47271372	ATP synthase H+ transporting mitochondrial F0 complex subunit c3 (subunit 9) genome duplicate b [Danio rerio]
eeel_rep_c24494	+	2.476	0.013		
eeel_rep_c26213	+	1.927	0.024		
eeel_rep_c27247	+	2.072	0.036	318103615	brain protein 44-like protein [Ictalurus punctatus]
eeel_rep_c27586	+	3.937	0.016	326932072	PREDICTED: troponin C skeletal muscle-like isoform 1 [Meleagris gallopavo]
eeel_rep_c30053	+	3.110	0.005	390468192	PREDICTED: 60S ribosomal protein L7a-like partial [Callithrix jacchus]
eeel_rep_c30205	+	2.535	0.014	395852832	PREDICTED: myosin-7-like [Otolemur garnettii]
eeel_rep_c30222	+	3.661	0.007	410896648	PREDICTED: uncharacterized protein LOC101068355 [Takifugu rubripes]
eeel_rep_c30490	+	1.910	0.013	194578927	cytochrome c oxidase subunit VIIb [Danio rerio]
eeel_rep_c31725	+	1.549	0.000	498937099	PREDICTED: keratinocyte-associated protein 2-like isoform X1 [Maylandia zebra]
eeel_rep_c33690	+	1.613	0.009	432849647	PREDICTED: ATP synthase subunit beta mitochondrial-like [Oryzias latipes]
eeel_rep_c34058	+	1.563	0.005	213512117	ATP synthase H+ transporting mitochondrial F0 complex subunit c-3 [Salmo salar]
eeel_rep_c34189	+	3.918	0.047	488594051	PREDICTED: carboxypeptidase E [Dasypus novemcinctus]
eeel_rep_c34453	+	1.525	0.030	157838025	N-acetylated-alpha-linked acidic dipeptidase-like protein [Danio rerio]
eeel_rep_c34463	+	1.677	0.000	348519669	PREDICTED: ATP synthase lipid-binding protein mitochondrial-like isoform 1 [Oreochromis niloticus]
eeel_rep_c34618	+	2.995	0.012	226443324	S100 calcium binding protein beta (neural) [Salmo salar]
eeel_rep_c35128	+	2.114	0.013	432907852	PREDICTED: sodium- and chloride-dependent transporter XTRP3-like isoform 1 [Oryzias latipes]
eeel_rep_c36311	+	3.668	0.012	291388127	PREDICTED: retinol dehydrogenase 10 [Oryctolagus cuniculus]
eeel_rep_c37622	+	1.512	0.047		
eeel_rep_c38685	+	2.430	0.011	327280691	PREDICTED: serine/threonine-protein phosphatase 4 catalytic subunit-like isoform 1 [Anolis carolinensis]
eeel_rep_c40332	+	1.726	0.006	226443324	S100 calcium binding protein beta (neural) [Salmo salar]
eeel_rep_c40664	+	2.101	0.017	318056044	s100-a10 [Ictalurus punctatus]
eeel_rep_c41182	+	1.754	0.009		
eeel_rep_c42053	+	1.853	0.011		
eeel_rep_c42748	+	3.712	0.000	488516138	PREDICTED: retinol-binding protein 4 isoform 1 [Dasypus novemcinctus]
eeel_rep_c43707	+	1.782	0.006	326921722	PREDICTED: sorcin-like [Meleagris gallopavo]
eeel_rep_c45427	+	1.665	0.024	499035953	PREDICTED: cysteine-rich venom protein ablomin-like [Maylandia zebra]
eeel_rep_c45910	+	1.899	0.007	213514118	Fatty acid-binding protein adipocyte [Salmo salar]
eeel_rep_c45950	+	1.587	0.026	297292234	PREDICTED: hypothetical protein LOC100424028 [Macaca mulatta]

eeel_rep_c45959	+	2.431	0.021	301616885	PREDICTED: uncharacterized protein DKFZp762I1415-like [Xenopus (Silurana) tropicalis]
eeel_rep_c47566	+	1.919	0.017	170074060	histone H2B.1 [Culex quinquefasciatus]
eeel_rep_c48799	+	4.912	0.030		
eeel_rep_c50854	+	1.740	0.030	301603650	PREDICTED: UPF0587 protein C1orf123 homolog [Xenopus (Silurana) tropicalis]
eeel_rep_c51291	+	4.188	0.047		
eeel_rep_c51422	+	2.017	0.005	185134464	peroxiredoxin [Oncorhynchus mykiss]
eeel_rep_c51896	+	2.013	0.008	213514682	Musculoskeletal embryonic nuclear protein 1 [Salmo salar]
eeel_rep_c55052	+	1.565	0.039	326922893	PREDICTED: tubulin alpha-4A chain-like [Meleagris gallopavo]
eeel_rep_c55508	+	2.194	0.047	213512718	LisH domain-containing protein C16orf63 homolog [Salmo salar]
eeel_rep_c55602	+	1.927	0.016	41055054	thymocyte nuclear protein 1 [Danio rerio]
eeel_rep_c55634	+	1.906	0.021	125851982	PREDICTED: troponin C skeletal muscle [Danio rerio]
eeel_rep_c55681	+	1.621	0.009	499017111	PREDICTED: calmodulin-like [Maylandia zebra]
eeel_rep_c55919	+	2.089	0.000	213514682	Musculoskeletal embryonic nuclear protein 1 [Salmo salar]
eeel_rep_c56122	+	1.817	0.009	410896760	PREDICTED: trafficking protein particle complex subunit 2-like [Takifugu rubripes]
eeel_rep_c56216	+	1.583	0.006		
eeel_rep_c56590	+	2.208	0.009	318056250	triosephosphate isomerase b [Ictalurus punctatus]
eeel_rep_c56727	+	1.529	0.024	499031856	PREDICTED: microsomal glutathione S-transferase 3-like [Maylandia zebra]
eeel_rep_c56890	+	1.790	0.006	499017111	PREDICTED: calmodulin-like [Maylandia zebra]
eeel_rep_c56917	+	2.084	0.006	432864846	PREDICTED: glutathione S-transferase Mu 3-like [Oryzias latipes]
eeel_rep_c56979	+	1.716	0.014	18858295	Na+/K+ -ATPase alpha 1 subunit [Danio rerio]
eeel_rep_c57242	+	1.727	0.014	499017111	PREDICTED: calmodulin-like [Maylandia zebra]
eeel_rep_c57375	+	1.598	0.009	189535522	PREDICTED: hepatocyte cell adhesion molecule-like [Danio rerio]
eeel_rep_c57503	+	1.868	0.000	185136366	cystatin precursor [Oncorhynchus mykiss]
eeel_rep_c57531	+	1.896	0.013	499017111	PREDICTED: calmodulin-like [Maylandia zebra]
eeel_rep_c57538	+	1.977	0.026	432925902	PREDICTED: cytolysin RTX-A-like [Oryzias latipes]
eeel_rep_c57696	+	1.634	0.007	498930983	PREDICTED: lactoylglutathione lyase-like [Maylandia zebra]
eeel_rep_c58096	+	1.625	0.016	348506662	PREDICTED: 28S ribosomal protein S18a mitochondrial-like [Oreochromis niloticus]
eeel_rep_c58199	+	1.559	0.009		
eeel_rep_c58510	+	2.247	0.009	318037359	musculoskeletal embryonic nuclear protein 1 [Ictalurus punctatus]
eeel_rep_c58971	+	1.768	0.017		
eeel_rep_c59192	+	1.520	0.047	20373137	G2/mitotic-specific cyclin-B1 [Danio rerio]
eeel_rep_c59205	+	1.754	0.011	189525232	PREDICTED: probable glutamate receptor-like [Danio rerio]
eeel_rep_c59297	+	1.697	0.024	41152375	ATP synthase subunit gamma mitochondrial [Danio rerio]
eeel_rep_c59459	+	1.719	0.026	348515731	PREDICTED: tubulin alpha-1C chain-like [Oreochromis niloticus]
eeel_rep_c59490	+	1.727	0.008	70778734	ATPase Na+/K+ transporting alpha 3b polypeptide [Danio rerio]
eeel_rep_c59601	+	8.077	0.011	41055570	parvalbumin isoform 1b [Danio rerio]
eeel_rep_c59645	+	1.665	0.030	41054601	voltage-dependent anion-selective channel protein 2 [Danio rerio]
eeel_rep_c59907	+	1.797	0.007	318101977	signal peptidase complex subunit 1 [Ictalurus punctatus]

eeel_rep_c59912	+	1.610	0.016	226443324	S100 calcium binding protein beta (neural) [Salmo salar]
eeel_rep_c60287	+	3.075	0.036	70912382	uncharacterized protein LOC368621 precursor [Danio rerio]
eeel_rep_c60716	+	1.606	0.013	218751897	glutathione peroxidase 4 precursor [Danio rerio]
eeel_rep_c60760	+	1.935	0.014	410958666	PREDICTED: tubulin beta chain isoform 2 [Felis catus]
eeel_rep_c60983	+	5.247	0.030		
eeel_rep_c61254	+	5.546	0.005	41055570	parvalbumin isoform 1b [Danio rerio]
eeel_s59840	+	1.535	0.024	238231795	ADP-ribosylation factor 4 [Oncorhynchus mykiss]
eeel_s60038	+	1.626	0.006		
eeel2_c1025	+	2.299	0.008	373838782	protachykinin-1 precursor [Danio rerio]
eeel2_c105	+	2.509	0.007	185135286	collagen alpha-2(I) chain precursor [Oncorhynchus mykiss]
eeel2_c1054	+	1.541	0.039	338723081	PREDICTED: dynein light chain roadblock-type 2-like [Equus caballus]
eeel2_c1057	+	4.573	0.000	348502427	PREDICTED: neuralized-like protein 2-like [Oreochromis niloticus]
eeel2_c1110	+	1.549	0.008	126283549	PREDICTED: proteasome subunit alpha type-6-like [Monodelphis domestica]
eeel2_c1112	+	2.673	0.026	373838776	tachykinin 3a precursor [Danio rerio]
eeel2_c1118	+	1.680	0.011		
eeel2_c113	+	1.658	0.012	499035754	PREDICTED: isopentenyl-diphosphate Delta-isomerase 1-like isoform X1 [Maylandia zebra]
eeel2_c1136	+	1.514	0.021	213512266	Pyridoxal phosphate phosphatase PHOSPHO2 [Salmo salar]
eeel2_c1160	+	1.625	0.009	348519641	PREDICTED: NADH-ubiquinone oxidoreductase 75 kDa subunit mitochondrial-like isoform 1 [Oreochromis niloticus]
eeel2_c1192	+	1.896	0.014	213511846	Four and a half LIM domains protein 1 [Salmo salar]
eeel2_c1213	+	4.192	0.006	255069762	peptidyl-prolyl cis-trans isomerase FKBP10 [Danio rerio]
eeel2_c1215	+	2.127	0.026	224809395	collagen alpha-2(V) chain precursor [Danio rerio]
eeel2_c124	+	5.133	0.026	56790315	collagen alpha-1(I) chain precursor [Danio rerio]
eeel2_c126	+	1.706	0.005	213512117	ATP synthase H+ transporting mitochondrial F0 complex subunit c-3 [Salmo salar]
eeel2_c1261	+	1.960	0.013	213514570	26S proteasome non-ATPase regulatory subunit 12 [Salmo salar]
eeel2_c1294	+	1.545	0.026	348513450	PREDICTED: vigilin-like [Oreochromis niloticus]
eeel2_c130	+	3.221	0.000	348540479	PREDICTED: SLAM family member 7-like [Oreochromis niloticus]
eeel2_c1301	+	1.680	0.013	213510972	neuropeptide-like protein C4orf48 homolog precursor [Salmo salar]
eeel2_c1333	+	1.723	0.008	18858295	Na+/K+ -ATPase alpha 1 subunit [Danio rerio]
eeel2_c1349	+	4.099	0.000	185132790	retinol-binding protein 2 precursor [Oncorhynchus mykiss]
eeel2_c1359	+	1.724	0.036	139949174	collagen alpha-1(X) chain precursor [Danio rerio]
eeel2_c1405	+	1.840	0.011	348521966	PREDICTED: far upstream element-binding protein 1 [Oreochromis niloticus]
eeel2_c1410	+	3.868	0.039	348531926	PREDICTED: collagen alpha-1(I) chain-like [Oreochromis niloticus]
eeel2_c1433	+	1.657	0.024	47085997	glycerophosphodiester phosphodiesterase domain containing 1 like [Danio rerio]
eeel2_c1462	+	2.895	0.039	185135286	collagen alpha-2(I) chain precursor [Oncorhynchus mykiss]
eeel2_c1483	+	2.734	0.039	213514900	isoamyl acetate-hydrolyzing esterase 1 homolog [Salmo salar]
eeel2_c1488	+	1.723	0.013	410916855	PREDICTED: latisemin-like [Takifugu rubripes]
eeel2_c1508	+	1.563	0.026	50540440	flavin reductase [Danio rerio]

eeel2_c1510	+	1.519	0.030	348533822	PREDICTED: gamma-secretase subunit PEN-2-like [Oreochromis niloticus]
eeel2_c1550	+	2.513	0.024	498972905	PREDICTED: lactose-binding lectin l-2-like [Maylandia zebra]
eeel2_c1576	+	2.153	0.005	348514660	PREDICTED: alpha-enolase-like isoform 1 [Oreochromis niloticus]
eeel2_c158	+	1.503	0.007	41152171	mid1-interacting protein 1-B [Danio rerio]
eeel2_c1605	+	1.570	0.012	318055366	mitochondrial pyruvate dehydrogenase e1 component subunit beta [Ictalurus punctatus]
eeel2_c1629	+	4.409	0.007	499032299	PREDICTED: myelin basic protein-like isoform X1 [Maylandia zebra]
eeel2_c1684	+	2.473	0.011	238624212	natriuretic peptide precursor C-like protein precursor [Danio rerio]
eeel2_c1691	+	1.877	0.026	348513721	PREDICTED: sarcoplasmic/endoplasmic reticulum calcium ATPase 2-like isoform 1 [Oreochromis niloticus]
eeel2_c1714	+	1.555	0.047	499050727	PREDICTED: developmentally-regulated GTP-binding protein 2-like isoform X1 [Maylandia zebra]
eeel2_c1720	+	1.519	0.000		
eeel2_c176	+	1.622	0.009	327262669	PREDICTED: gem-associated protein 6-like [Anolis carolinensis]
eeel2_c186	+	2.292	0.021	348504600	PREDICTED: LOW QUALITY PROTEIN: type I iodothyronine deiodinase [Oreochromis niloticus]
eeel2_c1906	+	2.024	0.024	403279715	PREDICTED: dynein light chain 2 cytoplasmic [Saimiri boliviensis boliviensis]
eeel2_c1929	+	1.713	0.011	348524274	PREDICTED: hypothetical protein LOC100692451 [Oreochromis niloticus]
eeel2_c1952	+	3.751	0.039	348538615	PREDICTED: glutamate decarboxylase 1-like [Oreochromis niloticus]
eeel2_c1987	+	1.634	0.008	327262669	PREDICTED: gem-associated protein 6-like [Anolis carolinensis]
eeel2_c2017	+	8.221	0.000	499045677	PREDICTED: myelin proteolipid protein-like isoform X1 [Maylandia zebra]
eeel2_c2022	+	1.861	0.017	185132395	serotransferrin-1 precursor [Salmo salar]
eeel2_c2032	+	1.862	0.036	390355294	PREDICTED: uncharacterized protein LOC578303 [Strongylocentrotus purpuratus]
eeel2_c2060	+	1.557	0.030	287327510	selenoprotein X 1b [Danio rerio]
eeel2_c2061	+	3.930	0.039	326674760	PREDICTED: microfibrillar-associated protein 2-like [Danio rerio]
eeel2_c2073	+	1.592	0.006	432850599	PREDICTED: cathepsin D-like isoform 1 [Oryzias latipes]
eeel2_c2138	+	1.826	0.005	292624780	PREDICTED: neuroendocrine protein 7B2-like isoform 2 [Danio rerio]
eeel2_c2148	+	1.606	0.017	348539067	PREDICTED: NSFL1 cofactor p47-like [Oreochromis niloticus]
eeel2_c2161	+	1.639	0.008	348519148	PREDICTED: metallothionein-like [Oreochromis niloticus]
eeel2_c220	+	2.461	0.008	317575728	protachykinin-1 precursor [Ictalurus punctatus]
eeel2_c2257	+	1.800	0.030	348541249	PREDICTED: complement component 1 Q subcomponent-binding protein mitochondrial-like [Oreochromis niloticus]
eeel2_c2261	+	1.565	0.026	56693357	epoxide hydrolase 2 [Danio rerio]
eeel2_c2264	+	5.209	0.000	348538615	PREDICTED: glutamate decarboxylase 1-like [Oreochromis niloticus]
eeel2_c2387	+	1.501	0.039		
eeel2_c240	+	1.746	0.011	149773511	39S ribosomal protein L36 mitochondrial [Danio rerio]
eeel2_c2496	+	4.022	0.000	426228277	PREDICTED: dnaJ homolog subfamily B member 6-like [Ovis aries]
eeel2_c2578	+	3.094	0.007	318065049	cathepsin K precursor [Ictalurus punctatus]
eeel2_c2653	+	1.800	0.026	292617090	PREDICTED: hypothetical protein LOC321191 [Danio rerio]

eeel2_c2656	+	2.006	0.036	213514432	Neurogenic differentiation factor 1 [Salmo salar]
eeel2_c2659	+	2.769	0.021		
eeel2_c2659	+	1.865	0.047		
eeel2_c2699	+	1.537	0.011	320461693	vacuolar ATPase assembly integral membrane protein VMA21 [Danio rerio]
eeel2_c2704	+	1.780	0.021	156365963	predicted protein [Nematostella vectensis]
eeel2_c271	+	1.872	0.016		
eeel2_c2726	+	1.532	0.017	185132941	simple type II keratin K8a (S1) [Oncorhynchus mykiss]
eeel2_c2745	+	6.155	0.009	499026685	PREDICTED: G-protein coupled receptor 64-like [Maylandia zebra]
eeel2_c2783	+	2.463	0.026	348542034	PREDICTED: CD9 antigen-like [Oreochromis niloticus]
eeel2_c2799	+	1.820	0.009	410913901	PREDICTED: membrane-spanning 4-domains subfamily A member 4D-like [Takifugu rubripes]
eeel2_c2824	+	3.230	0.000	185132790	retinol-binding protein 2 precursor [Oncorhynchus mykiss]
eeel2_c2907	+	1.937	0.005	348528925	PREDICTED: aspartate aminotransferase cytoplasmic-like [Oreochromis niloticus]
eeel2_c2923	+	1.591	0.008	185136281	SPARC precursor [Oncorhynchus mykiss]
eeel2_c2968	+	2.051	0.036	318056044	s100-a10 [Ictalurus punctatus]
eeel2_c3014	+	1.775	0.013	432880273	PREDICTED: SH3 domain-binding glutamic acid-rich-like protein-like [Oryzias latipes]
eeel2_c308	+	1.790	0.012	499039976	PREDICTED: gelsolin-like [Maylandia zebra]
eeel2_c3095	+	1.611	0.000	41055291	ribosome maturation protein SBDS [Danio rerio]
eeel2_c3108	+	1.504	0.030	432849807	PREDICTED: alpha-16-mannosylglycoprotein 6-beta-N-acetylglucosaminyltransferase A-like [Oryzias latipes]
eeel2_c3120	+	2.178	0.039	348545902	PREDICTED: lactose-binding lectin l-2-like [Oreochromis niloticus]
eeel2_c318	+	1.726	0.014	348514215	PREDICTED: elongation factor 1-gamma-like [Oreochromis niloticus]
eeel2_c3183	+	1.894	0.013	194332544	uncharacterized protein LOC100170510 [Xenopus (Silurana) tropicalis]
eeel2_c3184	+	1.669	0.036	71834572	sorting nexin-6 [Danio rerio]
eeel2_c326	+	1.657	0.013	348544773	PREDICTED: tetraspanin-13-like [Oreochromis niloticus]
eeel2_c3272	+	1.566	0.047	51010953	uncharacterized protein LOC445037 [Danio rerio]
eeel2_c3294	+	1.603	0.005	348509635	PREDICTED: vacuolar protein sorting-associated protein 26A-like [Oreochromis niloticus]
eeel2_c3357	+	1.698	0.014	390461319	PREDICTED: LOW QUALITY PROTEIN: tubulin beta-2B chain [Callithrix jacchus]
eeel2_c3358	+	1.956	0.021	259155204	StAR-related lipid transfer protein 5 [Salmo salar]
eeel2_c3391	+	2.279	0.007	45361327	integral membrane protein 2B [Xenopus (Silurana) tropicalis]
eeel2_c3431	+	1.527	0.011		
eeel2_c3458	+	1.731	0.036	313851114	anterior gradient protein 3 homolog precursor [Gallus gallus]
eeel2_c347	+	1.744	0.005	348540060	PREDICTED: transmembrane BAX inhibitor motif-containing protein 1-like [Oreochromis niloticus]
eeel2_c3496	+	2.194	0.008	145495023	hypothetical protein [Paramecium tetraurelia strain d4-2]
eeel2_c3531	+	1.782	0.006	33504535	metalloproteinase inhibitor 2 precursor [Danio rerio]
eeel2_c3555	+	1.569	0.013	348500699	PREDICTED: ATP-binding cassette sub-family B member 8 mitochondrial-like [Oreochromis niloticus]

eeel2_c3583	+	1.818	0.036	488569054	PREDICTED: vegetative cell wall protein gp1-like partial [Dasypus novemcinctus]
eeel2_c3635	+	2.442	0.013	348516473	PREDICTED: thioredoxin-like protein 4B-like [Oreochromis niloticus]
eeel2_c3659	+	1.704	0.008	432952290	PREDICTED: uncharacterized protein LOC101163787 [Oryzias latipes]
eeel2_c3667	+	2.668	0.005	410918077	PREDICTED: SH3 domain-containing protein 19-like [Takifugu rubripes]
eeel2_c3707	+	1.511	0.017		
eeel2_c376	+	10.757	0.007		
eeel2_c3760	+	2.270	0.047	348515339	PREDICTED: cytochrome b-245 heavy chain-like [Oreochromis niloticus]
eeel2_c3772	+	1.511	0.008	498959136	PREDICTED: cyclin-dependent kinase 4-like isoform X2 [Maylandia zebra]
eeel2_c3805	+	1.837	0.007	213514590	Very long-chain acyl-CoA synthetase [Salmo salar]
eeel2_c3809	+	6.321	0.014	156119437	homeobox protein engrailed-2-A [Xenopus laevis]
eeel2_c3822	+	1.959	0.012	407264591	PREDICTED: zinc finger protein 845 isoform 5 [Mus musculus]
eeel2_c3862	+	6.354	0.013		
eeel2_c3873	+	1.612	0.008	213513189	Ferritin lower subunit [Salmo salar]
eeel2_c3892	+	1.616	0.013	213512278	NFU1 iron-sulfur cluster scaffold homolog mitochondrial [Salmo salar]
eeel2_c3934	+	1.903	0.007	54400516	uncharacterized protein LOC449839 precursor [Danio rerio]
eeel2_c394	+	1.790	0.026	45383626	zinc finger protein ZIC 1 [Gallus gallus]
eeel2_c3970	+	1.621	0.013	225543532	transmembrane protein 35 [Danio rerio]
eeel2_c4011	+	1.619	0.009		
eeel2_c4039	+	1.813	0.008		
eeel2_c4052	+	1.724	0.012	297275735	PREDICTED: hypothetical protein LOC100426888 [Macaca mulatta]
eeel2_c4125	+	1.710	0.021		
eeel2_c417	+	1.561	0.014	498939662	PREDICTED: transmembrane emp24 domain-containing protein 10-like [Maylandia zebra]
eeel2_c419	+	1.567	0.013	348523467	PREDICTED: eukaryotic translation initiation factor 4 gamma 2-like [Oreochromis niloticus]
eeel2_c42	+	1.618	0.014	51011125	AN1-type zinc finger protein 1 [Danio rerio]
eeel2_c4289	+	4.481	0.039	167527299	hypothetical protein [Monosiga brevicollis MX1]
eeel2_c429	+	1.505	0.009	395529086	PREDICTED: complement component 1 Q subcomponent-binding protein mitochondrial partial [Sarcophilus harrisii]
eeel2_c4290	+	1.532	0.039		
eeel2_c451	+	2.441	0.011	126334578	PREDICTED: myosin-7-like [Monodelphis domestica]
eeel2_c4586	+	1.799	0.006	348531160	PREDICTED: uncharacterized protein C15orf57 homolog [Oreochromis niloticus]
eeel2_c461	+	2.008	0.000	410927292	PREDICTED: uncharacterized protein LOC101076001 [Takifugu rubripes]
eeel2_c4675	+	3.146	0.021	478512080	PREDICTED: transcription factor AP-2 gamma [Ceratotherium simum simum]
eeel2_c4714	+	2.377	0.007	499018576	PREDICTED: NIPA-like protein 2-like isoform X1 [Maylandia zebra]
eeel2_c4717	+	2.193	0.008	292618214	PREDICTED: periostin [Danio rerio]
eeel2_c4836	+	1.857	0.011	410930125	PREDICTED: solute carrier organic anion transporter family member 2A1-like [Takifugu rubripes]
eeel2_c4856	+	2.952	0.000		

eeel2_c509	+	9.847	0.000	209954618	type III iodothyronine deiodinase b [Takifugu rubripes]
eeel2_c511	+	1.656	0.024	47085927	actin-related protein 2/3 complex subunit 1B [Danio rerio]
eeel2_c513	+	1.552	0.011	213510740	MKI67 FHA domain-interacting nucleolar phosphoprotein-like [Salmo salar]
eeel2_c523	+	2.528	0.006	317574783	prodynorphin precursor [Ictalurus punctatus]
eeel2_c529	+	3.982	0.024	20373145	claudin b [Danio rerio]
eeel2_c547	+	1.739	0.014	410920475	PREDICTED: DNA-binding protein inhibitor ID-1-like [Takifugu rubripes]
eeel2_c559	+	1.527	0.039	226442761	ORM1-like protein 1 [Salmo salar]
eeel2_c5618	+	1.585	0.036		
eeel2_c562	+	1.804	0.006	499028353	PREDICTED: CD9 antigen-like isoform X2 [Maylandia zebra]
eeel2_c565	+	1.653	0.014	292627305	PREDICTED: profilin-1 isoform 1 [Danio rerio]
eeel2_c597	+	2.428	0.016	410920475	PREDICTED: DNA-binding protein inhibitor ID-1-like [Takifugu rubripes]
eeel2_c6	+	3.741	0.021	348533606	PREDICTED: troponin C slow skeletal and cardiac muscles-like [Oreochromis niloticus]
eeel2_c600	+	1.569	0.005		
eeel2_c62	+	1.636	0.047	238231795	ADP-ribosylation factor 4 [Oncorhynchus mykiss]
eeel2_c622	+	2.558	0.012	319738633	myoferlin [Danio rerio]
eeel2_c65	+	2.216	0.008	55925187	TP53RK-binding protein [Danio rerio]
eeel2_c679	+	1.669	0.005	346644752	ADP-ribosylation factor-like protein 6-interacting protein 1 [Sus scrofa]
eeel2_c683	+	1.753	0.036	260805718	hypothetical protein BRAFLDRAFT_217335 [Branchiostoma floridae]
eeel2_c703	+	2.209	0.016	432861686	PREDICTED: tropomyosin alpha-1 chain-like isoform 1 [Oryzias latipes]
eeel2_c730	+	1.520	0.008	41054860	probable RNA-binding protein EIF1AD [Danio rerio]
eeel2_c739	+	1.572	0.026	213511408	CDKN2AIP N-terminal-like protein [Salmo salar]
eeel2_c767	+	1.612	0.011	327272564	PREDICTED: NADH dehydrogenase [ubiquinone] 1 alpha subcomplex subunit 6-like [Anolis carolinensis]
eeel2_c78	+	3.510	0.006	432939948	PREDICTED: LOW QUALITY PROTEIN: glutathione peroxidase 2-like [Oryzias latipes]
eeel2_c784	+	1.786	0.008	185134464	peroxiredoxin [Oncorhynchus mykiss]
eeel2_c807	+	1.606	0.014	62955283	mRNA turnover protein 4 homolog [Danio rerio]
eeel2_c819	+	2.926	0.017	291190538	Extracellular matrix protein 1 precursor [Salmo salar]
eeel2_c83	+	1.514	0.024	318103615	brain protein 44-like protein [Ictalurus punctatus]
eeel2_c832	+	7.862	0.026	125812522	PREDICTED: hypothetical protein LOC798209 [Danio rerio]
eeel2_c870	+	1.540	0.016	226443324	S100 calcium binding protein beta (neural) [Salmo salar]
eeel2_c928	+	1.618	0.012	238231687	protein RER1 [Oncorhynchus mykiss]
eeel2_c936	+	1.543	0.009	318056250	triosephosphate isomerase b [Ictalurus punctatus]
eeel2_c94	+	1.956	0.047	171544947	collagen type I alpha 1 precursor [Oryzias latipes]
eeel2_c978	+	1.585	0.012	51468069	proteasome subunit beta type-1 [Danio rerio]
eeel2_c994	+	1.769	0.021	30410758	keratin type I cytoskeletal 18 [Danio rerio]
eeel2_c998	+	1.914	0.011	348533013	PREDICTED: nucleolar protein 16-like [Oreochromis niloticus]
eeel2_rep_c4330	+	1.624	0.013	432921572	PREDICTED: NHP2-like protein 1-like [Oryzias latipes]
eeel2_rep_c4339	+	1.556	0.036	432864846	PREDICTED: glutathione S-transferase Mu 3-like [Oryzias latipes]

eeel2_rep_c4381	+	1.514	0.012	348531830	PREDICTED: UPF0587 protein C1orf123 homolog [Oreochromis niloticus]
eeel2_rep_c4405	+	1.645	0.006	213512117	ATP synthase H+ transporting mitochondrial F0 complex subunit c-3 [Salmo salar]
eeel2_rep_c4454	+	1.590	0.016	499031856	PREDICTED: microsomal glutathione S-transferase 3-like [Maylandia zebra]
eeel2_rep_c4503	+	1.751	0.007	499017111	PREDICTED: calmodulin-like [Maylandia zebra]
eeel2_rep_c4639	+	1.542	0.008	213511768	Ribonuclease H2 subunit C [Salmo salar]
eeel2_rep_c4716	+	1.540	0.012	238231795	ADP-ribosylation factor 4 [Oncorhynchus mykiss]
eeel2_rep_c4905	+	1.603	0.000	410928504	PREDICTED: sodium/potassium-transporting ATPase subunit beta-233-like [Takifugu rubripes]
eeel2_rep_c4906	+	1.647	0.005	213514924	nucleobindin-1 precursor [Salmo salar]
eeel2_rep_c4929	+	1.518	0.008	47085917	lactoylglutathione lyase [Danio rerio]
eeel2_rep_c4942	+	1.517	0.024	326668491	PREDICTED: uncharacterized protein C7orf57 homolog isoform 2 [Danio rerio]
eeel2_rep_c4943	+	1.656	0.017	498942894	PREDICTED: angiopoietin-related protein 4-like [Maylandia zebra]
eeel2_rep_c4956	+	1.556	0.009	291407865	PREDICTED: ubiquitin-conjugating enzyme E2A-like isoform 2 [Oryctolagus cuniculus]
eeel2_rep_c4972	+	1.694	0.026	348520800	PREDICTED: retinal rod rhodopsin-sensitive cGMP 3'5'-cyclic phosphodiesterase subunit gamma-like [Oreochromis niloticus]
eeel2_rep_c4988	+	1.868	0.009	62955529	cathepsin K precursor [Danio rerio]
eeel2_rep_c4999	+	1.609	0.021	348511249	PREDICTED: DNA-directed RNA polymerases I II and III subunit RPABC2-like [Oreochromis niloticus]
eeel2_rep_c5002	+	2.119	0.006	410925248	PREDICTED: tetraspanin-9-like [Takifugu rubripes]
eeel2_rep_c5016	+	2.157	0.016		
eeel2_rep_c5052	+	1.529	0.006	41055291	ribosome maturation protein SBDS [Danio rerio]
eeel2_rep_c5126	+	1.657	0.011	213514916	CA123 protein [Salmo salar]
eeel2_rep_c5133	+	1.602	0.006	47777306	voltage-dependent anion-selective channel protein 1 [Danio rerio]
eeel2_rep_c5183	+	4.462	0.021	185132790	retinol-binding protein 2 precursor [Oncorhynchus mykiss]
eeel2_rep_c5194	+	1.821	0.021	348512753	PREDICTED: very long-chain acyl-CoA synthetase-like [Oreochromis niloticus]
eeel2_rep_c5203	+	1.818	0.006	410917906	PREDICTED: uncharacterized protein LOC101078106 [Takifugu rubripes]
eeel2_rep_c5223	+	1.851	0.011	498997406	PREDICTED: TP53RK-binding protein-like isoform X1 [Maylandia zebra]
eeel2_rep_c5236	+	2.093	0.009	213514682	Musculoskeletal embryonic nuclear protein 1 [Salmo salar]
eeel2_rep_c5279	+	2.000	0.009	410928504	PREDICTED: sodium/potassium-transporting ATPase subunit beta-233-like [Takifugu rubripes]
eeel2_rep_c5289	+	1.564	0.009	45361541	secreted protein acidic cysteine-rich precursor [Xenopus (Silurana) tropicalis]
eeel2_rep_c5326	+	1.934	0.012	410916855	PREDICTED: latisemin-like [Takifugu rubripes]
eeel2_rep_c5392	+	6.943	0.013	41055570	parvalbumin isoform 1b [Danio rerio]
eeel2_rep_c5398	+	1.612	0.024	432864846	PREDICTED: glutathione S-transferase Mu 3-like [Oryzias latipes]
eeel2_rep_c5482	+	1.515	0.005	432921572	PREDICTED: NHP2-like protein 1-like [Oryzias latipes]
eeel2_rep_c5512	+	2.128	0.009	348503506	PREDICTED: hypothetical protein LOC100693404 [Oreochromis niloticus]
eeel2_rep_c5534	+	1.856	0.013	189525232	PREDICTED: probable glutamate receptor-like [Danio rerio]
eeel2_rep_c5535	+	2.025	0.008	327290016	PREDICTED: protein S100-A11-like [Anolis carolinensis]
eeel2_rep_c5634	+	1.741	0.017	356640249	voltage-dependent anion-selective channel protein 2 [Salmo salar]

eeel2_rep_c5670	+	2.075	0.008	348503506	PREDICTED: hypothetical protein LOC100693404 [Oreochromis niloticus]
eeel2_rep_c5737	+	1.631	0.000	348519148	PREDICTED: metallothionein-like [Oreochromis niloticus]
eeel2_rep_c5740	+	1.734	0.000	185136366	cystatin precursor [Oncorhynchus mykiss]
eeel2_rep_c5753	+	5.228	0.026	348545695	PREDICTED: lactose-binding lectin l-2-like [Oreochromis niloticus]
eeel2_rep_c5845	+	1.566	0.039	51010953	uncharacterized protein LOC445037 [Danio rerio]
eeel2_rep_c5849	+	1.880	0.012	499028554	PREDICTED: V-type proton ATPase subunit F-like [Maylandia zebra]
eeel2_rep_c5930	+	4.268	0.016	125851982	PREDICTED: troponin C skeletal muscle [Danio rerio]
eeel2_rep_c6182	+	1.944	0.000	185136366	cystatin precursor [Oncorhynchus mykiss]
eeel2_rep_c6192	+	3.859	0.005	499000985	PREDICTED: neurofilament light polypeptide-like [Maylandia zebra]
eeel2_rep_c6196	+	1.961	0.007	432864846	PREDICTED: glutathione S-transferase Mu 3-like [Oryzias latipes]
eeel2_rep_c6229	+	2.247	0.005	348543931	PREDICTED: tetraspanin-9-like [Oreochromis niloticus]
eeel2_rep_c6286	+	1.635	0.009	45383337	SPARC precursor [Gallus gallus]
eeel2_rep_c6521	+	1.814	0.000		
eeel2_rep_c6749	+	1.584	0.007	348519148	PREDICTED: metallothionein-like [Oreochromis niloticus]
eeel2_rep_c7005	+	2.037	0.024	348514668	PREDICTED: ATP synthase subunit b mitochondrial-like [Oreochromis niloticus]
eeel2_rep_c8024	+	1.699	0.000	185136366	cystatin precursor [Oncorhynchus mykiss]
eeel2_rep_c8130	+	1.566	0.000	41055656	programmed cell death protein 6 [Danio rerio]
eeel2_rep_c8221	+	1.791	0.000	41055656	programmed cell death protein 6 [Danio rerio]
eeel2_rep_c8242	+	1.598	0.012	213513189	Ferritin lower subunit [Salmo salar]
eeel2_rep_c8295	+	1.507	0.026	47085917	lactoylglutathione lyase [Danio rerio]
eeel2_rep_c8335	+	2.030	0.009	213514682	Musculoskeletal embryonic nuclear protein 1 [Salmo salar]
eeel2_rep_c8513	+	1.522	0.009	498930983	PREDICTED: lactoylglutathione lyase-like [Maylandia zebra]
eeel2_rep_c8725	+	1.723	0.008		
eeel2_rep_c8734	+	1.896	0.017	61806494	uncharacterized protein LOC497166 [Danio rerio]
eeel2_rep_c8768	+	1.588	0.007	45383337	SPARC precursor [Gallus gallus]
eeel2_rep_c9074	+	1.534	0.036	507647379	PREDICTED: general transcription factor IIH subunit 5-like [Echinops telfairi]
eeel2_rep_c9269	+	1.530	0.016	499006603	PREDICTED: aminopeptidase N-like [Maylandia zebra]
eeel2_s4944	+	1.694	0.007	41282137	ATPase Na+/K+ transporting alpha 3a polypeptide [Danio rerio]
eeel2_s4952	+	1.618	0.030	218751897	glutathione peroxidase 4 precursor [Danio rerio]
eeel2_s5067	+	1.502	0.017	213512551	Transmembrane protein 128 [Salmo salar]
eeel2_s5082	+	2.002	0.008	62955529	cathepsin K precursor [Danio rerio]
eeel2_s5139	+	2.590	0.012		
eeel2_s5197	+	1.963	0.007	185134464	peroxiredoxin [Oncorhynchus mykiss]
eeel2_s5252	+	1.581	0.014	136256410	protein Z-dependent protease inhibitor precursor [Danio rerio]
eeel2_s5288	+	1.784	0.007	185134464	peroxiredoxin [Oncorhynchus mykiss]
eeel2_s5352	+	1.827	0.012	213515560	succinate dehydrogenase [ubiquinone] iron-sulfur subunit mitochondrial [Salmo salar]
eeel2_s5407	+	1.683	0.017	213514192	AP-3 complex subunit sigma-1 [Salmo salar]
eeel2_s5412	+	1.646	0.021	218751897	glutathione peroxidase 4 precursor [Danio rerio]

eeel2_s5437	+	2.054	0.000		
eeel2_s5799	+	1.995	0.013	348503506	PREDICTED: hypothetical protein LOC100693404 [Oreochromis niloticus]
eeel2_s5892	+	1.676	0.036	61806494	uncharacterized protein LOC497166 [Danio rerio]
eeel2_s5947	+	1.511	0.008	47085917	lactoylglutathione lyase [Danio rerio]
eeel2_s6048	+	2.159	0.009	348503502	PREDICTED: hypothetical protein LOC100692858 [Oreochromis niloticus]
eeel2_s6061	+	1.731	0.014	189525232	PREDICTED: probable glutamate receptor-like [Danio rerio]
eeel2_s6114	+	1.677	0.039	61806494	uncharacterized protein LOC497166 [Danio rerio]
eeel2_s6127	+	1.553	0.016	226443324	S100 calcium binding protein beta (neural) [Salmo salar]
eeel2_s6190	+	1.553	0.016	213510766	General transcription factor IIH subunit 5 [Salmo salar]
eeel2_s6206	+	5.855	0.014	41055570	parvalbumin isoform 1b [Danio rerio]
eeel2_s6222	+	2.183	0.008	348503502	PREDICTED: hypothetical protein LOC100692858 [Oreochromis niloticus]
eeel2_s6224	+	1.643	0.036	61556716	ba1 globin like [Danio rerio]
eeel2_s6232	+	1.588	0.047	61806494	uncharacterized protein LOC497166 [Danio rerio]
eeel2_s6301	+	2.062	0.000		
eeel2_s6323	+	1.662	0.000	213512117	ATP synthase H+ transporting mitochondrial F0 complex subunit c-3 [Salmo salar]
eeel2_s6325	+	1.743	0.026	61806494	uncharacterized protein LOC497166 [Danio rerio]
eeel2_s6357	+	1.704	0.030	61806494	uncharacterized protein LOC497166 [Danio rerio]
eeel2_s6841	+	1.677	0.011	70778734	ATPase Na+/K+ transporting alpha 3b polypeptide [Danio rerio]
eeel2_s6864	+	1.927	0.017	61806494	uncharacterized protein LOC497166 [Danio rerio]
eeel2_s7021	+	2.008	0.021	499003779	PREDICTED: tubulin alpha chain-like isoform X1 [Maylandia zebra]
eeel2_s8002	+	1.642	0.024	218751897	glutathione peroxidase 4 precursor [Danio rerio]
eeel2_s8023	+	1.608	0.014	348538208	PREDICTED: hypothetical protein LOC100700323 [Oreochromis niloticus]
eeel2_s8120	+	5.021	0.013	348545695	PREDICTED: lactose-binding lectin l-2-like [Oreochromis niloticus]
eeel2_s8135	+	2.201	0.000	213514682	Musculoskeletal embryonic nuclear protein 1 [Salmo salar]
eeel2_s8151	+	1.606	0.024	504165112	PREDICTED: V-type proton ATPase subunit F [Ochotona princeps]
eeel2_s8170	+	1.989	0.008	47086689	glutathione S-transferase M [Danio rerio]
eeel2_s8203	+	1.590	0.006	213512117	ATP synthase H+ transporting mitochondrial F0 complex subunit c-3 [Salmo salar]
eeel2_s8240	+	3.097	0.047	213512927	matrix Gla protein precursor [Salmo salar]
eeel2_s8393	+	1.656	0.039	61806494	uncharacterized protein LOC497166 [Danio rerio]
eeel2_s8399	+	1.507	0.017	354479645	PREDICTED: syntenin-1-like [Cricetulus griseus]
eeel2_s8482	+	1.518	0.036	51467909	ATP synthase subunit O mitochondrial [Danio rerio]
eeel2_s8518	+	1.706	0.000	185136366	cystatin precursor [Oncorhynchus mykiss]
eeel2_s8571	+	2.183	0.007	410920806	PREDICTED: sodium/potassium-transporting ATPase subunit beta-233-like [Takifugu rubripes]
eeel2_s8635	+	2.277	0.011	410928504	PREDICTED: sodium/potassium-transporting ATPase subunit beta-233-like [Takifugu rubripes]
eeel2_s8675	+	2.250	0.012	41055570	parvalbumin isoform 1b [Danio rerio]
eeel2_s8678	+	1.869	0.000		
eeel2_s8678	+	1.733	0.017		

eeel2_s8687	+	7.233	0.024	348545695	PREDICTED: lactose-binding lectin l-2-like [Oreochromis niloticus]
eeel2_s8746	+	1.841	0.021	61806494	uncharacterized protein LOC497166 [Danio rerio]
eeel2_s8756	+	1.824	0.024	61806494	uncharacterized protein LOC497166 [Danio rerio]
eeel2_s8762	+	1.895	0.021	61806494	uncharacterized protein LOC497166 [Danio rerio]
eeel2_s8765	+	1.835	0.024	61806494	uncharacterized protein LOC497166 [Danio rerio]
eeel2_s8816	+	1.740	0.017	61556716	ba1 globin like [Danio rerio]
eeel2_s8851	+	1.803	0.007	70778734	ATPase Na+/K+ transporting alpha 3b polypeptide [Danio rerio]
eeel2_s8861	+	1.541	0.012	301610364	PREDICTED: tumor suppressor candidate 3 [Xenopus (Silurana) tropicalis]
eeel2_s8882	+	1.650	0.036	51010953	uncharacterized protein LOC445037 [Danio rerio]
eeel2_s8903	+	1.669	0.036	61806494	uncharacterized protein LOC497166 [Danio rerio]
eeel2_s8926	+	8.358	0.017	41055570	parvalbumin isoform 1b [Danio rerio]
eeel2_s9035	+	2.285	0.030	327290016	PREDICTED: protein S100-A11-like [Anolis carolinensis]
eeel2_s9081	+	2.647	0.047		
eeel2_s9131	+	1.664	0.026	61556716	ba1 globin like [Danio rerio]
eeel2_s9132	+	1.905	0.016	185132478	hemoglobin subunit alpha [Salmo salar]
eeel2_s9145	+	2.179	0.009	348503506	PREDICTED: hypothetical protein LOC100693404 [Oreochromis niloticus]
eeel2_s9200	+	10.510	0.013	41055570	parvalbumin isoform 1b [Danio rerio]
eeel2_s9221	+	1.717	0.036	341926186	general transcription factor IIH subunit 5 [Danio rerio]
eeel_c10040	-	0.585	0.039	395521641	PREDICTED: complement C1q subcomponent subunit C [Sarcophilus harrisii]
eeel_c10530	-	0.605	0.039	393715121	MHC class I alpha chain precursor [Oncorhynchus mykiss]
eeel_c10720	-	0.658	0.036	410917348	PREDICTED: serine/threonine-protein kinase N2-like [Takifugu rubripes]
eeel_c1109	-	0.471	0.047		
eeel_c11189	-	0.612	0.039	432921184	PREDICTED: protein S100-P-like [Oryzias latipes]
eeel_c11280	-	0.628	0.012		
eeel_c11424	-	0.435	0.036	499045802	PREDICTED: nuclear factor 7 ovary-like [Maylandia zebra]
eeel_c11608	-	0.559	0.008	326676757	PREDICTED: hypothetical protein LOC557772 [Danio rerio]
eeel_c11630	-	0.613	0.014	312093606	hypothetical protein LOAG_12181 [Loa loa]
eeel_c11636	-	0.496	0.047	350539523	uncharacterized protein LOC100005232 [Danio rerio]
eeel_c11767	-	0.614	0.039	348531631	PREDICTED: DNA-binding protein inhibitor ID-2-like [Oreochromis niloticus]
eeel_c11816	-	0.541	0.012	410897104	PREDICTED: sacsin-like [Takifugu rubripes]
eeel_c11837	-	0.457	0.013	348521932	PREDICTED: nuclear factor 7 ovary-like [Oreochromis niloticus]
eeel_c11854	-	0.634	0.036		
eeel_c1213	-	0.514	0.047	185136252	heat shock 90kDa protein 1 beta isoform b [Oncorhynchus mykiss]
eeel_c12367	-	0.468	0.039	301632724	PREDICTED: placenta-specific gene 8 protein-like isoform X1 [Xenopus (Silurana) tropicalis]
eeel_c12641	-	0.278	0.021	326675398	PREDICTED: NACHT LRR and PYD domains-containing protein 1-like [Danio rerio]
eeel_c12642	-	0.377	0.039	213514902	pleckstrin [Salmo salar]
eeel_c12782	-	0.124	0.008	499017429	PREDICTED: viral T-cell receptor beta chain-like T17T-22-like isoform X2 [Maylandia zebra]

eeel_c13035	-	0.590	0.013	170060529	conserved hypothetical protein [Culex quinquefasciatus]
eeel_c13088	-	0.662	0.026	213514916	CA123 protein [Salmo salar]
eeel_c13140	-	0.608	0.013		
eeel_c13237	-	0.650	0.014		
eeel_c13375	-	0.452	0.030	45361531	proteasome (prosome macropain) 26S subunit ATPase 6 [Xenopus (Silurana) tropicalis]
eeel_c13616	-	0.452	0.047	498938413	PREDICTED: urokinase plasminogen activator surface receptor-like isoform X1 [Maylandia zebra]
eeel_c13769	-	0.515	0.030	345328502	PREDICTED: interferon-induced protein with tetratricopeptide repeats 5-like [Ornithorhynchus anatinus]
eeel_c13778	-	0.601	0.013	392356281	PREDICTED: heat shock protein HSP 90-beta-like [Rattus norvegicus]
eeel_c13876	-	0.599	0.036	390355294	PREDICTED: uncharacterized protein LOC578303 [Strongylocentrotus purpuratus]
eeel_c14062	-	0.533	0.014	348545266	PREDICTED: protein NLRC3-like partial [Oreochromis niloticus]
eeel_c14236	-	0.440	0.026		
eeel_c14355	-	0.587	0.017	83415096	protein SREK1IP1 [Danio rerio]
eeel_c14426	-	0.376	0.021	260809321	hypothetical protein BRAFLDRAFT_81045 [Branchiostoma floridae]
eeel_c14487	-	0.646	0.021	62858585	nuclear apoptosis inducing factor 1 [Xenopus (Silurana) tropicalis]
eeel_c14563	-	0.491	0.024	18858561	homeobox protein Dlx4b [Danio rerio]
eeel_c14680	-	0.615	0.047	326918909	PREDICTED: YTH domain-containing protein 1-like isoform 1 [Meleagris gallopavo]
eeel_c14861	-	0.581	0.026	74095921	CD45 precursor [Takifugu rubripes]
eeel_c14921	-	0.643	0.036	348541039	PREDICTED: protein NLRC3-like [Oreochromis niloticus]
eeel_c14957	-	0.394	0.047	499029866	PREDICTED: protein NLRC3-like [Maylandia zebra]
eeel_c14987	-	0.651	0.039	224004208	calmodulin [Thalassiosira pseudonana CCMP1335]
eeel_c15210	-	0.491	0.036	148223093	diamine N-acetyltransferase 1 a [Xenopus laevis]
eeel_c1543	-	0.485	0.047	41056159	tumor necrosis factor alpha-induced protein 8-like protein 1 [Danio rerio]
eeel_c1577	-	0.475	0.008	118354437	hypothetical protein TTHERM_00355830 [Tetrahymena thermophila]
eeel_c16216	-	0.507	0.026	59276034	rho-related GTP-binding protein RhoV [Danio rerio]
eeel_c16424	-	0.637	0.039	213514964	prodynorphin precursor [Salmo salar]
eeel_c16565	-	0.483	0.030	390360486	PREDICTED: putative nuclease HARBI1-like [Strongylocentrotus purpuratus]
eeel_c16581	-	0.645	0.017	125837366	PREDICTED: ephexin-1-like [Danio rerio]
eeel_c16731	-	0.483	0.047	449514259	PREDICTED: interleukin-7 receptor subunit alpha [Taeniopygia guttata]
eeel_c16923	-	0.661	0.039	301770165	PREDICTED: kinesin-1 heavy chain-like [Ailuropoda melanoleuca]
eeel_c1759	-	0.583	0.012	348541751	PREDICTED: zinc finger C3H1 domain-containing protein-like [Oreochromis niloticus]
eeel_c17627	-	0.193	0.016	395502360	PREDICTED: protein KIAA1199 homolog [Sarcophilus harrisii]
eeel_c17663	-	0.644	0.026	348542909	PREDICTED: probable G-protein coupled receptor 148-like [Oreochromis niloticus]
eeel_c17672	-	0.639	0.016	348542497	PREDICTED: potassium voltage-gated channel subfamily C member 2-like [Oreochromis niloticus]
eeel_c17845	-	0.645	0.047		
eeel_c1787	-	0.359	0.039	504145946	PREDICTED: transcription factor PU.1 [Ochotona princeps]

eeel_c18065	-	0.618	0.014	326673134	PREDICTED: hypothetical protein LOC795947 [Danio rerio]
eeel_c18118	-	0.575	0.008	499002811	PREDICTED: ankyrin repeat domain-containing protein 12-like isoform X1 [Maylandia zebra]
eeel_c1812	-	0.576	0.016	348521634	PREDICTED: RNA-binding protein 10-like [Oreochromis niloticus]
eeel_c18232	-	0.565	0.011	432964404	PREDICTED: V-set and transmembrane domain-containing protein 2A-like [Oryzias latipes]
eeel_c18410	-	0.293	0.030	395510235	PREDICTED: myosin-4 [Sarcophilus harrisii]
eeel_c18528	-	0.562	0.036	395519341	PREDICTED: pre-mRNA 3' end processing protein WDR33 [Sarcophilus harrisii]
eeel_c18538	-	0.452	0.039	125806827	PREDICTED: peroxisomal proliferator-activated receptor A-interacting complex 285 kDa protein [Danio rerio]
eeel_c18595	-	0.618	0.013	326676024	PREDICTED: early endosome antigen 1 [Danio rerio]
eeel_c18685	-	0.586	0.036	348508310	PREDICTED: metabotropic glutamate receptor 5-like [Oreochromis niloticus]
eeel_c19154	-	0.598	0.008	498999940	PREDICTED: TATA-binding protein-associated factor 172-like [Maylandia zebra]
eeel_c19520	-	0.451	0.021	348524867	PREDICTED: structural maintenance of chromosomes protein 6-like [Oreochromis niloticus]
eeel_c1953	-	0.565	0.011	348507104	PREDICTED: actin-binding LIM protein 1 isoform 1 [Oreochromis niloticus]
eeel_c19560	-	0.461	0.047	498927034	PREDICTED: uncharacterized protein LOC101478254 [Maylandia zebra]
eeel_c19757	-	0.275	0.008	348519623	PREDICTED: hypothetical protein LOC100692986 [Oreochromis niloticus]
eeel_c19969	-	0.653	0.010	441614542	PREDICTED: unconventional myosin-XVI [Nomascus leucogenys]
eeel_c20006	-	0.581	0.008	190358411	glutamate [NMDA] receptor subunit epsilon-2 [Danio rerio]
eeel_c20176	-	0.359	0.017	301604768	PREDICTED: protein disulfide-isomerase-like protein of the testis-like [Xenopus (Silurana) tropicalis]
eeel_c20191	-	0.566	0.017	354490484	PREDICTED: ankyrin-3-like [Cricetulus griseus]
eeel_c20513	-	0.661	0.026		
eeel_c20713	-	0.550	0.047		
eeel_c20843	-	0.503	0.024	301624462	PREDICTED: interferon-induced very large GTPase 1-like [Xenopus (Silurana) tropicalis]
eeel_c20931	-	0.664	0.008	507671488	PREDICTED: caseinolytic peptidase B protein homolog isoform X1 [Echinops telfairi]
eeel_c21035	-	0.276	0.026		
eeel_c21293	-	0.523	0.011	326669328	PREDICTED: interferon-induced very large GTPase 1-like [Danio rerio]
eeel_c21303	-	0.580	0.013	326665392	PREDICTED: SURP and G-patch domain-containing protein 1 [Danio rerio]
eeel_c21398	-	0.573	0.011	499049991	PREDICTED: poly [ADP-ribose] polymerase 14-like [Maylandia zebra]
eeel_c21400	-	0.629	0.011	499025785	PREDICTED: sterile alpha motif domain-containing protein 9-like partial [Maylandia zebra]
eeel_c21723	-	0.620	0.008	410909422	PREDICTED: mitogen-activated protein kinase kinase kinase 15-like [Takifugu rubripes]
eeel_c21960	-	0.586	0.030	66821579	hypothetical protein DDB_G0274131 [Dictyostelium discoideum AX4]
eeel_c21978	-	0.626	0.008	167524286	hypothetical protein [Monosiga brevicollis MX1]
eeel_c22180	-	0.635	0.017	326676757	PREDICTED: hypothetical protein LOC557772 [Danio rerio]

eeel_c22407	-	0.499	0.024		
eeel_c22607	-	0.645	0.030	410897553	PREDICTED: LOW QUALITY PROTEIN: nebulin-like [Takifugu rubripes]
eeel_c22896	-	0.570	0.036	189515794	PREDICTED: chromodomain-helicase-DNA-binding protein 7 [Danio rerio]
eeel_c22987	-	0.654	0.047		
eeel_c23042	-	0.496	0.039	410929519	PREDICTED: piggyBac transposable element-derived protein 4-like partial [Takifugu rubripes]
eeel_c23100	-	0.586	0.008	410906923	PREDICTED: claudin-15-like [Takifugu rubripes]
eeel_c23132	-	0.585	0.021	301617343	PREDICTED: RNA-directed DNA polymerase from mobile element jockey-like [Xenopus (Silurana) tropicalis]
eeel_c23204	-	0.608	0.039	410899024	PREDICTED: uncharacterized protein LOC101068812 [Takifugu rubripes]
eeel_c23226	-	0.614	0.026		
eeel_c23432	-	0.288	0.014	392331971	PREDICTED: CMRF35-like molecule 3-like [Rattus norvegicus]
eeel_c23478	-	0.609	0.030		
eeel_c23554	-	0.530	0.039	113676668	uncharacterized protein LOC557909 [Danio rerio]
eeel_c23677	-	0.602	0.014	397492124	PREDICTED: uncharacterized protein LOC100991173 [Pan paniscus]
eeel_c23851	-	0.417	0.010	18858389	caspase a [Danio rerio]
eeel_c23908	-	0.560	0.008	348505657	PREDICTED: DNA damage-binding protein 2-like [Oreochromis niloticus]
eeel_c23940	-	0.659	0.039		
eeel_c23970	-	0.525	0.014		
eeel_c23976	-	0.627	0.030		
eeel_c23992	-	0.607	0.036		
eeel_c24008	-	0.579	0.047	348522064	PREDICTED: hypothetical protein LOC100707549 [Oreochromis niloticus]
eeel_c24048	-	0.641	0.013		
eeel_c24216	-	0.633	0.016	326676175	PREDICTED: voltage-gated potassium channel subunit beta-1-like [Danio rerio]
eeel_c24244	-	0.192	0.047	348510635	PREDICTED: immunoglobulin lambda-like polypeptide 1-like [Oreochromis niloticus]
eeel_c24359	-	0.530	0.030		
eeel_c24359	-	0.484	0.036		
eeel_c24447	-	0.445	0.012	259155232	Macrosialin precursor [Salmo salar]
eeel_c24555	-	0.325	0.047	348541141	PREDICTED: proteasome subunit beta type-6-B like protein-like isoform 1 [Oreochromis niloticus]
eeel_c24704	-	0.322	0.021	354475754	PREDICTED: PAX-interacting protein 1-like [Cricetulus griseus]
eeel_c24723	-	0.115	0.017	348564894	PREDICTED: CD209 antigen-like protein 2-like [Cavia porcellus]
eeel_c24725	-	0.628	0.030	259155244	G1/S-specific cyclin-D1 [Salmo salar]
eeel_c24786	-	0.558	0.021	498949527	PREDICTED: homer protein homolog 2-like isoform X1 [Maylandia zebra]
eeel_c24850	-	0.268	0.039	301620859	PREDICTED: prolyl endopeptidase-like partial [Xenopus (Silurana) tropicalis]
eeel_c25032	-	0.644	0.036	301606475	PREDICTED: AT-rich interactive domain-containing protein 2 isoform X1 [Xenopus (Silurana) tropicalis]
eeel_c25165	-	0.637	0.016	326676757	PREDICTED: hypothetical protein LOC557772 [Danio rerio]
eeel_c25201	-	0.395	0.036	499017216	PREDICTED: nuclear GTPase SLIP-GC-like [Maylandia zebra]

eeel_c25206	-	0.588	0.008	27545227	60S ribosomal protein L24 [Danio rerio]
eeel_c25216	-	0.503	0.030	326674455	PREDICTED: metabotropic glutamate receptor 5-like [Danio rerio]
eeel_c25265	-	0.573	0.036	348544530	PREDICTED: CD2-associated protein-like [Oreochromis niloticus]
eeel_c25385	-	0.561	0.024	52219190	cytochrome c oxidase subunit VIa-like [Danio rerio]
eeel_c25452	-	0.532	0.021	185136391	erythrocyte band 7 integral membrane protein precursor [Oncorhynchus mykiss]
eeel_c25582	-	0.354	0.024	45361585	creatine kinase mitochondrial 1B [Xenopus (Silurana) tropicalis]
eeel_c25614	-	0.634	0.017	115430075	finTRIM family member 82 [Danio rerio]
eeel_c25693	-	0.566	0.024		
eeel_c25794	-	0.634	0.014	348540607	PREDICTED: insulin receptor substrate 2-B-like [Oreochromis niloticus]
eeel_c25890	-	0.542	0.039		
eeel_c26017	-	0.609	0.047	91176308	CD8 antigen alpha polypeptide precursor [Danio rerio]
eeel_c26041	-	0.630	0.047	41055184	transformer-2 protein homolog beta [Danio rerio]
eeel_c26320	-	0.510	0.026	348541751	PREDICTED: zinc finger C3H1 domain-containing protein-like [Oreochromis niloticus]
eeel_c2635	-	0.597	0.024		
eeel_c26639	-	0.525	0.021	326671275	PREDICTED: hypothetical protein LOC100331563 [Danio rerio]
eeel_c26954	-	0.661	0.039	432882842	PREDICTED: DNA topoisomerase 2-beta-like [Oryzias latipes]
eeel_c26960	-	0.579	0.008	301171547	SAFB-like transcription modulator [Danio rerio]
eeel_c27063	-	0.538	0.024	410931036	PREDICTED: dynein heavy chain 9 axonemal-like [Takifugu rubripes]
eeel_c27167	-	0.582	0.011	348540353	PREDICTED: sorting nexin-19 [Oreochromis niloticus]
eeel_c27466	-	0.505	0.024	260791303	hypothetical protein BRAFLDRAFT_89480 [Branchiostoma floridae]
eeel_c27499	-	0.448	0.021	348541037	PREDICTED: protein NLRC3-like [Oreochromis niloticus]
eeel_c27528	-	0.641	0.016	395504470	PREDICTED: uncharacterized protein LOC100925129 [Sarcophilus harrisii]
eeel_c2759	-	0.607	0.017		
eeel_c2819	-	0.617	0.000	149773519	TM2 domain-containing protein 1 precursor [Danio rerio]
eeel_c28212	-	0.516	0.010	189536881	PREDICTED: cytochrome c oxidase subunit 4 isoform 2 mitochondrial [Danio rerio]
eeel_c2843	-	0.544	0.026	429484494	microtubule associated monoxygenase calponin and LIM domain containing 2b [Danio rerio]
eeel_c28450	-	0.660	0.047		
eeel_c28692	-	0.526	0.047	348501726	PREDICTED: hypothetical protein LOC100699980 [Oreochromis niloticus]
eeel_c28698	-	0.558	0.026	224809395	collagen alpha-2(V) chain precursor [Danio rerio]
eeel_c28800	-	0.509	0.047	348527870	PREDICTED: solute carrier family 22 member 5-like [Oreochromis niloticus]
eeel_c28820	-	0.636	0.030	471392200	PREDICTED: poly [ADP-ribose] polymerase 8 isoform 1 [Trichechus manatus latirostris]
eeel_c28968	-	0.441	0.024	189523699	PREDICTED: titin [Danio rerio]
eeel_c29070	-	0.422	0.047	348529132	PREDICTED: LOW QUALITY PROTEIN: PRKC apoptosis WT1 regulator protein-like [Oreochromis niloticus]
eeel_c29094	-	0.469	0.039	261245071	fish virus induced TRIM protein [Oncorhynchus mykiss]
eeel_c29173	-	0.646	0.017	326676757	PREDICTED: hypothetical protein LOC557772 [Danio rerio]
eeel_c29232	-	0.555	0.047	348542132	PREDICTED: protein FAM49A-like isoform 2 [Oreochromis niloticus]

eeel_c29555	-	0.544	0.039	348545840	PREDICTED: hypothetical protein LOC100706620 partial [Oreochromis niloticus]
eeel_c29653	-	0.599	0.024	308153271	U2-associated SR140 protein-like [Danio rerio]
eeel_c29718	-	0.654	0.039		
eeel_c29726	-	0.556	0.036	189537462	PREDICTED: ras association domain-containing protein 10 [Danio rerio]
eeel_c29782	-	0.623	0.039	291389862	PREDICTED: ribosomal protein L3-like [Oryctolagus cuniculus]
eeel_c30223	-	0.564	0.047	410931654	PREDICTED: GSK-3-binding protein-like [Takifugu rubripes]
eeel_c30237	-	0.655	0.008	334347705	PREDICTED: serine/threonine-protein phosphatase 6 regulatory subunit 2 [Monodelphis domestica]
eeel_c30420	-	0.536	0.024	432900014	PREDICTED: ribosomal protein S6 kinase beta-1-like [Oryzias latipes]
eeel_c30483	-	0.505	0.047	326665995	PREDICTED: interferon-induced 35 kDa protein homolog [Danio rerio]
eeel_c30532	-	0.577	0.016	68398601	PREDICTED: reticulocalbin-1 [Danio rerio]
eeel_c30617	-	0.640	0.030	498928809	PREDICTED: probable ATP-dependent RNA helicase DDX5-like isoform X1 [Maylandia zebra]
eeel_c30653	-	0.251	0.012		
eeel_c30703	-	0.565	0.039	326676905	PREDICTED: receptor-type tyrosine-protein phosphatase kappa [Danio rerio]
eeel_c31010	-	0.646	0.008	348545017	PREDICTED: tubulin-specific chaperone cofactor E-like protein-like [Oreochromis niloticus]
eeel_c3113	-	0.472	0.047	58332840	uncharacterized protein LOC496998 [Xenopus (Silurana) tropicalis]
eeel_c31576	-	0.658	0.021		
eeel_c31880	-	0.564	0.030	66472224	uncharacterized protein LOC553782 [Danio rerio]
eeel_c3194	-	0.507	0.021		
eeel_c32787	-	0.469	0.039	113681947	heparanase precursor [Danio rerio]
eeel_c32959	-	0.496	0.036	226533709	disintegrin and metalloproteinase domain-containing protein 10 precursor [Danio rerio]
eeel_c33073	-	0.616	0.047	317575863	pancreatic triacylglycerol lipase precursor [Ictalurus punctatus]
eeel_c33188	-	0.661	0.024	391331604	PREDICTED: 40S ribosomal protein S23-like [Metaseiulus occidentalis]
eeel_c33229	-	0.663	0.021	326667829	PREDICTED: transposon TX1 uncharacterized 149 kDa protein-like [Danio rerio]
eeel_c33583	-	0.600	0.047	176866341	endoplasmic reticulum aminopeptidase 2 precursor [Danio rerio]
eeel_c3374	-	0.585	0.011	47575780	peptidylprolyl isomerase G [Xenopus (Silurana) tropicalis]
eeel_c33792	-	0.503	0.026	291408584	PREDICTED: hypothetical protein [Oryctolagus cuniculus]
eeel_c33959	-	0.597	0.011	478530940	PREDICTED: protein ELYS [Ceratotherium simum simum]
eeel_c34113	-	0.577	0.030	499034626	PREDICTED: probable ubiquitin carboxyl-terminal hydrolase FAF-X-like isoform X1 [Maylandia zebra]
eeel_c34200	-	0.664	0.024	45361503	serine/arginine-rich splicing factor 2 [Xenopus (Silurana) tropicalis]
eeel_c34258	-	0.359	0.016	269914165	WD repeat-containing protein 37 [Danio rerio]
eeel_c34262	-	0.650	0.013	348530778	PREDICTED: hypothetical protein LOC100696644 [Oreochromis niloticus]
eeel_c34383	-	0.593	0.010	289191307	pinin desmosome associated protein [Danio rerio]
eeel_c34798	-	0.568	0.047	348537423	PREDICTED: apoptosis-inducing factor 1 mitochondrial-like [Oreochromis niloticus]
eeel_c34928	-	0.472	0.039	296192621	PREDICTED: probable tRNA(His) guanylyltransferase [Callithrix jacchus]

eeel_c35158	-	0.582	0.047	260782109	hypothetical protein BRAFLDRAFT_248491 [Branchiostoma floridae]
eeel_c35221	-	0.579	0.011	326665392	PREDICTED: SURP and G-patch domain-containing protein 1 [Danio rerio]
eeel_c35253	-	0.626	0.036	410913223	PREDICTED: zinc finger protein 280C-like [Takifugu rubripes]
eeel_c35353	-	0.614	0.047	291388018	PREDICTED: thymus high mobility group box protein TOX [Oryctolagus cuniculus]
eeel_c35401	-	0.244	0.047	292618696	PREDICTED: hypothetical protein LOC100332429 [Danio rerio]
eeel_c35409	-	0.590	0.014		
eeel_c35409	-	0.644	0.017		
eeel_c35418	-	0.601	0.039	348523363	PREDICTED: proto-oncogene serine/threonine-protein kinase pim-1-like [Oreochromis niloticus]
eeel_c35699	-	0.414	0.030	123412829	trichohyalin [Trichomonas vaginalis G3]
eeel_c35745	-	0.635	0.011	238624203	centriole cilia and spindle-associated protein [Danio rerio]
eeel_c3589	-	0.660	0.026		
eeel_c36037	-	0.539	0.030	66773118	annexin A13 [Danio rerio]
eeel_c36038	-	0.468	0.017	345490268	PREDICTED: putative acyl-CoA-binding protein-like isoform 2 [Nasonia vitripennis]
eeel_c36694	-	0.593	0.012	499047943	PREDICTED: phospholipase D1-like isoform X1 [Maylandia zebra]
eeel_c36740	-	0.647	0.014	348535818	PREDICTED: uncharacterized protein KIAA0889-like [Oreochromis niloticus]
eeel_c37025	-	0.584	0.030	326679199	PREDICTED: zinc finger protein castor homolog 1 [Danio rerio]
eeel_c3727	-	0.336	0.047	499017584	PREDICTED: tripartite motif-containing protein 35-like [Maylandia zebra]
eeel_c37278	-	0.323	0.014	393715121	MHC class I alpha chain precursor [Oncorhynchus mykiss]
eeel_c37282	-	0.601	0.039	170050090	conserved hypothetical protein [Culex quinquefasciatus]
eeel_c37495	-	0.611	0.012	410931036	PREDICTED: dynein heavy chain 9 axonemal-like [Takifugu rubripes]
eeel_c37562	-	0.324	0.039	291190138	interferon regulatory factor 9 [Salmo salar]
eeel_c37571	-	0.332	0.039	499042356	PREDICTED: uncharacterized protein LOC101486487 [Maylandia zebra]
eeel_c37649	-	0.387	0.039	156551533	PREDICTED: 60S ribosomal protein L32 isoform 1 [Nasonia vitripennis]
eeel_c37655	-	0.577	0.011	410931036	PREDICTED: dynein heavy chain 9 axonemal-like [Takifugu rubripes]
eeel_c37873	-	0.652	0.017		
eeel_c3825	-	0.628	0.016	348500512	PREDICTED: 40S ribosomal protein S28-like isoform 1 [Oreochromis niloticus]
eeel_c38521	-	0.383	0.010	498935505	PREDICTED: ryanodine receptor 3-like [Maylandia zebra]
eeel_c38624	-	0.367	0.021	410924299	PREDICTED: muscleblind-like protein 1-like isoform 9 [Takifugu rubripes]
eeel_c38706	-	0.659	0.039	224157908	predicted protein [Populus trichocarpa]
eeel_c38777	-	0.649	0.030	505778936	PREDICTED: mucin-5AC [Sorex araneus]
eeel_c38821	-	0.514	0.024	242045628	hypothetical protein SORBIDRAFT_02g033135 [Sorghum bicolor]
eeel_c38821	-	0.492	0.047	242045628	hypothetical protein SORBIDRAFT_02g033135 [Sorghum bicolor]
eeel_c38868	-	0.582	0.024	71647202	cytochrome c oxidase subunit I [Japyx solifugus]
eeel_c3894	-	0.437	0.047		
eeel_c39098	-	0.627	0.008	348504506	PREDICTED: leucine-rich repeat-containing protein 16B-like [Oreochromis niloticus]
eeel_c39153	-	0.630	0.030	291393035	PREDICTED: E74-like factor 1-like isoform 1 [Oryctolagus cuniculus]
eeel_c39215	-	0.644	0.021		
eeel_c39258	-	0.615	0.014	125840569	PREDICTED: synaptic vesicle glycoprotein 2A-like isoform 2 [Danio rerio]

eeel_c39491	-	0.436	0.047	348522074	PREDICTED: adenylate kinase isoenzyme 5-like [Oreochromis niloticus]
eeel_c39568	-	0.650	0.039	348510465	PREDICTED: band 4.1-like protein 1-like [Oreochromis niloticus]
eeel_c3997	-	0.465	0.036	261245071	fish virus induced TRIM protein [Oncorhynchus mykiss]
eeel_c40026	-	0.648	0.017	185133512	transport-associated protein 2A [Salmo salar]
eeel_c40081	-	0.617	0.021	223029393	calcium/calmodulin-dependent protein kinase II inhibitor 2 [Danio rerio]
eeel_c40386	-	0.616	0.026	301615430	PREDICTED: calcium-activated potassium channel subunit alpha-1-like [Xenopus (Silurana) tropicalis]
eeel_c4047	-	0.446	0.039	348524998	PREDICTED: dual specificity protein phosphatase 5-like [Oreochromis niloticus]
eeel_c40540	-	0.640	0.024	499019562	PREDICTED: rho GTPase-activating protein 42-like isoform X1 [Maylandia zebra]
eeel_c40759	-	0.608	0.036	291233265	PREDICTED: reverse transcriptase-like protein-like [Saccoglossus kowalevskii]
eeel_c40797	-	0.567	0.013	432953235	PREDICTED: ras suppressor protein 1-like partial [Oryzias latipes]
eeel_c40887	-	0.664	0.047	471229697	proteasome subunit alpha type putative [Ichthyophthirius multifiliis]
eeel_c41033	-	0.594	0.014	432911863	PREDICTED: ankyrin repeat domain-containing protein 12 [Oryzias latipes]
eeel_c41387	-	0.600	0.010	348501742	PREDICTED: receptor-type tyrosine-protein phosphatase epsilon-like [Oreochromis niloticus]
eeel_c41408	-	0.414	0.047	301129246	E3 ubiquitin-protein ligase MARCH7 [Danio rerio]
eeel_c41558	-	0.653	0.017	410897193	PREDICTED: GRIP and coiled-coil domain-containing protein 2-like [Takifugu rubripes]
eeel_c41658	-	0.419	0.011	402854322	PREDICTED: 40S ribosomal protein S8 [Papio anubis]
eeel_c41698	-	0.632	0.036	348528573	PREDICTED: MORC family CW-type zinc finger protein 2A-like [Oreochromis niloticus]
eeel_c41914	-	0.644	0.039		
eeel_c41946	-	0.654	0.024	317031356	reverse transcriptase [Aspergillus niger CBS 513.88]
eeel_c42101	-	0.553	0.000	213510960	Transmembrane protease serine 2 [Salmo salar]
eeel_c42120	-	0.454	0.039	326679617	PREDICTED: hypothetical protein LOC100535411 [Danio rerio]
eeel_c42132	-	0.628	0.011	45433533	eukaryotic translation initiation factor 3 subunit A [Danio rerio]
eeel_c42174	-	0.246	0.012	292621859	PREDICTED: hypothetical protein LOC100331236 [Danio rerio]
eeel_c42351	-	0.442	0.039	326674444	PREDICTED: NACHT LRR and PYD domains-containing protein 14-like [Danio rerio]
eeel_c42470	-	0.598	0.014	41054141	guanine nucleotide-binding protein G(i) subunit alpha-2 [Danio rerio]
eeel_c42491	-	0.578	0.024	71834322	forkhead box protein P2 [Danio rerio]
eeel_c42628	-	0.616	0.012	302856851	hypothetical protein VOLCADRAFT_71691 [Volvox carteri f. nagariensis]
eeel_c42896	-	0.660	0.036	348515953	PREDICTED: serine/arginine-rich splicing factor 4-like isoform 1 [Oreochromis niloticus]
eeel_c43003	-	0.658	0.012	292609536	PREDICTED: G protein-coupled receptor kinase 4-like [Danio rerio]
eeel_c43341	-	0.427	0.021	317031344	zinc knuckle domain protein [Aspergillus niger CBS 513.88]
eeel_c43422	-	0.489	0.026		
eeel_c43451	-	0.506	0.026	190358624	structural maintenance of chromosomes protein 6 [Danio rerio]
eeel_c43576	-	0.410	0.047	348542260	PREDICTED: nuclear factor 7 ovary-like [Oreochromis niloticus]
eeel_c43694	-	0.632	0.026	189536778	PREDICTED: connector enhancer of kinase suppressor of ras 2 [Danio rerio]
eeel_c43751	-	0.462	0.039	190358624	structural maintenance of chromosomes protein 6 [Danio rerio]

eeel_c43851	-	0.436	0.030	499028270	PREDICTED: gamma-interferon-inducible lysosomal thiol reductase-like [Maylandia zebra]
eeel_c43980	-	0.584	0.014	170053960	multicopper oxidase [Culex quinquefasciatus]
eeel_c44006	-	0.632	0.008		
eeel_c44095	-	0.484	0.047	41055726	cyclic AMP-dependent transcription factor ATF-3 [Danio rerio]
eeel_c44177	-	0.572	0.024	213514062	RING finger protein 135 [Salmo salar]
eeel_c44275	-	0.535	0.013	189537632	PREDICTED: formin-binding protein 4 [Danio rerio]
eeel_c44396	-	0.628	0.011	410932505	PREDICTED: protocadherin gamma-C5-like partial [Takifugu rubripes]
eeel_c44442	-	0.417	0.024		
eeel_c44465	-	0.591	0.026	154147642	calcium/calmodulin-dependent protein kinase (CaM kinase) II alpha [Xenopus (Silurana) tropicalis]
eeel_c44482	-	0.645	0.017	326665896	PREDICTED: LOW QUALITY PROTEIN: hypothetical protein LOC565612 [Danio rerio]
eeel_c44605	-	0.437	0.021	348526868	PREDICTED: ras-related protein Rab-13-like [Oreochromis niloticus]
eeel_c45246	-	0.624	0.012	18858935	Kruppel-like factor 4b [Danio rerio]
eeel_c45256	-	0.606	0.026	348508578	PREDICTED: mitotic checkpoint protein BUB3-like [Oreochromis niloticus]
eeel_c45368	-	0.661	0.011	301631257	PREDICTED: general transcription factor II-I repeat domain-containing protein 2-like [Xenopus (Silurana) tropicalis]
eeel_c45439	-	0.499	0.030	410929251	PREDICTED: sodium channel protein type 2 subunit alpha-like partial [Takifugu rubripes]
eeel_c45495	-	0.559	0.017	432867504	PREDICTED: cytochrome c oxidase subunit 6A mitochondrial-like [Oryzias latipes]
eeel_c45611	-	0.465	0.012	292610274	PREDICTED: protein NLRC3 [Danio rerio]
eeel_c45652	-	0.663	0.016		
eeel_c45715	-	0.570	0.014	348534092	PREDICTED: centromere protein F [Oreochromis niloticus]
eeel_c45798	-	0.614	0.030	301616681	PREDICTED: copine-4 [Xenopus (Silurana) tropicalis]
eeel_c4583	-	0.595	0.017	410912876	PREDICTED: homer protein homolog 2-like [Takifugu rubripes]
eeel_c45854	-	0.644	0.047	348536737	PREDICTED: coiled-coil and C2 domain-containing protein 1A [Oreochromis niloticus]
eeel_c45963	-	0.448	0.030	498955662	PREDICTED: ras GTPase-activating protein 1-like [Maylandia zebra]
eeel_c46139	-	0.628	0.047	291401980	PREDICTED: ATP synthase H+ transporting mitochondrial F1 complex gamma subunit [Oryctolagus cuniculus]
eeel_c46178	-	0.449	0.047	301776290	PREDICTED: polypyrimidine tract-binding protein 1-like [Ailuropoda melanoleuca]
eeel_c46241	-	0.538	0.021	156376356	predicted protein [Nematostella vectensis]
eeel_c46455	-	0.582	0.017	41053893	abhydrolase domain-containing protein 2-A [Danio rerio]
eeel_c46480	-	0.647	0.017	327279524	PREDICTED: ankyrin-3-like [Anolis carolinensis]
eeel_c4649	-	0.421	0.026	348511207	PREDICTED: 2-amino-3-ketobutyrate coenzyme A ligase mitochondrial-like [Oreochromis niloticus]
eeel_c46696	-	0.456	0.036	449682231	PREDICTED: uncharacterized protein LOC101237572 [Hydra magnipapillata]
eeel_c46742	-	0.577	0.017	432863525	PREDICTED: RNA-directed DNA polymerase from mobile element jockey-like [Oryzias latipes]
eeel_c46906	-	0.420	0.039	292621744	PREDICTED: NACHT LRR and PYD domains-containing protein 3-like [Danio rerio]

eeel_c46907	-	0.665	0.026	498938576	PREDICTED: regulating synaptic membrane exocytosis protein 2-like isoform X1 [Maylandia zebra]
eeel_c47028	-	0.503	0.047	498987607	PREDICTED: BRISC complex subunit Abro1-like isoform X1 [Maylandia zebra]
eeel_c47501	-	0.657	0.039	348514225	PREDICTED: thiosulfate sulfurtransferase/rhodanese-like domain-containing protein 1-like [Oreochromis niloticus]
eeel_c47809	-	0.485	0.047	465974651	PREDICTED: cysteine and glycine-rich protein 3 [Orcinus orca]
eeel_c48002	-	0.591	0.008	410931036	PREDICTED: dynein heavy chain 9 axonemal-like [Takifugu rubripes]
eeel_c48054	-	0.592	0.026		
eeel_c48150	-	0.575	0.030	432854530	PREDICTED: dynamin-like 120 kDa protein mitochondrial-like [Oryzias latipes]
eeel_c48186	-	0.657	0.039	46309567	immunoglobulin superfamily member 8 precursor [Danio rerio]
eeel_c48218	-	0.469	0.016	66571325	integrin beta-like protein 1 precursor [Danio rerio]
eeel_c4825	-	0.416	0.024	498964349	PREDICTED: serine/threonine-protein kinase 38-like isoform X1 [Maylandia zebra]
eeel_c48303	-	0.342	0.021	326667368	PREDICTED: neoverrucotoxin subunit alpha-like [Danio rerio]
eeel_c48392	-	0.553	0.036	301623875	PREDICTED: solute carrier family 25 member 36 [Xenopus (Silurana) tropicalis]
eeel_c48499	-	0.385	0.016	198278519	transmembrane protein 129 [Rattus norvegicus]
eeel_c4888	-	0.311	0.047	326664998	PREDICTED: hypothetical protein LOC100538086 [Danio rerio]
eeel_c4897	-	0.616	0.024	498949527	PREDICTED: homer protein homolog 2-like isoform X1 [Maylandia zebra]
eeel_c49024	-	0.520	0.017	45387673	hyccin [Danio rerio]
eeel_c4924	-	0.652	0.036	327279490	PREDICTED: probable ATP-dependent RNA helicase DDX5-like [Anolis carolinensis]
eeel_c49389	-	0.652	0.012	348503377	PREDICTED: hypothetical protein LOC100698918 [Oreochromis niloticus]
eeel_c50008	-	0.654	0.026	395535560	PREDICTED: guanine nucleotide-binding protein G(k) subunit alpha [Sarcophilus harrisii]
eeel_c50170	-	0.600	0.024	116517274	proto-oncogene serine/threonine-protein kinase pim-1 [Danio rerio]
eeel_c50422	-	0.528	0.013	348524550	PREDICTED: transmembrane protein 131-like [Oreochromis niloticus]
eeel_c50515	-	0.596	0.013	292617915	PREDICTED: myosin-VIIa-like [Danio rerio]
eeel_c50529	-	0.268	0.026	504156832	PREDICTED: interferon-induced very large GTPase 1-like [Ochotona princeps]
eeel_c50651	-	0.607	0.011	185132733	vascular endothelial growth factor precursor [Oncorhynchus mykiss]
eeel_c51278	-	0.531	0.026	348517988	PREDICTED: synaptotagmin-1-like [Oreochromis niloticus]
eeel_c5145	-	0.621	0.011	348524016	PREDICTED: interferon-induced protein with tetratricopeptide repeats 1-like [Oreochromis niloticus]
eeel_c5149	-	0.661	0.030	432924384	PREDICTED: uncharacterized protein LOC101171703 [Oryzias latipes]
eeel_c51594	-	0.647	0.011	312080142	hypothetical protein LOAG_06891 [Loa loa]
eeel_c51607	-	0.648	0.008	348538677	PREDICTED: dual specificity mitogen-activated protein kinase kinase 5 [Oreochromis niloticus]
eeel_c5565	-	0.217	0.047	393715121	MHC class I alpha chain precursor [Oncorhynchus mykiss]
eeel_c5605	-	0.644	0.026	499020949	PREDICTED: uncharacterized protein LOC101470938 isoform X1 [Maylandia zebra]
eeel_c5883	-	0.208	0.039	393715121	MHC class I alpha chain precursor [Oncorhynchus mykiss]
eeel_c6109	-	0.565	0.036		
eeel_c6170	-	0.521	0.036	318103633	granzyme precursor [Ictalurus punctatus]

eeel_c6273	-	0.530	0.021	380021378	PREDICTED: uncharacterized protein LOC100872060 [Apis florea]
eeel_c6383	-	0.287	0.047	45433507	major histocompatibility complex class I UEA precursor [Danio rerio]
eeel_c6510	-	0.422	0.030	21955148	cytochrome P450 3A18 [Rattus norvegicus]
eeel_c6550	-	0.666	0.026	297293275	PREDICTED: 14-3-3 protein theta isoform 2 [Macaca mulatta]
eeel_c6695	-	0.484	0.011	118350731	Bifunctional endo-14-beta-xylanase xylA precursor putative [Tetrahymena thermophila]
eeel_c6787	-	0.389	0.047		
eeel_c7148	-	0.514	0.030	395742562	PREDICTED: uncharacterized protein LOC100939189 partial [Pongo abelii]
eeel_c7584	-	0.448	0.030	213512927	matrix Gla protein precursor [Salmo salar]
eeel_c7931	-	0.641	0.030	18858707	forkhead box protein G1 [Danio rerio]
eeel_c8188	-	0.632	0.008		
eeel_c8245	-	0.156	0.026	397493737	PREDICTED: uncharacterized protein LOC100978031 partial [Pan paniscus]
eeel_c8247	-	0.636	0.030	213511731	Tumor protein D53 homolog [Salmo salar]
eeel_c8375	-	0.612	0.014	297288028	PREDICTED: zonadhesin [Macaca mulatta]
eeel_c841	-	0.537	0.011	432892790	PREDICTED: betabeta-carotene 9'10'-oxygenase-like [Oryzias latipes]
eeel_c8764	-	0.577	0.008	318103633	granzyme precursor [Ictalurus punctatus]
eeel_c9331	-	0.373	0.036	41055464	coronin-1A [Danio rerio]
eeel_c9337	-	0.514	0.017	395742562	PREDICTED: uncharacterized protein LOC100939189 partial [Pongo abelii]
eeel_c971	-	0.556	0.008	326676757	PREDICTED: hypothetical protein LOC557772 [Danio rerio]
eeel_c9939	-	0.519	0.047	326678171	PREDICTED: nicotinamide riboside kinase 2-like [Danio rerio]
eeel_c9995	-	0.376	0.039	348518447	PREDICTED: hypothetical protein LOC100693528 [Oreochromis niloticus]
eeel_rep_c15977	-	0.538	0.011	498949666	PREDICTED: secretogranin-3-like isoform X2 [Maylandia zebra]
eeel_rep_c15987	-	0.650	0.014	241998968	secreted protein [Ixodes scapularis]
eeel_rep_c16116	-	0.453	0.016	350589594	PREDICTED: MAM and LDL-receptor class A domain-containing protein C10orf112-like partial [Sus scrofa]
eeel_rep_c16180	-	0.640	0.013	432875049	PREDICTED: small G protein signaling modulator 1-like [Oryzias latipes]
eeel_rep_c17503	-	0.553	0.008	145508143	hypothetical protein [Paramecium tetraurelia strain d4-2]
eeel_rep_c18692	-	0.584	0.021		
eeel_rep_c19043	-	0.475	0.014	291394539	PREDICTED: transformer-2 alpha [Oryctolagus cuniculus]
eeel_rep_c19043	-	0.645	0.016	291394539	PREDICTED: transformer-2 alpha [Oryctolagus cuniculus]
eeel_rep_c21853	-	0.622	0.024	170073590	glycosyltransferase PglE [Culex quinquefasciatus]
eeel_rep_c24315	-	0.579	0.017	326681109	PREDICTED: mucin-2-like [Danio rerio]
eeel_rep_c24682	-	0.393	0.024	432950756	PREDICTED: gamma-crystallin M3-like [Oryzias latipes]
eeel_rep_c24987	-	0.482	0.024	185132822	apolipoprotein A-I-2 precursor [Oncorhynchus mykiss]
eeel_rep_c25103	-	0.655	0.008		
eeel_rep_c25103	-	0.487	0.036		
eeel_rep_c26213	-	0.551	0.039		
eeel_rep_c26318	-	0.560	0.008	501595058	hypothetical protein Ycf1 (chloroplast) [Cephalotaxus oliveri]
eeel_rep_c26926	-	0.620	0.026	74095955	ammonium transporter Rh type C 1 [Takifugu rubripes]

eeel_rep_c27289	-	0.493	0.047	301606877	PREDICTED: hypothetical protein LOC100493272 [Xenopus (Silurana) tropicalis]
eeel_rep_c28056	-	0.427	0.012	499017214	PREDICTED: nuclear GTPase SLIP-GC-like [Maylandia zebra]
eeel_rep_c31643	-	0.617	0.021	312100969	hypothetical protein LOAG_13958 [Loa loa]
eeel_rep_c31852	-	0.562	0.010	326679186	PREDICTED: basement membrane-specific heparan sulfate proteoglycan core protein [Danio rerio]
eeel_rep_c31973	-	0.477	0.026	395513797	PREDICTED: LOW QUALITY PROTEIN: MLX-interacting protein [Sarcophilus harrisii]
eeel_rep_c34726	-	0.562	0.012		
eeel_rep_c35338	-	0.596	0.014	358418996	PREDICTED: LOW QUALITY PROTEIN: zonadhesin [Bos taurus]
eeel_rep_c35932	-	0.432	0.011	326666822	PREDICTED: NACHT LRR and PYD domains-containing protein 12-like [Danio rerio]
eeel_rep_c36279	-	0.652	0.039	60279653	anterior gradient protein 2 homolog precursor [Danio rerio]
eeel_rep_c36960	-	0.283	0.039	306966139	MHC class II beta chain precursor [Oncorhynchus mykiss]
eeel_rep_c37243	-	0.626	0.010		
eeel_rep_c38835	-	0.655	0.047	292617919	PREDICTED: papilin isoform 1 [Danio rerio]
eeel_rep_c40098	-	0.527	0.039		
eeel_rep_c45832	-	0.610	0.024	348534997	PREDICTED: lipopolysaccharide-induced tumor necrosis factor-alpha factor homolog [Oreochromis niloticus]
eeel_rep_c48634	-	0.513	0.016		
eeel_rep_c49726	-	0.581	0.014	432854617	PREDICTED: actin-related protein 2/3 complex subunit 5-like [Oryzias latipes]
eeel_rep_c52550	-	0.558	0.017	121583786	TNFAIP3-interacting protein 1 [Danio rerio]
eeel_rep_c53057	-	0.142	0.008		
eeel_rep_c56053	-	0.486	0.030	432867611	PREDICTED: granulins-like [Oryzias latipes]
eeel_rep_c56074	-	0.464	0.008		
eeel_rep_c56340	-	0.620	0.036	380036054	proteasome activator complex subunit 1 [Ictalurus punctatus]
eeel_rep_c56435	-	0.607	0.017	432875404	PREDICTED: uncharacterized protein C8orf4 homolog [Oryzias latipes]
eeel_rep_c56750	-	0.382	0.039	326676527	PREDICTED: barrier-to-autointegration factor [Danio rerio]
eeel_rep_c56817	-	0.449	0.036	157954452	carboxymethylenebutenolidase homolog [Danio rerio]
eeel_rep_c57024	-	0.500	0.012	326668129	PREDICTED: neurogranin isoform 2 [Danio rerio]
eeel_rep_c57180	-	0.399	0.016	318037630	calglandulin [Ictalurus punctatus]
eeel_rep_c57205	-	0.436	0.047	213512214	NADH dehydrogenase 1 beta subcomplex subunit 7 [Salmo salar]
eeel_rep_c57501	-	0.594	0.036	297264978	PREDICTED: striated muscle preferentially expressed protein kinase-like [Macaca mulatta]
eeel_rep_c57923	-	0.563	0.039	185132708	ubiquitin-like protein [Oncorhynchus mykiss]
eeel_rep_c58616	-	0.354	0.047	292627305	PREDICTED: profilin-1 isoform 1 [Danio rerio]
eeel_rep_c58705	-	0.566	0.026	56692314	NADH dehydrogenase subunit 2 [Anguilla anguilla]
eeel_s53036	-	0.538	0.013	499004795	PREDICTED: GTPase IMAP family member 4-like [Maylandia zebra]
eeel_s54326	-	0.584	0.008	302849666	hypothetical protein VOLCADRAFT_97367 [Volvox carteri f. nagariensis]
eeel_s54630	-	0.632	0.030	156381904	predicted protein [Nematostella vectensis]
eeel_s58077	-	0.600	0.014	326536019	NADH dehydrogenase subunit 6 [Hypomesus nipponensis]
eeel_s58164	-	0.249	0.021	348525182	PREDICTED: hypothetical protein LOC100702553 [Oreochromis niloticus]

eeel2_c1043	-	0.518	0.017	348526351	PREDICTED: LOW QUALITY PROTEIN: glyceraldehyde-3-phosphate dehydrogenase-like [Oreochromis niloticus]
eeel2_c1062	-	0.645	0.008	410924588	PREDICTED: 40S ribosomal protein S15-like [Takifugu rubripes]
eeel2_c1202	-	0.644	0.024	348500512	PREDICTED: 40S ribosomal protein S28-like isoform 1 [Oreochromis niloticus]
eeel2_c1221	-	0.472	0.039		
eeel2_c1270	-	0.542	0.011	326676757	PREDICTED: hypothetical protein LOC557772 [Danio rerio]
eeel2_c1331	-	0.539	0.047	410900362	PREDICTED: LOW QUALITY PROTEIN: epithelial cell adhesion molecule-like [Takifugu rubripes]
eeel2_c1355	-	0.280	0.039		
eeel2_c1455	-	0.584	0.024	499008275	PREDICTED: eukaryotic elongation factor 2 kinase-like isoform X1 [Maylandia zebra]
eeel2_c1474	-	0.456	0.000	213515404	RING finger protein 182 [Salmo salar]
eeel2_c154	-	0.440	0.036	410902865	PREDICTED: 40S ribosomal protein S11-like [Takifugu rubripes]
eeel2_c1630	-	0.447	0.010	192447413	THAP domain-containing protein 4 [Danio rerio]
eeel2_c1712	-	0.654	0.014		
eeel2_c1782	-	0.394	0.030	326676399	PREDICTED: protein NLRC3-like [Danio rerio]
eeel2_c1920	-	0.349	0.024	348504506	PREDICTED: leucine-rich repeat-containing protein 16B-like [Oreochromis niloticus]
eeel2_c2084	-	0.388	0.024		
eeel2_c2146	-	0.422	0.047	348503321	PREDICTED: rho GTPase-activating protein 21-like [Oreochromis niloticus]
eeel2_c2159	-	0.523	0.036	410896304	PREDICTED: pleckstrin homology domain-containing family S member 1-like [Takifugu rubripes]
eeel2_c2172	-	0.309	0.024	31795559	troponin T3b skeletal fast isoform 2 [Danio rerio]
eeel2_c2177	-	0.578	0.036	432908770	PREDICTED: tyrosine-protein phosphatase non-receptor type 6-like [Oryzias latipes]
eeel2_c2217	-	0.627	0.012	326918909	PREDICTED: YTH domain-containing protein 1-like isoform 1 [Meleagris gallopavo]
eeel2_c239	-	0.581	0.021	56692324	NADH dehydrogenase subunit 6 [Anguilla anguilla]
eeel2_c244	-	0.573	0.030	213513328	Periphilin-1 [Salmo salar]
eeel2_c2698	-	0.287	0.024	432876129	PREDICTED: uncharacterized protein LOC101164984 [Oryzias latipes]
eeel2_c2747	-	0.628	0.047	260824293	hypothetical protein BRAFLDRAFT_57344 [Branchiostoma floridae]
eeel2_c2765	-	0.620	0.013		
eeel2_c2774	-	0.408	0.008	432860359	PREDICTED: interferon-induced transmembrane protein 5-like [Oryzias latipes]
eeel2_c2977	-	0.350	0.021	185132132	complement factor B [Oncorhynchus mykiss]
eeel2_c3020	-	0.648	0.008	326671325	PREDICTED: CREB/ATF bZIP transcription factor isoform 1 [Danio rerio]
eeel2_c3069	-	0.498	0.026	302847554	hypothetical protein VOLCADRAFT_96261 [Volvox carteri f. nagariensis]
eeel2_c3084	-	0.534	0.047	292622408	PREDICTED: thyroglobulin [Danio rerio]
eeel2_c3155	-	0.617	0.030		
eeel2_c3499	-	0.588	0.021		
eeel2_c3573	-	0.409	0.021	395742562	PREDICTED: uncharacterized protein LOC100939189 partial [Pongo abelii]
eeel2_c3573	-	0.532	0.026	395742562	PREDICTED: uncharacterized protein LOC100939189 partial [Pongo abelii]
eeel2_c3589	-	0.610	0.017	402863056	PREDICTED: zonadhesin-like partial [Papio anubis]
eeel2_c3665	-	0.586	0.011	326676757	PREDICTED: hypothetical protein LOC557772 [Danio rerio]

eeel2_c3732	-	0.573	0.036	348523828	PREDICTED: chromodomain-helicase-DNA-binding protein 3 [Oreochromis niloticus]
eeel2_c3933	-	0.334	0.047	410926025	PREDICTED: proteasome subunit beta type-9-like partial [Takifugu rubripes]
eeel2_c4073	-	0.434	0.017		
eeel2_c4182	-	0.381	0.036	393715121	MHC class I alpha chain precursor [Oncorhynchus mykiss]
eeel2_c434	-	0.551	0.010	259089199	60S ribosomal protein L11 [Oncorhynchus mykiss]
eeel2_c438	-	0.627	0.030		
eeel2_c4606	-	0.214	0.026	498955512	PREDICTED: zinc finger BED domain-containing protein 4-like [Maylandia zebra]
eeel2_c4677	-	0.645	0.012		
eeel2_c4686	-	0.621	0.008	126341092	PREDICTED: girdin-like [Monodelphis domestica]
eeel2_c478	-	0.650	0.047	499030771	PREDICTED: KDEL motif-containing protein 1-like isoform X1 [Maylandia zebra]
eeel2_c4872	-	0.661	0.008	348541079	PREDICTED: hypothetical protein LOC100711790 [Oreochromis niloticus]
eeel2_c579	-	0.352	0.039	66472430	dehydrogenase/reductase SDR family member 7C [Danio rerio]
eeel2_c581	-	0.630	0.024	348524963	PREDICTED: ATP-binding cassette sub-family A member 1-like [Oreochromis niloticus]
eeel2_c590	-	0.632	0.036		
eeel2_c634	-	0.523	0.012	292614216	PREDICTED: neurogranin isoform 3 [Danio rerio]
eeel2_c724	-	0.305	0.008		
eeel2_c724	-	0.490	0.013		
eeel2_c749	-	0.226	0.039	348526351	PREDICTED: LOW QUALITY PROTEIN: glyceraldehyde-3-phosphate dehydrogenase-like [Oreochromis niloticus]
eeel2_c87	-	0.610	0.012	50344746	BET1 homolog [Danio rerio]
eeel2_c906	-	0.654	0.021	348530176	PREDICTED: deoxyribonuclease-2-beta-like [Oreochromis niloticus]
eeel2_rep_c4775	-	0.506	0.047		
eeel2_rep_c5043	-	0.515	0.008	213515404	RING finger protein 182 [Salmo salar]
eeel2_rep_c5055	-	0.551	0.021	499017321	PREDICTED: E3 ubiquitin-protein ligase RNF213-like isoform X1 [Maylandia zebra]
eeel2_rep_c5072	-	0.472	0.017	213515404	RING finger protein 182 [Salmo salar]
eeel2_rep_c5081	-	0.640	0.011		
eeel2_rep_c5117	-	0.560	0.039	441636518	PREDICTED: filaggrin [Nomascus leucogenys]
eeel2_rep_c5357	-	0.612	0.039	50539990	thioredoxin [Danio rerio]
eeel2_rep_c5492	-	0.555	0.010	170073590	glycosyltransferase PglE [Culex quinquefasciatus]
eeel2_rep_c5578	-	0.546	0.014	326676757	PREDICTED: hypothetical protein LOC557772 [Danio rerio]
eeel2_rep_c5590	-	0.193	0.047	395531029	PREDICTED: glyceraldehyde-3-phosphate dehydrogenase-like [Sarcophilus harrisii]
eeel2_rep_c5759	-	0.476	0.039	169403976	glutathione peroxidase 1 [Danio rerio]
eeel2_rep_c5771	-	0.585	0.008	410924037	PREDICTED: 40S ribosomal protein S8-like [Takifugu rubripes]
eeel2_rep_c5827	-	0.487	0.016	113673265	ribosomal protein L22-like 1 [Danio rerio]
eeel2_rep_c5881	-	0.595	0.017	297288028	PREDICTED: zonadhesin [Macaca mulatta]
eeel2_rep_c6070	-	0.368	0.024	318037630	calglandulin [Ictalurus punctatus]
eeel2_rep_c6084	-	0.356	0.039	348544526	PREDICTED: nuclear factor 7 ovary-like [Oreochromis niloticus]
eeel2_rep_c6216	-	0.512	0.008	471413304	PREDICTED: galectin-4 isoform 1 [Trichechus manatus latirostris]

eeel2_rep_c6225	-	0.536	0.024		
eeel2_rep_c6243	-	0.646	0.026	218931124	C-C motif chemokine 28 precursor [Salmo salar]
eeel2_rep_c6407	-	0.658	0.021		
eeel2_rep_c6453	-	0.531	0.011		
eeel2_rep_c6479	-	0.224	0.047	18858227	major histocompatibility complex class II integral membrane alpha chain precursor [Danio rerio]
eeel2_rep_c6530	-	0.624	0.012	156386546	predicted protein [Nematostella vectensis]
eeel2_rep_c6805	-	0.627	0.024	123500541	hypothetical protein [Trichomonas vaginalis G3]
eeel2_rep_c7395	-	0.484	0.024	505786330	PREDICTED: putative uncharacterized protein DDB_G0279653-like [Sorex araneus]
eeel2_rep_c7965	-	0.664	0.036		
eeel2_rep_c8040	-	0.534	0.026	113673265	ribosomal protein L22-like 1 [Danio rerio]
eeel2_rep_c8307	-	0.422	0.016	218931124	C-C motif chemokine 28 precursor [Salmo salar]
eeel2_s5264	-	0.272	0.047	192455680	melanoma-derived growth regulatory protein precursor [Danio rerio]
eeel2_s5351	-	0.286	0.036	192455680	melanoma-derived growth regulatory protein precursor [Danio rerio]
eeel2_s5385	-	0.592	0.024		
eeel2_s5630	-	0.196	0.026	18858227	major histocompatibility complex class II integral membrane alpha chain precursor [Danio rerio]
eeel2_s5640	-	0.623	0.021	66472758	39S ribosomal protein L30 mitochondrial [Danio rerio]
eeel2_s5696	-	0.031	0.047	125838385	PREDICTED: hypothetical protein LOC795173 [Danio rerio]
eeel2_s6082	-	0.557	0.036	432857078	PREDICTED: LOW QUALITY PROTEIN: glutathione peroxidase 1-like [Oryzias latipes]
eeel2_s6121	-	0.654	0.047	395527140	PREDICTED: 60S ribosomal protein L31 isoform 3 [Sarcophilus harrisii]
eeel2_s6284	-	0.291	0.014	301612863	PREDICTED: hypothetical protein LOC100496537 [Xenopus (Silurana) tropicalis]
eeel2_s6374	-	0.640	0.036		
eeel2_s6544	-	0.609	0.026	348521738	PREDICTED: LOW QUALITY PROTEIN: glutathione peroxidase 2 [Oreochromis niloticus]
eeel2_s6934	-	0.450	0.026	169403976	glutathione peroxidase 1 [Danio rerio]
eeel2_s7283	-	0.538	0.047	156358368	predicted protein [Nematostella vectensis]
eeel2_s7960	-	0.564	0.017	261289447	hypothetical protein BRAFLDRAFT_63199 [Branchiostoma floridae]
eeel2_s8066	-	0.213	0.021	18858227	major histocompatibility complex class II integral membrane alpha chain precursor [Danio rerio]
eeel2_s8073	-	0.654	0.010	47174751	non-muscle cofilin 1 [Danio rerio]
eeel2_s8144	-	0.484	0.047	348544526	PREDICTED: nuclear factor 7 ovary-like [Oreochromis niloticus]
eeel2_s8157	-	0.657	0.011		
eeel2_s8193	-	0.637	0.036	62955715	myosin light chain 10 regulatory [Danio rerio]
eeel2_s8225	-	0.591	0.026	498958195	PREDICTED: LOW QUALITY PROTEIN: glutathione peroxidase 1-like [Maylandia zebra]
eeel2_s8233	-	0.647	0.024		
eeel2_s8255	-	0.597	0.036	301612863	PREDICTED: hypothetical protein LOC100496537 [Xenopus (Silurana) tropicalis]
eeel2_s8309	-	0.474	0.030	301612863	PREDICTED: hypothetical protein LOC100496537 [Xenopus (Silurana) tropicalis]
eeel2_s8455	-	0.621	0.036		

eeel2_s8619	-	0.574	0.011	410924037	PREDICTED: 40S ribosomal protein S8-like [Takifugu rubripes]
eeel2_s8627	-	0.612	0.036	47174751	non-muscle cofilin 1 [Danio rerio]
eeel2_s8699	-	0.578	0.024		
