# Supplementary material for: Changes in the gene expression profiles of the brains of male European eels (Anguilla anguilla) during sexual maturation
Source: BMC Genomics. 2014 Sep 17;15(1):799. doi: 10.1186/1471-2164-15-799 (PMC4175612; doi:10.1186/1471-2164-15-799)
Supplement: Supplementary file 5 — Additional file 5: Table S5: Primer sequences for qPCR. List of primer sequences used for qPCR and the expected product sizes in base pairs (bp). Primers were designed to amplify the following differentially expressed genes: galanin peptide (GALP), glutamate decarboxylase 1 (GAD1), corticotropin releasing factor binding protein (CRFBP), C-type natriuretic peptide (CNP), gamma-aminobutyric acid receptor subunit δ (GABRD), neuropeptide Y (NPY), mammalian ependymin-related protein (MERP) and the iroquois-class homeodomain protein 2 (IRX2). The TATA box binding protein (TBP) and Elongation factor 1-alpha 1 (EEF1A1) were used as controls. (PDF 259 KB) [file 12864_2014_6477_MOESM5_ESM.pdf]

Additional file 5: Table S5. Primer sequences for qPCR.

List of primer sequences used for qPCR and the expected product sizes in base pairs (bp). Primers were designed to amplify the following differentially expressed genes: galanin peptide (GALP), glutamate decarboxylase 1 (GAD1), corticotropin releasing factor binding protein (CRFBP), C-type natriuretic peptide (CNP), Gamma-aminobutyric acid receptor subunit δ (GABRD), neuropeptide Y (NPY), mammalian ependymin-related protein (MERP) and the iroquois-class homeodomain protein 2 (IRX2). The TATA box binding protein (TBP) and Elongation factor 1-alpha 1 (EEF1A1) were used as controls.

Gene	Primer sequences (5'-3')	Product size (bp)
GALP	Fw: CAT TCA CAC CAT CAT TGA CTT CC	89
	Rv: ATC TCG TCT GAT GTG ATT GAG G	
GAD1	Fw: CAGCCTTGTTTCTCATTAGCCTGCA	91
	Rv: AAA ATA CAC AGC AAC CAC TTC AG	
CNP	Fw: AAT ACC AAC CTA CCA ACT GTT GC	80
	Rv: TTA AAT ATT ACA ACT CCA CAA CTG G	
CRFBP	Fw: CGC CGA GAG TGG ATT CAG C	138
	Rv: GAT ATA TGA TGG AGA AAC TGC AG	
GABRD	Fw: ATC GAC GCC GAC ACC ATC G	100
	Rv: GCC TCA CAT GGT GTA CGC C	
NPY	Fw: CTT ACT GTC AGC TAC GTG CC	113
	Rv: GGA GCA GTT TGT ATG GAC AGC	
MERP	Fw: CTG GGT CTG AAT TCT TGA TTC G	90
	Rv: CGC AAA TAC ACA GCG TCT CC	
IRX2	Fw: TGTGCATTTGTTGGTAGTTTCCC	145
	Rv: TCT TAC AAA TAA GCT TTA CAG TAG G	
TBP	Fw: GAG TTG CGA CGT GAA GTT CC	132
	Rv: AAT ATC AAG AGC ACA ATC CTG GG	
EEF1A1	Fw: CGA GAA GTT CGA GAA GGA AGC	141
	Rv: TAC TTG GTG GTC TCA AAC TTC C	
